# Abstract Proceedings of the 38th Annual Canadian Association of Psychosocial Oncology (CAPO) Conference, June 2023

**DOI:** 10.3390/curroncol30070503

**Published:** 2023-07-20

**Authors:** Peter Traversa, Carmen G. Loiselle

**Affiliations:** 1 Canadian Association of Psychosocial Oncology, Toronto, ON M5A 1S2, Canada; 2 Departments of Oncology and Ingram School of Nursing, McGill University, Montreal, QC H3A 2M7, Canada; carmen.g.loiselle@mcgill.ca

**Keywords:** psychosocial, oncology, cancer, research, co-design

## Abstract

On behalf of the Canadian Association of Psychosocial Oncology, we are pleased to present the Abstracts from the 2023 Annual Conference, titled “Co-designing Psychosocial Oncology: Optimizing Outcomes for All”. The conference was held in Montreal from 20 June 2023 to 22 June 2023. This conference brought together key stakeholders including multidisciplinary professionals from nursing, psychology, psychiatry, social work, spiritual care, nutrition, medicine, rehabilitation medicine, occupational health and radiation therapy for both adult and pediatric populations. Participants included clinicians, researchers, educators in cancer care, community-based organizations and patient representatives. Patients, caregivers and family members presented abstracts that spoke to their role in managing cancer experiences and care. Over one hundred (100) abstracts were selected for presentation as symposia, 20-min oral presentations, 10-min oral presentations, 90-min workshops and poster presentations. We congratulate all the presenters on their research work and contribution.

## 1. Abstract Themes

A. Equity, diversity, inclusion and advocacy in cancer care and research
B. Cancer care across the life span (children, adolescent and young adults, adults, and older adults)
C. Complementary and integrative cancer care
D. Community-based and volunteer cancer-care services
E. Sociodemographic, culture, and sex/gender issues in cancer
F. Digital health and cancer care
G. Exercise/pre-habilitation and rehabilitation in cancer
H. Implementation science, knowledge translation and synthesis
I. Survivorship
J. Palliative and end-of-life care
K. Primary, secondary and tertiary cancer prevention
L. Innovation in psychosocial oncology interventions
M. Health care provider wellness
N. Cancer treatment-related symptom and toxicity management
O. Pandemics and cancer care issues
P. Adapting PSO care in LMI countries
Q. Patient-oriented research approaches
R. Other value-based and person-centered cancer care

## 2. Symposia

### 2.1. S1. Improving Psychosocial Support for Adolescents and Young Adults with Cancer: Moving from Unmet Needs to Innovative Interventions

Moderator: Fiona Schulte

Summary: Adolescents and young adults (AYAs) with cancer are increasingly recognized as a population with distinct medical and psychosocial needs, which are often unmet and overlooked. Despite the growing body of research on AYA oncology, knowledge gaps remain and contribute to ongoing challenges with care. For instance, there is a limited understanding of the experiences of AYAs with diverse identities and a lack of evidence-based interventions that address the specialized psychosocial needs of AYAs. In this symposium, speakers will present new research co-designed with patient partners focused on improving the psychosocial care for all AYAs with cancer. First, J. Duong and I. Rahamatullah will co-present data on barriers and enablers to engaging AYAs who have been historically under-represented in cancer research (e.g., patients who identify as racialized, 2SLGBTQIA+, and with intersecting under-represented identities) with a focus on recommendations for tailoring interventions to optimize inclusivity. Next, P. Tutelman will describe patient perspectives of a new group-based psychotherapeutic intervention for AYAs between the ages of 16 and 29 years. Finally, D. Male will outline the development of ‘PIVOT’, a novel intervention focused on addressing the unique needs of older AYAs in their 30s and 40s. Implications for future research and clinical practice will be discussed.

S1-34

### 2.2. Prioritizing Important Values, Opportunities and Transitions: Preliminary Results of the PIVOT Group Intervention for People in Their 30s and 40s Navigating the Ways Cancer Changes Life Plans

Dana Male ^1,2^, Sara Beattie ^1,2^, Perri Tutelman ^1,2^, Fiona Schulte ^1,2^, Jennifer Pink ^3^, Jessie Moorman ^2^ and April Boychuk ^2^

1. University of Calgary, Calgary, Canada
2. Alberta Health Services, Calgary, Canada
3. University of Waterloo, Waterloo, Canada

Background/Rationale or Objectives/Purpose

Cancer often impacts significant life plans (e.g., career, relationships, family planning) for people in their 30s and 40s. Individuals face loss, disruption, isolation, and report challenges accessing well-matched resources (e.g., lack of belonging in available programs, typically attended by older adults or adolescents and young adults as young as 18 years old). To address the psychosocial needs of these individuals, clinicians at the Tom Baker Cancer Centre (TBCC) developed and piloted a six-week ‘PIVOT’ therapy group for people aged 30–49.

Methodology or Methods

Eligible participants were either receiving active treatment, or had completed treatment ≤ five years, through the TBCC. PIVOT is founded on acceptance and commitment therapy (ACT) and supportive expressive therapy (SET) principles. Sessions focused on a particular domain (e.g., body image, relationships, sexual health, career/livelihood) and incorporated psychoeducation and discussion. Baseline, post-treatment, and follow-up questionnaires were collected, including treatment acceptability data.

Impact on Practice or Results

Eight people (five females and three males, aged 36–47) participated in the first cohort. Primary tumour sites included breast, lymphoma, cervical, ovarian, melanoma, testicular. Three participants had a metastatic cancer diagnosis and five were in active treatment. Sessions were well attended (88–100% attendance). Satisfaction ratings were favourable (100% agreed they would recommend the group to a friend).

Discussion or Conclusions

Findings suggest a group of this kind is acceptable and feasible for people in a similar life stage despite other varying factors (e.g., gender identity, tumour type, cancer stage) and significant disease burden (e.g., active treatment). Future directions include ongoing implementation and evaluation with goals of group manualization and integration into standard psychosocial care.

S1-41

### 2.3. “I Didn’t Realize How Much I Needed the Connection until I Began the Group”: Participant Perspectives of an Online Group Psychotherapy Intervention for Adolescents and Young Adults with Cancer

Perri Tutelman ^1,2^, Chelsea Moran ^1^, Sara Beattie ^1,2^, Melanie Khu ^2^, Melissa Howlett ^3^, Kristen Silveira ^4^ and Fiona Schulte ^1,2^

1. University of Calgary, Calgary, Canada
2. Alberta Health Services, Calgary, Canada
3. The Hospital for Sick Children, Toronto, Canada
4. Vancouver Island Health Authority, Victoria, Canada

Background/Rationale or Objectives/Purpose

There is growing recognition that specialized, developmentally tailored interventions are needed to address the unmet psychosocial needs of adolescents and young adults (AYAs) with cancer. The objective was to explore participant perspectives of a novel online group psychotherapy intervention for AYAs.

Methodology or Methods

The intervention was a manualized virtual psychotherapy group consisting of eight, weekly sessions facilitated by trained therapists. It was co-designed with patients to address AYA-specific psychosocial needs, promote peer interaction and bolster coping skills. Participants were AYAs with cancer (currently 16–29 years, diagnosed ≥ 15 years) who were treated at the Alberta Children’s Hospital or Tom Baker Cancer Centre in Calgary, AB. To be eligible, patients had to be currently receiving treatment or be within 5 years of treatment completion. After completing the group, participants answered an open-ended question on the most and least enjoyable aspects of the group. Responses were analyzed using content analysis.

Impact on Practice or Results

Thirty-four AYAs forming four groups completed the intervention. Twenty-seven (79%) provided open-ended survey responses. The most enjoyable aspects of the group were summarized by the following categories: (1) group discussion; (2) peer connection and support; and (3) increased comfort with self-expression. Least enjoyable aspects and suggestions for improvement clustered into the following areas: (1) delivery method (preference for in-person vs. virtual); (2) length (preference for longer vs. shorter); and (3) format (preference for more vs. less structure).

Discussion or Conclusions

The results offer empirical data on a novel online group psychotherapy intervention for AYAs with cancer. Findings will guide intervention refinement to support ongoing clinical programming for this population.

S1-52

### 2.4. Exploring Lived Experiences and Inclusive Engagement Practices for Under-Represented Adolescents and Young Adults in Psychosocial Oncology Research: A Qualitative Study

Jenny Duong ^1^, Caitlin Forbes ^1,2^, Brianna Henry ^1,2^, Tristan Bilash ^1^, Iqra Rahamatullah ^1^, Sharon Hou ^1,3^, Sheila Garland ^4,5^, Jackie Bender ^6,7^ and Fiona Schulte ^1,2^

1. University of Calgary, Calgary, Canada
2. Alberta Children’s Hospital, Calgary, Canada
3. British Columbia Children’s Hospital, Vancouver, Canada
4. Memorial University, St. John’s, Canada
5. Beatrice Hunter Cancer Research Institute, Halifax, Canada
6. Department of Supportive Care, Princess Margaret Cancer Centre, Toronto, Canada.
7. Dalla Lana School of Public Health, University of Toronto, Toronto, Canada

Background/Rationale or Objectives/Purpose

What we know about survivors of adolescent and young adult (AYA) cancer is limited by the under-representation of AYAs who identify as Indigenous, racialized, 2SLGBTQIA+, and other social identities. The aim of this project was to identify barriers and enablers to engaging under-represented AYAs in research in inclusive ways.

Methodology or Methods

Eligible participants were AYAs aged 15–39 diagnosed with cancer between the ages of 15 and 39, who self-identified as under-represented and lived in Canada. Stakeholders were also eligible if they advocated for Canadian AYAs with cancer from under-represented groups. The study occurred virtually and recruited from across Canada.

Participants were recruited through social media, snowball and convenience sampling, and promotions by partnered community organizations. Interviews were conducted over Zoom videoconferencing, audio-recorded, and transcribed verbatim. A hybrid deductive–inductive thematic analysis approach was used by two independent coders. Patient and community partner feedback informed coding and theme generation.

Impact on Practice or Results

Interviews with AYAs (*n* = 4) and stakeholders (*n* = 2) were conducted. Most AYAs identified as racialized and had intersecting identities. Preliminarily identified barriers to engagement included lack of community, lack of information, patient confidentiality, and stigma. Preliminary enablers included representation, intersectionality, online platforms for accessibility and outreach, reciprocity, and systemic change.

Discussion or Conclusions

These findings will inform future studies on AYA cancer, especially online studies facing novel challenges regarding participant safety and data integrity. This study also amplifies the voices of under-represented AYAs to ensure they are effectively included in research along the cancer care continuum.

### 2.5. S2 CAPO and CANO Aligning Priorities: The Power of Conversation, Collaboration, and Co-Creation

Moderator: Carmen Loiselle

Summary: As two key Canadian organizations committed to enhancing experiences and outcomes for people affected by cancer, the Canadian Association of Psychosocial Oncology (CAPO) and the Canadian Association of Nurses in Oncology (CANO) have undertaken collaborative processes to optimize our strategic plans. In this symposium, we discuss how CAPO and CANO independently and together push our organizations’ agendas forward. Through three presentations, we (1) introduce our respective vision, mission, and priorities, (2) identify promising trends including value-based healthcare that can benefit from fuller integration within and across organizations, and (3) present a concrete example, the I-CAN Manage Program that underscores how innovation co-design and integration can readily be achieved. The symposium concludes with a discussion on next steps to further collaboration, creativity, and innovation as essential components of co-creation.

S2-119

### 2.6. CAPO/ACOP’s Strategic Objectives for Optimizing Health and Healing among Individuals Affected by Cancer

Carmen Loiselle

McGill University, Montreal, Canada

Background/Rationale or Objectives/Purpose

The Canadian Association of Psychosocial Oncology (CAPO/ACOP) seeks to advance the field of psychosocial oncology for the benefit of patients and family members. The 2022–2027 strategic plan reflects its vision of “psychosocial care that optimizes health and healing for every Canadian affected by cancer”.

Methodology or Methods

This is to be achieved through CAPO’s mission “to foster the development, testing, implementation and sustainability of best practices in psychological, social, physical, and spiritual well-being among individual affected by cancer”.

Impact on Practice or Results

Partnerships and interprofessional collaborations are crucial as we face significant cancer-care delivery challenges and reforms. Joining efforts with diverse cancer-related organizations will ensure more comprehensive models of care to better support all affected by cancer including patients, caregivers, and under-represented communities.

Discussion or Conclusions

CAPO continues to be committed to strong collaborations across cancer-related organizations including close partnership with the Canadian Association of Nurses in Oncology (CANO). Within this context, concrete joint initiatives are explored moving forward, as well as new opportunities and anticipated challenges. Key priorities pertaining to innovation and education, research and knowledge translation, clinical and community care, as well as leadership and advocacy will also be presented.

S2-120

### 2.7. CANO/ACIO’s Strategic Orientations for Enhanced Access and Equity in Cancer Care

Joy Tarasuk

Nova Scotia Health, Halifax, Canada

Background/Rationale or Objectives/Purpose

The Canadian Association of Nurses in Oncology (CANO/ACIO) seeks “to advance cancer care nursing through advocacy, collaboration, the provision of practice resources, research and leadership for the benefit of all people living with cancer”. The 2021–2025 priorities reflect its vision of being “a recognized leader in pursuing cancer care nursing excellence and improving access and equity, nationally and internationally”.

Methodology or Methods

More specifically, CANO strives to engage rural/remote nurses to understand and respond to the unique educational and practical needs of these practice settings, including virtual care, advocacy, and evidence-informed practice guidelines.

Impact on Practice or Results

A focus on equitable care access and systematic documentation of patient-reported outcomes can translate into more tailored support for patients and informal caregivers. Likewise, a better understanding of oncology nurses’ experiences can assist in addressing workplace stressors while increasing cancer care team efficacy and efficiency.

Discussion or Conclusions

CANO recognizes that, to meet these goals, close partnership with other organizations such as CAPO is essential. Through collaboration and co-creation, our two organizations can work together toward common goals to ensure comprehensive cancer care that places patients and families at the center of these processes. Together, CANO/CAPO can act as catalysts for innovative models of cancer care delivery, such as I-CAN—presented in this symposium—that truly integrate interprofessional practice, education/training, research, leadership, and advocacy.

S2-121

### 2.8. Giving Voice to Patients in Codesigning Cancer Care Innovations: The I-CAN Manage Web-Based Self-Management Program

Doris Howell

University Health Network, Toronto, Canada

Background/Rationale or Objectives/Purpose

Transformations in cancer care can be realized through mobilizing psychosocial and nursing resources across our organizations and, most importantly, through giving voice to patients (and families) in the co-design of care-related innovations. In this presentation, we highlight the importance of co-design, its principles and practice, and provide an example of how co-design was used to develop a digital self-management support program.

Methodology or Methods

A user-centred co-design process was used in partnership with patients and other knowledge end-users to develop and iteratively test a theoretically informed web-based cancer self-management education program prototype (I-CAN Manage). The prototype content, features, and functions were informed by an initial qualitative inquiry discovery phase followed by personal and journey mapping with patients and knowledge end-users. The I-CAN Manage program integrates information and evidence-based strategies for self-management of cancer and treatment. Behavioral exercises, patient written and video stories, downloadable learning resources, and online completion of brief SMART (specific, measurable, achievable, relevant, time-bound) goals and action plans were integrated throughout.

Impact on Practice or Results

The program principally targets patient activation in the use of core self-management skills, problem-specific self-management strategies, and health behaviours to manage cancer and its treatment.

Discussion or Conclusions

I-CAN Manage was tested in a feasibility trial combined with coaching delivered by oncology nurses and a signal for improvement in patient activation was obtained. I-CAN development and testing will be presented as a promising exemplar of cross-disciplinary and patient co-design.

### 2.9. S3 Integrating AYA Cancer Care in Canada

Moderator: Jonathan Avery, Tiffany Hill

Summary: It is now widely acknowledged that adolescent and young adults (AYAs), individuals between 15 and 39 years of age, diagnosed with cancer, require specific and tailored oncological and supportive cancer care. Provision of AYA-specific care has become standard in publicly funded health systems such as the National Health Service (NHS) in the UK and in Australia. However, in Canada, no such sustainable funding exists, which has left many oncology programs from across provincial jurisdictions to find their own way to integrate evidenced-informed AYA cancer care into the existing clinical infrastructure. Cancer care is provincially regulated, and each province would require a specific implementation strategy to incorporate AYA care to the existing infrastructure. The purpose of the symposium is to hear from three different groups from across Canada (groups from BC, Manitoba, and Ontario) on their efforts and learnings to establish and integrate AYA cancer care into clinical practice.

S3-131

### 2.10. Co-Designing a New Young Adult Cancer Program at BC Cancer

Cheryl Heykoop ^1^ and Jonathan Avery ^1,2^

1. Anew Research Collaborative: Reshaping Young Adult Cancer Care, Royal Roads University, Victoria, Canada
2. Department of Supportive Care, Princess Margaret Cancer Centre, University Health Network, Toronto, Canada

Background/Rationale or Objectives/Purpose

It is well established that adolescent and young adults (AYAs), individuals between 15 and 39 years of age, require specific oncologic and supportive care distinct from children and older adults. There is currently no provincial AYA program in British Columbia; thus, there is an unmet need.

Methodology or Methods

The Anew Research Collaborative was formed to reshape AYA cancer care in BC. Using participatory, patient-oriented, and action-focused research approaches, we are working with BC Cancer to co-design a provincial AYA cancer care program with AYAs, cancer care providers, supporters, and decision-makers. To date, we have facilitated creative dialogues with AYAs, interviewed providers, reviewed policies, and engaged in ongoing dialogue with decision-makers. We are also co-developing an immersive, multi-sensory experience to share unique AYA cancer care experiences.

Impact on Practice or Results

Together, we are co-creating a provincial AYA program that responds to the unique needs of AYAs, their supporters, and cancer care providers across BC. Throughout the process we are working to intentionally ensure AYAs with diverse, intersectional identities are meaningfully involved and we are co-creating knowledge translation outputs to transform cancer care for AYAs in BC and beyond.

Discussion or Conclusions

As co-development efforts with AYAs, providers, supporters, and decision-makers are underway, we are hopeful that our collective work will develop a comprehensive, responsive, and sustainable AYA program in BC that reshapes cancer care for AYAs.

S3-132

### 2.11. Development of a Multidisciplinary Adolescent and Young Adult Program at CancerCare Manitoba: Lessons Learned from A Mid-Sized Canadian Oncology Centre

Ian Scott ^1^, Mackenzie Jansen ^2^, Caitlin Woodrow ^3^, Lorena Gerl ^3^, Sapna Oberoi ^4,5^, Anne Katz ^2^ and Ruth Loewen ^6^

1. Department of Psychosocial Oncology, CancerCare Manitoba, Winnipeg, Canada
2. Department of Nursing, CancerCare Manitoba, Winnipeg, Canada
3. Department of Patient and Family Support Services, CancerCare Manitoba, Winnipeg, Canada
4. CancerCare Manitoba Research Institute, CancerCare Manitoba, Winnipeg, Canada
5. Section of Pediatric Hematology-Oncology, CancerCare Manitoba, Winnipeg, Canada
6. Department of Community Oncology, CancerCare Manitoba, Winnipeg, Canada

Background/Rationale or Objectives/Purpose

A multidisciplinary program providing personalized care to adolescents and young adults (AYAs) with cancer is the most effective way to meet their complex needs. Such a program would provide comprehensive wrap-around care and greatly improve healthcare experiences and outcomes for AYAs.

Methodology or Methods

In 2022, the AYA program at CancerCare Manitoba (CCMB) received funding from the CCMB Foundation to expand its staffing complement beyond the existing psychosocial clinician position. The grant included funding for a 1.0 FTE Clinical Nurse Specialist (CNS), 0.5 FTE Dietitian, 1.0 FTE Clinical Research Coordinator, 0.5 Secretary, 0.4 FTE Physiotherapist, 0.3 FTE Occupational Therapist and 0.2 FTE Physiatrist.

CCMB’s AYA program includes a sustainable research platform linked with the CCMB Research Institute. The vision is for research to help ameliorate outcomes for AYAs with cancer and their families.

Impact on Practice or Results

The AYA CNS and dietitian positions have been filled. Activities thus far include staff education, initiation of an environmental scan, and the development of a standardized and universal referral pathway. The dietitian has seen a steady increase in referrals. There is an ongoing effort to fill the remaining positions in the AYA grant. The research platform continues to evolve, including the development of AYA metrics, patient engagement activities and program evaluation.

Discussion or Conclusions

CCMB is a mid-sized Canadian oncology centre that sees an estimated 300 new AYA patients annually. Its AYA program could serve as a blueprint for other jurisdictions. Through a robust multidisciplinary approach, the goal is to be a national leader in AYA oncology.

S3-133

### 2.12. Building an AYA Patient Community: Highlighting the Princess Margaret Cancer Centre’s Initiatives and Their Impact on Reducing Social Isolation

Luxshiga Premakumar, Sharon D’Souza, Marlie Smith, Abha Gupta

Department of Supportive Care, Princess Margaret Cancer Centre, University Health Network, Toronto, Canada

Background/Rationale or Objectives/Purpose

The adolescent and young adults (AYA) program at PM provides personalized supportive care to young adults between the ages of 18 and 39 with cancer. AYAs comprise only 10% of the adult cancer population and unique challenges based on their developmental stage; some challenges include fertility, sexuality, body image, school/work transitions, and fostering connections with their social network. The resulting isolation has a detrimental impact on their mental health.

Methodology or Methods

Our program’s clinical nurse specialist provides one-on-one support to referred patients. Through these consults innovative ways of engaging patients have arisen to foster a strong patient community. In collaboration with our team, new patient events were hosted, including cooking classes, art therapy, yoga classes, book club and socials. Another key component that emerged from discussions with patients is assistance with transitioning to school/work.

Impact on Practice or Results

With greater event programming, we have fostered an AYA community consisting of over 100 patients. The community continues to grow with a strong social media presence of over 800 followers across platforms. Efforts have also been directed to spread awareness to physicians for increased referrals. Patients are now able to engage with other patients, attend in person/virtual events, rebuild their career path, access school support, and minimize disruptions to their education/employment goals.

Discussion or Conclusions

AYAs with cancer have expressed a desire for more opportunities to engage with other patients with shared lived experiences. Future directions include collecting patient-reported outcomes to guide patient-centered programming and develop strategies to sustain the program over time.

## 3. Final Category: A. Equity, Diversity, Inclusion and Advocacy in Cancer Care and Research

12

### 3.1. Value of Having Patient Advisors in Committees: Experience of the Canadian Association of Psychosocial Oncology (CAPO) Advocacy Committee

Sevtap Savas ^1^, Chantale Thurston ^2^, Tristan Bilash ^3^ and Kimberley Thibodeau ^4^

1. Memorial University, CAPO Advocacy, St-John’s, Newfoundland and Labrador, Canada
2. CAPO Advocacy Committee, Manitoba, Canada
3. CAPO Advocacy Committee, Saskatchewan, Canada
4. McGill University Health Center, Montreal, Canada

Background/Rationale or Objectives/Purpose

Background: The voices of cancer-affected individuals (“patient advisors”) are critical in driving meaningful change and effectively addressing their priorities and needs. The Canadian Association of Psychosocial Oncology (CAPO) has a keen interest in working with patient advisors and integrating their perspectives in its activities, operations, and organizational culture.

Objectives: Our aim is to share the experience and perspectives gained by working with patient advisors recruited to the Advocacy Committee of CAPO (CAPO-AC).

Methodology or Methods

This is a reflective work. The committee members reflected on their own experience regarding the factors influencing recruitment of patient advisors and their participation in committee meetings. Additionally, they reviewed the meeting minutes and email communications in relation to the impact and contributions of patient advisors. Last, a meeting was held focusing on a patient advisor’s perspectives and experience with the CAPO-AC committee.

Impact on Practice or Results

Results: Our committee recruited three patient advisors in 2022. Patient advisors have been enthusiastic contributors and critical in hearing diverse patient and family priorities, opinions, and interests. These led to the identification of new patient-oriented strategic objectives that were communicated to the CAPO leadership. Time zone differences, CAPO membership requirements, and workplace requirements were among the barriers for participation. Our committee has taken steps to remove some of these barriers.

Discussion or Conclusions

Conclusion: Patient advisors make important contributions to CAPO-AC’s mandates. Removing the barriers for participation is critical, empowers all sides, can increase representation of cancer-affected individuals in CAPO and other organizations, and eventually can facilitate meaningful change in the lives of cancer-affected individuals.

20

### 3.2. Access to Symptom Screening and Severe Symptom Risk among Cancer Patients with Severe Psychiatric Illness

Laura E. Davis ^1^, Rinku Sutradhar ^2,3^, Michaela A. Bourque ^4^, Antoine Eskander ^2,3^, Natalie Coburn ^2,3^, Julie Deleemans ^5^, Wing C. Chan ^2^, Julie Hallet ^2,3^ and Alyson L. Mahar ^2,6^

1. McGill University, Montreal, Canada
2. ICES, Toronto, Canada
3. University of Toronto, Toronto, Canada
4. Queen’s University, Kingston, Canada
5. University of Calgary, Calgary, Canada
6. Queen’s University, Toronto, Canada

Background/Rationale or Objectives/Purpose

Objectives/Purpose: Cancer patients with severe psychiatric illness (SPI) are at higher risk of health inequalities. We evaluated the rates of symptom screening and burden using the Edmonton Symptom Assessment System (ESAS) in cancer patients with SPI.

Methodology or Methods

Sample and Setting: Linked administrative databases in Ontario, Canada were employed to conduct a retrospective cohort study of persons diagnosed with cancer from 1 January 2007 to 31 August 2020. The sample comprised 4049 inpatients and 9775 outpatients with SPI, and 376,046 individuals who had neither SPI nor recently utilized mental health services (total *n =* 389,870).

Procedures: SPI was assessed across five years preceding cancer diagnosis and classified as inpatient, outpatient, or no SPI. Outcomes were defined as ≥1 ESAS screening and ≥1 moderate-to-severe symptom (severe score ≤ 4 per symptom) among those who completed ≥1 ESAS assessment. Cause-specific and Fine and Gray competing events models were used for each outcome.

Impact on Practice or Results

Results: Individuals with inpatient or outpatient SPI had significantly lower incidence of routine symptom screening records compared to those without SPI after accounting for the competing risk of death (multivariable sHR 0.77 (95%CI 0.74–0.80) and 0.88 (95%CI 0.86–0.90)). Additionally, individuals living with inpatient or outpatient SPI were significantly more likely to experience severe symptoms compared to those without an SPI.

Discussion or Conclusions

Conclusion and Clinical Implications: Patients with inpatient and outpatient SPI are less likely to be ESAS evaluated but also have higher symptom scores. Understanding disparities in ESAS screening for SPI and cancer patients is essential to ensuring equitable cancer care.

22

### 3.3. Financial Aid Requests by Brain Tumor Patients in Ontario through GoFundMe

Kaviya Devaraja ^1^, Yajur Iyengar ^1^, Andy Zhang ^1^, Jonathan Avery ^2^ and Seth Climans ^3^

1. University of Toronto, Toronto, Canada
2. Princess Margaret Cancer Center, Toronto, Canada
3. London Health Sciences Center, Toronto, Canada

Background/Rationale or Objectives/Purpose

Objective/purpose: Numerous Ontario brain tumor patients suffer the unequal burden of responsibility of paying for treatments like oral chemotherapy. However, less is known about other direct and indirect financial costs due to their diagnosis. The purpose of this study was to analyze publicly available data from GoFundMe, an online fundraising platform, to explore the financial needs of brain tumor patients.

Methodology or Methods

Method: A qualitative descriptive design drawing on thematic analysis was used to analyze GoFundMe requests to support individuals diagnosed with brain cancer in Ontario between 2014 and 2021. A coding framework was created to determine emerging themes using qualitative data analysis software NVivo 10.

Impact on Practice or Results

Results: There were 195 fundraising requests. Requests described financial strain from the loss of income experienced by the patient and their caregivers to afford cancer treatments. Requests highlighted (1) an overall lack of awareness of how and where access financial aid and affordable psychosocial support; (2) concerns over the long-term financial well-being of bereaving family members; (3) A call for more public awareness of the financial burden and emotional distress experienced by those impacted by brain cancer.

Discussion or Conclusions

Conclusions/clinical implications: These GoFundMe requests highlight a connection between financial burden and emotional distress and an overall lack of awareness of where/how to seek financial and emotional support. These results will serve as the foundation to advocate for and raise awareness of the financial assistance needed by brain tumor patients and their families.

30

### 3.4. Timely and Equitable Access to Psychosocial Oncology

Marianne Arab, Lindsay Dickson

NS Health, Halifax, Canada

Background/Rationale or Objectives/Purpose

Patient access to the Psychosocial Oncology Team at NS Health was challenging with long wait times to see some disciplines. There was inequity and inconsistency in the service provision. The project sought to develop an innovative model to deliver care when, where and how it was needed. Objectives were to ensure that all patients living with cancer have equitable access to high-quality, person-centered care and to build a healthy, satisfied and sustainable workforce.

Methodology or Methods

A needs assessment was completed with patients living with cancer. The team engaged with healthcare providers who were part of the PSO Team, referral sources and the senior management of the Cancer Care Program. Interventions:

1. Develop and implement a centralized referral process that outlined the criteria for referral, including consistent processes to receive, track and triage referrals and monitor wait times;
2. Develop practice standards and guidelines for each discipline that included standardized assessments and documentation processes, discipline-specific profiles based on core discipline competencies and guidelines for service duration and frequency of treatment.

Impact on Practice or Results

As a result of this quality improvement project, the PSO team provides centralized, equitable and accessible patient-centered care in an innovative, coordinated and efficient manner compared to the previous model. Patients are now experiencing decreased wait times and equitable access.

Discussion or Conclusions

The PSO program now continuously monitors program quality through the collection of key performance indicators and patient satisfaction data.

A key lesson learned during this project was the importance of engaging patients to identify their needs and help shape the services they receive.

32

### 3.5. Lived Experience of Black Women with Breast Cancer in Toronto, Canada

Ielaf Khalil ^1^, Gayathri Naganathan ^2^, Aisha Lofters ^2,3^, Juliet Daniel ^4^, Leila Springer ^5^, Frances Wright ^2,6^, Danielle Rodin ^2,7^, Tulin Cil ^2,7^, Olive Wahoush ^8^ and Andrea Covelli ^1,2^

1. Sinai Health System, Toronto, Canada
2. University of Toronto, Toronto, Canada
3. Women’s College Hospital, Toronto, Canada
4. McMaster University, Hamilton, Canada
5. The Olive Branch of Hope, Toronto, Canada
6. Sunnybrook Health Sciences Centre, Toronto, Canada
7. University Health Network, Toronto, Canada
8. McMaster University, Toronto, Canada

Background/Rationale or Objectives/Purpose

Although little is known in the Canadian context, there are well-documented differences in treatments and outcomes for Black women undergoing breast cancer care in the United States. Canada is not immune to racial disparities, but race-based health data are not routinely collected. The objective of this project was to broaden our understanding in the Canadian context of how Black women experience cancer and to identify barriers and inadequacies in care.

Methodology or Methods

One-on-one, semi-structured qualitative interviews were conducted with 30 women living in Toronto, Ontario, Canada who identified as Black who were currently undergoing or had previously undergone treatment for breast cancer. Data were analyzed using an inductive, constant comparative method to derive themes.

Impact on Practice or Results

There are unique needs and challenges that Black women in Toronto experience during their breast cancer treatment. Seven main themes were identified: (1) mistrust of the healthcare system; (2) microaggressions and lack of representation; (3) need for psychosocial support; (4) dismissal of pain; (5) fertility considerations; (6) financial strain; and (7) importance of faith and community. Recommendations included the importance of patient advocacy, and the need for race-based data and racially concordant care.

Discussion or Conclusions

Black women with breast cancer in Toronto, Canada face multiple challenges that appear to be rooted in prejudice and systemic racism. Further research is needed to develop tools to address these inequities and close the gap in care.

55

### 3.6. Co-Creation of a National Agenda for Psychosocial Oncology Advocacy: Ongoing Partnerships and Updates

Samar Attieh ^1^, Sevtap Savas ^2^, Kimberley Thibodeau ^3^ and Carmen G. Loiselle ^4^

1. Department of Experimental Medicine, Faculty of Medicine and Health Sciences, McGill University, Montreal, Canada
2. Division of Biomedical Sciences, Faculty of Medicine, Memorial University, St John’s, Canada
3. McGill University Health Centre, Montreal, Canada
4. Department of Oncology and Ingram School of Nursing, Faculty of Medicine and Health Sciences, McGill University, Montreal, Canada

Background/Rationale or Objectives/Purpose

Ongoing collaborations, clear priorities, and new partnerships among cancer care organizations are key in informing an inclusive and timely psychosocial oncology (PSO) advocacy agenda. The CAPO Advocacy Committee continues its efforts to (1) develop a large repository of Canadian organizations advocating for PSO, and (2) co-create a national advocacy agenda that puts forward key priorities and action items.

Methodology or Methods

Medline Ovid, social media, grey literature, and informal feedback served to identify organizations with a focus on advocating for PSO. Status (e.g., NGO, charitable) and main advocacy activities were identified for each organization. The development of a larger survey is underway to capture further organizational characteristics (e.g., advocacy subcommittees, advocacy budget, number of staff/volunteers involved), outreach (i.e., local, provincial, national), key priorities, platforms, as well as current successes and challenges.

Impact on Practice or Results

Fifty-two organizations confirmed their psychosocial oncology advocacy commitment. Among these, 71% (*n* = 37) reported being non-profit and 54% (*n* = 28) as a charity (*n* = 19) reporting being both non-profit and a charity). The two main PSO advocacy activities selected by the organizations were raising awareness on cancer-supportive needs (90%; *n* = 47) and promoting initiatives that support person-centered psychosocial care (88%; *n* = 46).

Discussion or Conclusions

As we enter a renewed focus on innovation, collaboration, and co-design in cancer care, joining efforts with cancer advocacy organizations across the nation will ensure better support for individuals affected by cancer.

73

### 3.7. Development of an Inclusive Cancer Care Program for Sexual- and Gender Diverse Patients with Cancer: The Experience at the Princess Margaret Cancer Centre in Toronto

Christian Schulz-Quach ^1,2^, Brendan Lyver ^2^, Gilla Shapiro ^2,3^, Jennifer Croke ^4,5^, Marie-Anne Archambault-Grenier ^6,7^ and Margo Kennedy ^2^

1. Department of Psychiatry, Temerty Faculty of Medicine, University of Toronto, Toronto, Canada
2. Department of Supportive Care, Princess Margaret Cancer Centre, University Health Network, Toronto, Canada
3. Department of Psychiatry, University of Toronto, Toronto, Canada
4. Radiation Medicine Program, Princess Margaret Cancer Centre, University Health Network, Toronto, Canada
5. Department of Radiation Oncology, University of Toronto, Toronto, Canada
6. Centre Intégré de Santé et des Services Sociaux de Sud de Lanaudière, Montreal, Canada
7. Association des Médecins Hématologues et Oncologues du Québec, Canada

Background/Rationale or Objectives/Purpose

Patients, caregivers and chosen family who identify as Two-Spirit, lesbian, gay, bisexual, transgender, queer, intersex and asexual (2SLGBTQIA+) experience substantial challenges in cancer care, from screening, to cancer treatments, and in survivorship and end-of-life care. The Princess Margaret (PM) Cancer Centre in Toronto, Ontario, is developing an innovative, inclusive program to provide specialised support to 2SLGBTQIA+ patients and their caregivers. Our program uses the term Sexual and Gender Diversity (SGD) to encompass intersectional identities and experiences.

Objectives

1. To describe the process, and 10 core components that were established in the development of our SGD cancer care (SGDc) program;
2. To understand what inclusive care means to SGDc patients and their caregivers as well as their preferences for the SGD specialised program;
3. To identify challenges and lessons learned in our program development process.

Methodology or Methods

We utilized a multilevel Quality Improvement (QI) framework approach, including an organizational (CDC program evaluation framework) as well as a clinical lens (Plan-Do-Study-Act method), to develop an innovative, inclusive care program model for SGDc patients and caregivers.

Impact on Practice or Results

The program aims to improve the identification, support, and experience of SGDc patients/caregivers along the cancer care continuum. Impact on organizational identity, environmental signifiers and clinical service development will be presented.

Discussion or Conclusions

Lessons learned in our program development process will be addressed along with a discussion of future program directions. We will also provide an overview of our pilot project in Quebec.

75

### 3.8. Needs Assessment for an Inclusive Care Cancer Program for Sexual- and Gender Diverse Cancer Patients: A Quality Improvement Project at The Princess Margaret Cancer Centre

Margo Kennedy ^1^, Lauren Squires ^1,2^, Brendan Lyver ^1^, Shawn Hercules ^1^, Iowyth Hezel Ulthiin ^1^, Paige Lau ^1^, Gilla Shapiro ^1,3^, Jennifer Croke ^1^, Jackie Bender ^1,2^ and Christian Schulz-Quach ^1,4^

1. University Health Network, Toronto, Canada
2. Dalla Lana School of Public Health, University of Toronto, Toronto, Canada
3. Department of Psychiatry, University of Toronto, Toronto, Canada
4. Temerty Faculty of Medicine, University of Toronto, Toronto, Canada

Background/Rationale or Objectives/Purpose

Patients, caregivers and chosen family who identify as Two-Spirit, lesbian, gay, bisexual, transgender, queer, intersex and asexual (2SLGBTQIA+) experience substantial challenges receiving inclusive cancer care. The Princess Margaret (PM) Cancer Centre is developing an innovative inclusive cancer program to provide specialized support to Sexual and Gender Diversity in cancer care (SGDc) patients, their caregivers and their chosen families. A needs assessment was conducted with SGDc patients and caregivers to determine what is needed to provide an inclusive cancer care experience at our centre.

Objectives

1. To identify patients and caregivers’ top priorities for an inclusive sexual and gender diverse cancer care program;
2. To understand challenges and lessons learned from the clinical care experiences of patients and caregivers at PM.

Methodology or Methods

We conducted a quality improvement project incorporating the PDSA method as well as the CDC program evaluation framework. Five semi-structured qualitative interviews were completed and analyzed, and an additional 25 are planned (Feb–April 2023). Recruitment via posters, social media, and healthcare providers is ongoing. Interviews will be transcribed, verified, and analyzed using thematic analysis within MAXQDA

Impact on Practice or Results

Preliminary interviews provided good support for the interview guide, confirmed the need for dedicated clinical service development and provided directions for prioritizing psychosocial support, dedicated support groups, and resource navigation. Findings will be presented at the conference.

Discussion or Conclusions

Our pilot data confirm the current literature and prioritizes navigation, psychosocial support and dedicated group support offerings for SGDc patients and their chosen family.

92

### 3.9. Equity, Diversity, and Inclusion across Exercise Oncology: Exploring Exercise Preferences, Barriers, and Facilitators for LGBTQIA2S+ Individuals Living with and beyond Cancer

Benny Rana ^1^, Mannat Bansal ^1^, Helen MacRae ^2^, Lin Yang ^3,4^, William Bridel ^1^ and S. Nicole Culos-Reed ^1,5^

1. Faculty of Kinesiology, University of Calgary, Calgary, Canada
2. Participant Advisor, Calgary, Canada
3. Cancer Epidemiology and Prevention Research, Alberta Health Services, Calgary, Canada
4. Department of Community Health Sciences, Cumming School of Medicine, University of Calgary, Calgary, Canada
5. Department of Oncology, Cumming School of Medicine, University of Calgary, Calgary, Canada

Background/Rationale or Objectives/Purpose

To explore preferences, barriers, and facilitators to exercise participation in LGBTQIA2S+ individuals living with and beyond cancer.

Methodology or Methods

A qualitative study, using semi-structured interviews, explored exercise preferences, barriers, and facilitators for exercise programming from the experience of LGBTQIA2S+ individuals living with and beyond cancer. A participant advisor provided expertise throughout the study. Grounded in interpretive description methodology, the data gathered from the semi-structured interviews will be analyzed using thematic description.

Impact on Practice or Results

In ongoing work to date, *n* = 10 participants were recruited through outreach to cancer and LGBTQIA2S+ support groups and community organizations such as Wellspring, Calgary Outlink, Centre for Sexuality, and through social media. Interviews were conducted in person and online. Preferences, barriers, and facilitators are being identified for LGBTQIA2S+ individuals living with and beyond cancer.

Discussion or Conclusions

This study addresses a critical gap, supporting equity, diversity, and inclusivity for the LGBTQIA2S+ individuals within our understanding of the role of exercise in oncology. The results gained from this study may facilitate the tailoring of exercise (education and programming) as a supportive cancer care resource for LGBTQIA2S+ individuals living with and beyond cancer.

111

### 3.10. Exploring Responsive and Supportive Cancer Care with Racialized Adolescents and Young Adults

Tiffany Hill ^1^, Param Gill ^1^, Ada Okonkwo ^1^, Cheryl Heykoop ^1^, Jennifer Wolfe ^1^, Leah Lambert ^2^, Helen McTaggart-Cowan ^3^, Karine Chalifour ^4^ and Sarah Fletcher ^5^

1. Royal Roads University, Victoria, Canada
2. BC Cancer, Vancouver, Canada
3. BC Cancer Research, Vancouver, Canada
4. Young Adult Cancer Canada, St. John’s, Canada
5. UBC, Victoria, Canada

Background/Rationale or Objectives/Purpose

Annually, nearly 8000 adolescents and young adults, aged 15–39, are diagnosed with cancer in Canada. Currently, cancer care systems have limited capacity to meet the complex needs of young adults, and this is particularly true for racialized young adults.

Through a CIHR grant, the Anew Research Collaborative is actively collaborating with racialized young adults with lived experience of cancer and cancer care allies (clinicians, decision makers, care supporters, and researchers), to explore how cancer care systems can better respond to the specific needs and priorities and racialized young adults.

Methodology or Methods

This 18-month patient-oriented, participatory action research study has four phases. The first phase completed in April 2023 engaged approximately 20 racialized young adults in semi-structured interviews to better understand lived experiences, needs and priorities. The second phase will engage racialized young adults in a participatory and creative process to further explore the findings emerging from the interviews, the third phase will share results with clinicians and explore the support they require to better respond to the needs of racialized young adults, and the fourth phase will bring racialized young adults and cancer care allies together to engage in dialogue and identify creative and innovative strategies to facilitate more responsive and supportive cancer care for racialized young adults.

Impact on Practice or Results

Our presentation will share research findings and results emerging from the interviews and subsequent research methods.

Discussion or Conclusions

We will explore study learnings with a focus on ethics, equity, and partnerships, and will consider how this research is supporting Anew to be more equitable within our work.

117

### 3.11. Underserved Communities Report: Advancing Health Equity through Cancer Information and Support Services

Laura Burnett, Apiramy Jeyapalan, Tracy Torchetti and Elizabeth Holme

The Canadian Cancer Society

Background/Rationale or Objectives/Purpose

The Canadian Cancer Society’s (CCS) underserved communities project identifies communities that face barriers to accessing cancer information and support. The project’s goal is to understand gaps and challenges faced by these communities and outline opportunities and tactics to address them. For our work, CCS has currently identified 10 underserved communities with 25+ sub-categories: adolescents and young adults with cancer, advanced cancer, Indigenous communities, LGBTQ2+, newcomers to Canada, non-English-speaking or non-French-speaking communities, older adults, racialized communities, rare cancers and rural and remote communities.

Methodology or Methods

A mixed-methods approach looking at both qualitative and quantitative data was used including literature reviews, stakeholder interviews and data and document reviews. Key informant interviews were conducted with CCS staff, patient advisory groups and organizations that engage with underserved communities, and cancer agencies nationally and internationally.

Impact on Practice or Results

In addition to identifying gaps, opportunities and recommended tactics, this project identified key considerations for engagement with each community and five overall recommendations to support the organization’s work with underserved communities: train staff, address capacity, engage with communities, co-design with communities and develop an evaluation framework. Diversity and intersectionality are considered as individuals are unique and may identify with more than one community, bringing those experiences with them.

Discussion or Conclusions

The evidence gathered through the underserved communities project will guide engagement efforts and strategies to better serve underserved communities with information, peer, psychosocial and practical supports in a meaningful, evidence-informed way. Further, CCS encourages other organizations to co-develop tailored initiatives with underserved communities, guided by the perspective of people with cancer from those communities.

123

### 3.12. Tailoring Exercise Oncology Resources for Punjabi-Speaking Individuals of South Asian Heritage Living with and beyond Cancer: A Participant-Oriented Approach

Mannat Bansal and Nicole Culos-Reed

University of Calgary, Calgary, Canada

Background/Rationale or Objectives/Purpose

Physical activity is an effective evidence-based supportive care intervention for individuals living with and beyond cancer. Despite individuals of South Asian heritage (Punjabi-speaking) making up the largest ethnic minority group in Canada, exercise oncology programs and educational resources are not available for this population. The objective of this study is to co-create tailored exercise oncology resources for Punjabi-speaking individuals of South Asian heritage.

Methodology or Methods

A participant-oriented approach will be taken to co-create tailored exercise oncology resources using the Knowledge to Action framework. Specifically, South Asian patients, families, as well as fitness professionals and healthcare providers who work in predominately South Asian communities in Calgary, will engage with the research team to build and translate exercise oncology educational and programming resources into Punjabi. Building resources that include visible representation of the target population will occur through an iterative process with the research team. Feedback on resources will be collected through surveys and focus groups interviews.

Impact on Practice or Results

This work is the first in a series of PhD studies aimed at enhancing accessibility for individuals of South Asian heritage to exercise oncology resources. Translated documents will include educational resources to support exercise behaviour change and training and delivery documents to support exercise oncology programs.

Discussion or Conclusions

The outcomes from this study will build equity in access to resources for individuals of South Asian heritage living with and beyond cancer. Ultimately, this work will support increasing the diversity of participants within exercise oncology programs, ensuring more individuals can benefit from exercise during the cancer journey.

## 4. Final Category: B. Cancer Care across the Life Span (Children, Adolescents and Young Adults, Adults, and Older Adults)

42

### 4.1. A Study-In-Progress: Understanding the Association between Physical Activity, Body Image and Quality of Life for Young Adult Males Living with and beyond Cancer

Tana Dhruva ^1^, Chad W. Wagoner ^1^, Sarah J. Kenny ^1^, David M. Langelier ^1,2^ and S. Nicole Culos-Reed ^1^

1. The University of Calgary, Calgary, Canada
2. University of Toronto, Toronto, Canada

Background/Rationale or Objectives/Purpose

Improvement of quality of life (QOL) for young adults (YA) (20–39 years) living with and beyond cancer is an important survivorship issue given the potential negative physical and psychosocial treatment-related outcomes that are experienced. This includes the impacts of treatment on body image (BI). Physical activity (PA) is an evidence-based tool that may positively impact both BI and QOL. There is a need to specifically examine the PA, BI, and QOL relationship in YA males, a relatively understudied population in the exercise oncology literature.

Methodology or Methods

Sample/Setting: Young adult males, ages 20–39 (*n* = 30). Research will be conducted at the Health and Wellness Lab in the University of Calgary.

Procedures: The proposed mixed-methods study will utilize semi-structured interviews and questionnaires to explore the association between PA, BI, and QOL. Potential participants will be recruited via outreach through pre-existing studies, support organizations, social media, and clinical settings. Self-reported measures will be used to collect data on BI (Body Image Scale), PA (Godin Leisure-Time Exercise Questionnaire) and QOL (Functional Assessment of Cancer Therapy).

Impact on Practice or Results

Descriptive statistics (mean, standard deviations) will summarize study sample characteristics including age, cancer type, etc. Pearson product moment correlations will examine associations among BI, PA, and QOL. The qualitative component (*n* = 10) will involve semi-structured interviews to facilitate a deeper understanding of these associations and will be analyzed using thematic analysis framed within an interpretive description approach.

Discussion or Conclusions

Knowledge from this study may be used to tailor PA interventions and educational resources for male YAs to address BI and QOL needs.

93

### 4.2. Reshaping Adolescents and Young Adults Cancer Care, Together

Cheryl Heykoop, Jennifer Wolfe, Tiffany Hill, Lily Rogers, Ada Okonkwo, Jon Avery and Param Gill

Royal Roads University, Victoria, Canada

Background/Rationale or Objectives/Purpose

In Canada, nearly 8000 adolescents and young adults (AYAs) are diagnosed each year with cancer, and their unique needs are largely unmet by cancer care systems. AYAs have highly specific medical and psychosocial needs. Simultaneously, cancer in AYAs often coincides with major life transitions such as: post-secondary education or employment, independent living, marriage and partnerships, parenthood or caring for aging relatives. It is well established that AYAs require life-stage appropriate cancer care distinct from children and older adults.

To develop AYA cancer care and support programming, it is critical that AYAs are actively engaged and involved. Research indicates young adults have a clear understanding of how their care can be improved and want to play an active role. Further, relevant patient engagement is a key principle in the Strategy for Patient-Oriented Research.

Methodology or Methods

The Anew Research Collaborative is working with AYAs and cancer care allies (supporters, clinicians, decision-makers, researchers) to develop an AYA program in BC through participatory, creative, and patient-oriented approaches.

Impact on Practice or Results

We have been engaging with AYAs to understand their lived experiences, needs, and priorities; working with clinicians to understand what they need to better support AYAs; and working with leadership within BC Cancer to create an AYA program.

Discussion or Conclusions

We continue to build from this momentum moving forward and are striving to be equitable and representative through our work, and also intend to co-develop materials, resources, and practices with AYAs moving forward to ensure the AYA program truly reflects AYAs’ needs and priorities.

99

### 4.3. Effectiveness-Implementation Hybrid Study Designs as a Method for Studying Supportive Care Interventions among Young People Diagnosed with Cancer

Emma McLaughlin ^1^, S. Nicole Culos-Reed ^1^, Anna Janzen ^2^, Hannah Cripps ^1^, Mannat Bansal ^1^, Julia Daun ^1^, Benny Rana ^1^, Tana Dhruva ^1^, Heather Molina ^3^, Georgia Kaluznick ^1^, Julianna Dreger ^1^ and Amanda Wurz ^2^

1. University of Calgary, Calgary, Canada
2. University of the Fraser Valley, Chilliwack, Canada
3. Bloom Wellness Collective, Calgary, Canada

Background/Rationale or Objectives/Purpose

Research suggests that physical activity (PA) is safe and beneficial for young people with cancer. However, there are few opportunities for this cohort to engage in population-specific PA and receive the potential benefits of PA. This may be due to challenges in moving knowledge to practice (i.e., knowledge translation). Effectiveness-implementation hybrid study designs can overcome some of these knowledge translation challenges. In this presentation, we will highlight our use of the effectiveness–implementation hybrid study design to move PA evidence to practice for young people with cancer.

Methodology or Methods

To illustrate how we are using the effectiveness–implementation hybrid study design, we will present an overview of two studies: (i) a 12-week group-based yoga for young adults with cancer, and (ii) a 12-week 1:1 PA intervention for children and adolescents with cancer.

Impact on Practice or Results

We will share how we have made important effectiveness–implementation study design and methodological decisions, including which knowledge translation and evaluation frameworks to utilize. We will describe how we are collecting and analyzing data and ensuring ongoing quality improvement of the intervention. To date, in study (i), 43 young adults have participated and 2 quality improvement cycles have occurred, and in study (ii), 5 children have participated and 1 quality improvement cycle has occurred.

Discussion or Conclusions

It is hoped that this presentation will provide insight into how effectiveness–implementation hybrid study designs can generate vital evidence to support knowledge translation to promote PA in the fields of exercise oncology and supportive cancer care.

101

### 4.4. Traumatic Stress Symptoms in Family Caregivers of Adults and Children with Newly Diagnosed Acute Leukemia: A Multisite Observational Study

Angela Mathews ^1,2^, Lindsay Jibb ^1,3^, Stephanie Nanos ^2,3^, Sarah Alexander ^1,3^, Carmine Malfitano ^1,2^, Anne Rydall ^2^, Argin Malakian ^1,2^, Kyle Fitzgibbon ^2^, Camilla Zimmermann ^1,2^ and Gary Rodin ^1,2^

1. University of Toronto, Toronto, Canada
2. Princess Margaret Cancer Centre, Toronto, Canada
3. The Hospital for Sick Children, Toronto, Canada

Background/Rationale or Objectives/Purpose

Acute leukemia (AL) is a hematologic malignancy of acute onset that represents a substantial threat to patients and their family caregivers (FCs), who may be partners, parents, or adult children. However, there is a gap in knowledge regarding the psychological effects of AL on FCs. This study aims to assess the prevalence, severity, and risk factors of traumatic stress (TS) symptoms in FCs following the diagnosis of AL.

Methodology or Methods

Adult FCs of patients of all ages with AL diagnosed within the preceding 3 months were recruited from two tertiary care centres in Toronto, Canada. Self-report questionnaires measured the severity of TS symptoms, depression, attachment security, caregiver burden, social support, satisfaction with medical care, and gender role at enrolment. Quantitative data were analyzed using descriptive and cross-sectional techniques, including multivariate analyses, to identify significant predictors of TS symptoms in FCs.

Impact on Practice or Results

A total of 54 FCs (spouses = 7, adult children = 2, parents = 45) were enrolled since February 2022. Clinically significant TS symptoms (SASRQ score ≥ 40) were reported by 37/54 (68.5%) FCs. In the total sample, TS symptoms were positively associated with attachment anxiety (r = 0.601, *p* < 0.001) and depression (r = 0.754, *p* < 0.001), and negatively associated with social support (r = −0.365, *p* = 0.007) and satisfaction with medical care (r = −0.378, *p* = 0.005).

Discussion or Conclusions

FCs of adult and pediatric patients with AL are at high risk of TS symptoms, which are associated with low mood, attachment insecurity, limited social support, and dissatisfaction with medical care. Although further research is needed to establish causality, these findings may inform the development of preventive and therapeutic interventions for this understudied population.

106

### 4.5. Predictors of Parent and Child Health-Related Quality of Life among Infants and Toddlers with Cancer

Sara Cho ^1^, Hailey Zwicker ^1^, Sharon Hou ^1,2^, Iqra Rahamatullah ^1^, Tak Fung ^1^, Nicole Racine ^3,4^, Janelle Morhun ^1^, Gregory Guilcher ^1^ and Fiona Schulte ^1^

1. University of Calgary, Calgary, Canada
2. British Columbia Children’s Hospital, Vancouver, Canada
3. University of Ottawa, Ottawa, Canada
4. Children’s Hospital of Eastern Ontario, Ottawa, Canada

Background/Rationale or Objectives/Purpose

This study aimed to describe the health-related quality of life (HRQOL) of infants and toddlers diagnosed with cancer and their caregivers over time and to identify variables predictive of HRQOL for infants and their parents.

Methodology or Methods

HRQOL was assessed for infants and toddlers (aged <4 years) on active treatment for cancer, and their parents (physical and mental HRQOL) at three time points (baseline, 6 months, 12 months). Linear mixed models were used to investigate whether demographic and treatment variables, parental HRQOL, and parental distress predicted child HRQOL over time.

Impact on Practice or Results

Thirty-nine infants and toddlers (51.20% male, 29.96 ± 15.32 mean age in months) and parents participated. At baseline, 51% of parents reported poor mental HRQOL. Child HRQOL was significantly lower than population norms but showed no difference compared to an older pediatric cancer sample. No significant variables were found to predict parent HRQOL, including child HRQOL, time since diagnosis, income, and intensity of treatment (*p* > 0.05). However, parent distress and child HRQOL bidirectionally predicted each other, whereby lower parental distress was associated with higher child HRQOL (B = −2.1, se = 0.078, *t* = −2.70, *p* = 0.009), and vice versa (B = −0.50, se = 0.119, t = −2.67, *p* = 0.010).

Discussion or Conclusions

Both infants and toddlers with cancer and their parents experience poorer HRQOL. Interventions targeting the parent’s distress, emphasizing the importance of the parent–child relationship, and the importance of self-care may be extremely important in improving the HRQOL of parents and children. Clinically, healthcare providers should routinely screen parents to assess their well-being and distress and provide appropriate support when needed.

112

### 4.6. Transforming Adolescent and Young Adult Cancer Care through Creativity and Theatre

Cheryl Heykoop ^1^, Will Weigler ^2^, Genevieve Stonebridge ^2^, Alice O’Grady ^3^, Jennifer Wolfe ^1^, Lily Rogers ^1^ and Fiona Thatcher ^4^

1. Royal Roads University, Victoria, Canada
2. N/A, Victoria, Canada
3. Leeds University, Leeds, UK
4. InspireHealth, Vancouver, Canada

Background/Rationale or Objectives/Purpose

Each year in Canada, nearly 8000 adolescents and young adults (AYAs; people aged between 15 and 39) are diagnosed with cancer. Cancer in AYAs often coincides with major life transitions (e.g., post-secondary education or employment, independent living, marriage and partnerships, parenthood) and the new onset of serious illness at this life-stage presents unique medical and psychosocial challenges. It is well established that AYAs require life-stage appropriate cancer care distinct from children and older adults, yet the unique needs of AYAs remain largely unmet by cancer care systems. Further, AYAs have a clear understanding of how to improve care and want to play a more active role in doing so.

Methodology or Methods

Weaving together three distinct yet complementary methodologies: Patient Activated Research, Participatory Action Research, and Performance As Research, we engaged young adults as co-researchers to create both videos and art to share unique aspects of AYA cancer and identify areas to improve cancer care for AYAs.

Impact on Practice or Results

A total of 15 AYAs participated in a six-week series to create nine short videos (forthcoming in April 2023) and 25 AYAs participated in a five-week creative art series (April to end of May 2023). In this presentation, we share some of the creative outputs developed and discuss key findings emerging.

Discussion or Conclusions

The findings of this research will be shared with cancer care allies (clinicians, supporters, decision-makers, and researchers) and AYAs to engage in dialogues about how to improve cancer care for AYAs in BC and beyond.

126

### 4.7. Optimizing the Experience and Use of Oral Chemotherapy at Home in Pediatric Acute Lymphoblastic Leukemia: A Collaborative Approach to Develop an Intervention Program for Families

Isabelle Laverdière ^1,2,3^, Sophie Lauzier ^1,4^, Angéline Labbé ^1,2^, Marianne Olivier D’Avignon ^5^, Laurence Guillaumie ^2,6^ and Pierre-Marie David ^7^

1. Faculty of pharmacy, Université Laval, Québec, Canada
2. CHU de Québec-UL Research Center, Québec, Canada
3. Department of Pharmacy, CHU de Québec, Québec, Canada
4. CHU de QuébecUL Research Center, Québec, Canada
5. Faculty of social sciences, Université Laval, Québec, Canada
6. Faculty of nursing, Université Laval, Québec, Canada
7. Faculty of pharmacy, Université de Montréal, Montreal, Canada

Background/Rationale or Objectives/Purpose

Pediatric acute lymphoblastic leukemia (ALL) treatment includes two years of home-based oral chemotherapies (OC) to prevent relapses. OC-related challenges are multifaceted and can affect medication intake and the well-being of the entire family. We aim to design a program to support families with OC.

Methodology or Methods

The program was designed using the Behaviour Change Wheel framework comprising three steps: (1) Understanding the behaviour; (2) Identifying intervention options; (3) Identifying content and implementation options. We used a participatory approach by involving a committee of parents of a child with ALL (*n* = 4) and health and psychosocial professionals (*n* = 5) at each stage.

Impact on Practice or Results

(1) A qualitative study conducted among 13 parents of a child with ALL comprehensively depicted OC challenges. The program aims to: “In collaboration with the healthcare team, families address the OC difficulties by mobilizing tools and resources to support OC use and family well-being”. It addresses (a) efficient and secure use of OC; (b) coping with their physical, psychological and social impacts; (2) The intervention functions are education, training, enablement, persuasion, modeling and environmental restructuring; (3) Three modules are provided at key points from hospital discharge and over the 2-year treatment: (a) education and training provided by the multidisciplinary team and supported by explanatory sheets and videos; (b) systematic evaluation of OC-related difficulties; (c) tools for their self-management and normalizing family experience.

Discussion or Conclusions

The program’s material is under development and will be pilot-tested in a mixed-methods feasibility study. The combination of systematic and participatory approaches for program development may increase its efficacy and implementation.

## 5. Final Category: C. Complementary and Integrative Cancer Care

4

### 5.1. Complementary and Alternative Medicine Use among Cervical Cancer Patients

Ifeyinwa Onwukeme ^1^, Dr. Kassim Davidson ^1,2^ and Kelechi Nneke ^1^

1. Sali Hoe Foundation, Abuja, Federal Capital Territory, Nigeria
2. Federal Medical Centre, Lokoja, Nigeria

Background/Rationale or Objectives/Purpose

The objective of the study was to ascertain the prevalence of complementary and alternative medicine (CAM) use among cervical cancer patients.

Methodology or Methods

This was a cross-sectional study conducted among 420 cervical cancer patients randomly selected using systematic sampling technique. The instrument of data collection was a semi-descriptive structured questionnaire. The mean age of the study participants was 45 ± 13.7 years. The predominant source of information on complementary and alternative medicine (CAM) use was from family and friends, 50.5%.

Impact on Practice or Results

The prevalence of CAM use in the study was 66.3% and the frequent CAMs used in the study were naturally based method (herbs 28.3%) and mind/body interventions (prayer 30.8%). Sex, occupation and concurrent illness were some of the factors associated with CAM use.

Discussion or Conclusions

Often times, the decisions on how to manage cancer using CAM is due to the hopeless situation in which most cancer patients found themselves. The thought of using complementary and alternative medicine with reliable information and support from their family might be considered especially where conventional methods failed to provide solutions.

It is imperative that health professionals explore the use of CAM with cancer patients and educate them on the potential benefits.

95

### 5.2. A Complex Adaptive Systems Perspective—Client Feedback of the Canadian Cancer Society’s Cancer Information Helpline Provides Insights of Cancer Needs and Program Expectations at Clinical, Practical and System Levels

Holly Bradley ^1^, Sharon Lee ^2^, Peter Nugus ^3^, Raissa Passes dos Santos ^3^, Julia Deeks ^1^ and Laura Burnett ^2^

1. Canadian Cancer Society, Toronto, Canada
2. Canadian Cancer Society, Hamilton, Canada
3. McGill University, Montreal, Canada

Background/Rationale or Objectives/Purpose

The Canadian Cancer Society’s Cancer Information Helpline (CIH) provides information (via phone and digital modes) to people with cancer and their caregivers at all stages of the continuum from prevention onward. Going beyond the traditional helpline literature that focuses on client demographics and topics of discussion, this study used client feedback to identify ways to improve the quality of the CIH and inform understanding of components of client satisfaction within the complexities of service provision.

Methodology or Methods

A qualitative thematic analysis was completed on 3,390 client responses to two open-ended survey questions about the help provided by the CIH and recommendations for program change. In addition to key themes, a broader systems perspective was applied.

Impact on Practice or Results

Thematic analyses revealed that the CIH responds to multi-level system needs. Further, it was difficult for some clients to separate the CIH from other organizational or health system services because this was their point-of-entry. To meet client expectations, responsiveness, practical service provision, guidance through information and psychosocial support, empathetic demeanour, and empowerment and advocacy, were the most important factors influencing client satisfaction.

Discussion or Conclusions

To meet client expectations, operations and staff training that produce timely, supportive and empathetic interactions are important. CIHs must be prepared to respond not only to informational, emotional, practical and physical needs, but also navigate needs in a broader organizational and system context that includes other services not delivered directly by the helpline, and institutions beyond.

114

### 5.3. The Virtual Mind Study: Virtual Reality Guided Mindfulness (VRGM) for Chronic Cancer Related Pain

Zen Gajtani ^1^, Mohamad Baydoun ^2^, Haley Mather ^1^, Kathryn Birnie ^1^ and Linda Carlson ^1^

1. University of Calgary, Calgary, Canada
2. University of Regina, Regina, Canada

Background/Rationale or Objectives/Purpose

Chronic cancer-related pain (CRP) adversely impacts cancer survivors’ quality of life. Virtual reality (VR) may be an effective medium for delivering mindfulness, which has been shown to reduce chronic pain but remains unexamined in cancer survivors. We investigated the feasibility of a VR-guided mindfulness (VRGM) intervention for adult cancer survivors with chronic CRP.

Methodology or Methods

This mixed-methods feasibility study uses a single-arm, pretest–post-test design with semi-structured interviews conducted post-intervention. Cancer survivors participate in the 6-week, home-based intervention consisting of 10–15 min daily VRGM practice. Feasibility and psychosocial outcomes (i.e., pain, sleep, depressive and anxiety symptoms, fatigue, quality of life, mindfulness) are assessed at three time points.

Impact on Practice or Results

Participants (*N*= 20) mean age was 49.8 years, 90% were female, and diagnoses included breast (60%), hematologic (15%), central nervous system (15%), gynecologic (5%), and thyroid (5%) cancers. Survivors reported moderate to severe pain severity lasting for 3 months to 6 years post-treatment. A total of 15 participants successfully completed the 6-week program resulting in a 75% retention rate. Preliminary qualitative analysis shows participants report beneficial effects of VGRM for pain management, anxiety, sleep and fatigue. Analyses are in progress and final qualitative and quantitative results will be presented.

Discussion or Conclusions

Survivors of cancer experience increased levels of psychosocial symptoms and pain interference. This novel intervention shows high participant acceptability and retention and may provide a potential alternative treatment to opioid analgesics. Results from the present study can inform future larger VGRM trials for chronic CRP to help reduce suffering in people with cancer.

## 6. Final Category: D. Community-Based and Volunteer Cancer Care Services

7

### 6.1. Effective Community-Based Cancer Care

Debbie Magwood

West Island Cancer Wellness Centre, Kirkland, Canada

Background/Rationale or Objectives/Purpose

Research points to the necessity of a whole-person approach to cancer care (Rubin et al., (2015), Polley et al., (2016), and Nixon et al., (2021)). The medical systems are overextended. Focusing on combatting this pesty disease, they must be hyper-focused on the disease model, leaving very little room, both literally and financially, to be able to respond these research findings. The solution lies in services giving by the West Island Cancer Wellness Centre (WICWC) (and like centres).

At the WICWC, professional volunteers providing services that they are already knowledgeable, well-skilled and comfortable providing. This removes barriers such as fears of the unknown/lack of self-efficacy/lack of skills or experience, all components that research suggests have been barriers for volunteers (Ormord (2006) and Haski-Leventhal et al., (2009) as cited by Lockstone Binney et al., (2021)).

Methodology or Methods

Professionals apply to volunteer and are interviewed, reference checked and vetted. A police verification is performed and the volunteer is given an orientation prior to being offered a position. Performance evaluations of the volunteer are conducted randomly, as well.

Impact on Practice or Results

The WICWC, registered as a charity in 2008, supports empirical research and has a VERY unique model of administration of care. Our model utilizes professional volunteers, thus operating within a small budget.

The WICWC offers upwards of 3500 individual hours of programs/services, given by volunteers. If we were to pay for these professionals, our operational budget would exceed CAD 3 million.

Discussion or Conclusions

How we save millions, cultivate relationships, and how we use their services is the heart of this presentation.

8

### 6.2. The Promise of Longitudinal Program Evaluation to Inform Community-Based Cancer Supportive Initiatives

Samar Attieh ^1,2^, Suzanne O’Brien ^2,3^ and Carmen G. Loiselle ^3,4^

1. Department of Experimental Medicine, Faculty of Medicine and Health Sciences, McGill University, Montreal, Canada
2. Hope & Cope, Jewish General Hospital, Montreal, Canada
3. Segal Cancer Center, Jewish General Hospital, Montreal, Canada
4. Department of Oncology and Ingram School of Nursing, Faculty of Medicine and Health Sciences, McGill University, Montreal, Canada

Background/Rationale or Objectives/Purpose

Community-based cancer organizations, such as Hope & Cope in Montreal, Qc, are increasingly relying on evidence to inform their cancer supportive initiatives. Gathering user perceptions of these initiatives such as expectations, design, content, and modes of delivery is key. The main objectives of this longitudinal evaluation program are to (1) explore and compare user perceptions of Hope & Cope initiatives, prior to program exposure, during attendance, and at program completion, and (2) document participants’ self-reported hopefulness and coping pre- and post-program participation.

Methodology or Methods

Methods: Using a mixed-method design, five supportive programs will be evaluated. A total of *n =* 75 individuals impacted by cancer and planning to attend a Hope & Cope initiative will be recruited (*n* = 15 for each of the five programs to be evaluated). Participants will be asked to complete self-report online questionnaires pre- and post-program attendance including Hope (Herth, 1992) and Cope (Carver,1997) measures. They will also be asked to participate in semi-structured interviews (pre-, mid-, and post-program).

Impact on Practice or Results

In addition to important qualitative insights gained over time, we hypothesize that, at program completion, hopefulness and coping will be significantly higher.

Discussion or Conclusions

Findings will provide key insights into optimal design, implementation, and contributions of these community-based cancer supportive programs so that they continue to be person-centered, co-designed, and situation responsive.

57

### 6.3. Expansion of Community-Based Psychosocial Oncology: A Pilot Project in Rural Nova Scotia

Katrin Julia Kaal ^1,2^, Marianne Arab ^3^, Pamela Robichaud ^3^, Joy Tarasuk ^3^, Maggie Arenburg ^2^, Janice Howes ^3^, Matthew Stanwood ^2^, Kara Jamieson ^2^, Margaret Ann Morrison ^3^ and Helmut Hollenhorst ^3^

1. Dalhousie University, Halifax, Canada
2. Nova Scotia Health, Halifax, Canada
3. Cancer Care Program, Nova Scotia Health, Halifax, Canada

Background/Rationale or Objectives/Purpose

Yarmouth, NS and surrounding counties have the highest travel burden (>2.5 h) to access cancer care. To mitigate the existing risk to patients who are either not accessing care, experience delays to care, or face an additional burden of extended travel and costs during care, a bundle of equity-enhancing investments to the local community cancer center included additional psychosocial oncology resources; specifically, a full-time dedicated Social Worker (SW) and part-time Drug Access Navigator (DAN) joined a well-established Cancer Patient Navigator (CPN).

Methodology or Methods

The implementation of the new positions into the care team was informed by (1) key informant interviews of each cancer care team member, (2) a focus group with the three PSO HCPs, and (3) sustained team engagement sessions. The goal and outcomes of these implementation strategies were: clarification of roles, scopes of practice, referral guidelines and the optimization as well as re-distribution of PSO duties and responsibilities that had previously been carried out by the CPN and other, non-PSO, team members.

Impact on Practice or Results

The expansion and careful integration of additional PSO resources at the pilot site is associated with more equitable access to PSO care, better outcomes for patients, better patient and HCP experience, and increased efficiency of PSO care delivery (i.e., quintuple aim).

Discussion or Conclusions

Dedicated efforts facilitated the successful integration of additional PSO resources at a rural community oncology site. This model of PSO care served as catalyst for the province and is currently being expanded to seven additional community oncology sites in NS.

59

### 6.4. Peer Support Needs and Digital Peer Navigation Preferences among Head and Neck Cancer Patients

Logan Meyers ^1^, Katie Kennedy ^1,2^, Maria Fusaro ^2^, Melanie Woodside ^1^, Jolie Ringash ^1,2^ and Jackie Bender ^1,2^

1. Princess Margaret Cancer Centre, Toronto, Canada
2. University of Toronto, Toronto, Canada

Background/Rationale or Objectives/Purpose

Head and neck cancer (HNC) patients experience unique barriers to optimal supportive care and lack access to peer support. We investigated the need for, and barriers to, peer support, and interest in digital peer navigation among HNC patients.

Methodology or Methods

A cross-sectional survey was administered to HNC patients in clinic and by email. HNC patients who were ≥ 18 years of age, English-speaking, and receiving care at the Princess Margaret Cancer Centre were invited to participate. Descriptive statistics were calculated, and multivariable analyses examined factors associated with desire for peer navigation and being a peer navigator.

Impact on Practice or Results

Participants (*n* = 170) were on average 59 years-of-age (SD = 13.1), 3.17 years (SD = 3.4) post-diagnosis and 73.4% were men. A total of 50% were interested in peer support, but 62% had not accessed peer support. The top barriers to peer support were: finding HNC-specific in-person programs (76.9%), finding HNC patients with whom they could relate (73.1%), and the inconvenience of in-person programs (71.1%). Nearly half (47%) desired support from a peer navigator through a digital app, and 43% were interested in being a peer navigator. Top preferred peer-matching characteristics were type of cancer (80.6%), treatment received (74.8%), and specific concerns (74.4%). Participants who were distressed (*p* = 0.03) were more likely to desire peer navigation. Having had chemotherapy (0.04) and good social support (*p* = 0.04) were associated with the desire to be a peer navigator.

Discussion or Conclusions

A digital peer navigation program was desired by almost half of HNC patients and could overcome some barriers to peer support, particularly for those who are distressed.

94

### 6.5. Qualitative Study of Coping with Hematologic Cancer during the Pandemic among Underserved Patients Engaged in a Cancer Wellness Program: “I Saw the Unity…as if [We] Were Hand to Hand”

Brittany Miller, Johnna Bakalar and Alyson Moadel-Robblee

Montefiore Einstein Cancer Center, Bronx, USA

Background/Rationale or Objectives/Purpose

Hematologic cancer (HC) is associated with significant challenges to quality of life due to multi-modal and systemic treatments. This study aimed to understand how underserved HC patients in Bronx, NY cope with the stress of cancer and engage with a free psychosocial and peer support program, called BOLD, during the COVID-19 pandemic.

Methodology or Methods

Twenty-nine HC patients between the ages of 19 and 76 (38% under age 50) were identified through convenience sampling through our Bronx cancer center and surveyed by phone. Patients were African American (52%), Hispanic/Latino (52%), and primarily female (62%). The study was conducted by the BOLD Program team. During the needs assessment survey, HC patients were asked open-ended questions on: (a) how they cope with cancer, (b) if/how BOLD has been helpful, and (c) barriers to participation.

Impact on Practice or Results

Thematic analysis of qualitative responses revealed that social support was the most common strategy for coping with cancer (41%). Social support, with an emphasis on shared experience and calls from staff, was what patients found to be most helpful from BOLD (55%). Women exclusively endorsed emotional support as a benefit, whereas men endorsed education. The top barrier to participation was competing demands (24%).

Discussion or Conclusions

Social support within a peer and common purpose community is a critical element of psychosocial wellness, particularly during the pandemic. Providing cancer education may engage more men, who often underutilize psychosocial services. Including evening programming may be helpful in engaging patients limited by other responsibilities, such as work, school, childcare, and appointments.

## 7. Final Category: E. Sociodemographic, Culture, and Sex/Gender Issues in Cancer

19

### 7.1. Cancer Care Experiences by Intersecting Identities in the Adolescent and Young Adult Population: A Scoping Review Protocol

Michaela A. Bourque ^1^, Julie Deleemans ^2^ and Alyson L. Mahar ^1^, on behalf of the MEGAN-CAN team

1. Queen’s University, Kingston, Canada
2. University of Calgary, Calgary, Canada

Background/Rationale or Objectives/Purpose

Adolescents and young adults (AYAs) may be at a higher risk of poor cancer outcomes because of health disparities exacerbated by their unique intersecting identities. The scoping review aims to identify gaps in methodologies and the application of intersectional approaches within AYA-focused research across the cancer continuum.

Methodology or Methods

Sample and Setting: The search includes work published between 1 January 2010 and 31 December 2022. Electronic databases, including MEDLINE, EMBASE, Cumulative Index to Nursing and Allied Health Literature, PubMed, and PsycINFO, will be systematically searched.

Procedures: This review follows a scoping methodology based on the Arksey and O’Malley expanded scoping review framework and the Preferred Reporting Items for Systematic Reviews and Meta-Analyses (PRISMA) extension for reporting scoping reviews: the PRISMA-Scoping Reviews checklist. A search strategy developed in conjunction with a health sciences librarian will be applied. Search terms covering the broad categories of cancer, AYA, and intersectionality (i.e., age, sex, gender, race, socioeconomic status, and disability) will be applied. Two authors will screen titles, abstracts, and full texts independently to determine inclusion. Data from eligible full-text publications will be extracted and summarized both quantitatively and qualitatively.

Impact on Practice or Results

Analysis is in progress. Preliminary findings will be presented.

Discussion or Conclusions

This scoping review will provide a critical overview of intersectionality alongside a methodological overview of work undertaken in the AYA cancer space. It is vital that AYAs’ intersecting identities be examined in the literature, as disparities must be critically examined if patient-centred, developmentally appropriate, personalized cancer care is to be provided.

85

### 7.2. This Is Not Your Mother’s Menopause: A Qualitative Exploration of BRCA1/2 Carriers Psychosocial Outcomes Post-Oophorectomy

Laurice Karkaby ^1,2^ and Gillian Einstein ^1^

1. University of Toronto, Toronto, Canada
2. Princess Margaret Cancer Centre, Toronto, Canada

Background/Rationale or Objectives/Purpose

Women who carry a BRCA (BReast CAncer) 1 or 2 mutation are counselled to have a risk-reducing bilateral salpingo oophorectomy (removal of both ovaries and fallopian tubes (BSO)), prior to age 40, or when childbearing is complete. While cancer-related worry markedly decreases following BSO, women report experiencing a host of physical and psychosocial outcomes. The rationale of this research study was to gain first-person perspectives about the psychosocial outcomes post-BSO.

Methodology or Methods

Semi-structured, open-ended interviews of 1–2 h were conducted via Zoom or telephone with 14 BRCA carriers post-BSO. Interview transcripts were analyzed using thematic analysis.

Impact on Practice or Results

A key theme arising was that women with BRCA-BSO felt much older than their chronological age. This was engendered by both their physical symptoms post-BSO and the cultural baggage accompanying the label of their condition as “menopause”. Several women rejected the negative imagery of this label—its association with aging as well as the normalizing of their condition at their young age. While women taking estrogen replacement therapy viewed it as their “little ovaries”, perhaps staving off some negative physical side effects, the women shared their frustrations of not being immune to the negative stereotypes and stigma that surround menopause.

Discussion or Conclusions

These interviews provide insight into the limited information women receive about the psychosocial outcomes of BSO. These accounts support that healthcare providers should be sensitive to the notion that induced menopause is a unique experience onto itself.

86

### 7.3. Loneliness in Pediatric Brain Tumour Survivors

Araby Roberts ^1,2^, Mehak Stokoe ^1^, Taryn Fay-McClymont ^1^, Gregory Guilcher ^1^, Lucie Lafay-Cousin ^1^, Keith Yeates ^1^, Kevin Krull ^3^ and Fiona Schulte ^1,2^

1. University of Calgary, Calgary, Canada
2. Alberta Children’s Hospital, Calgary, Canada
3. St. Jude Children’s Research Hospital, Memphis, USA

Background/Rationale or Objectives/Purpose

Pediatric brain tumour survivors (PBTS) have poor social adjustment, including loneliness, but it is unclear whether adjustment is associated with sociodemographic factors. Previous findings suggest social adjustment is poorer for female, racial/ethnic minority, older current age, or lower household income pediatric cancer survivors. We examined associations between sociodemographic factors and loneliness for PBTS.

Methodology or Methods

Thirty-three PBTS (*M*_age_ = 14.0 years [*SD* = 3.3], female = 54.6%, white = 81.8%) were recruited from a local long-term survivor clinic. Thirty-five healthy controls (*M*_age_ = 11.6 years [*SD* = 3.0], female = 51.4%, white = 71.4%) were recruited from the community.

Participants completed the Children’s Loneliness and Social Dissatisfaction Scale. Sociodemographic information included sex (male, female), race/ethnicity (white, non-white), age at assessment, and household income (under CAD 50,000, over CAD 50,000).

Impact on Practice or Results

Linear regression tested whether sociodemographic factors were associated with loneliness scores in survivors and controls. Model 1 included cancer status and was significant, *F*(1, 66) = 5.88, *p* = 0.018, *R^2^* = 0.082, with PBTS reporting a loneliness score 3.87 points higher than controls. Sex, race/ethnicity, age, and income were added as main effects (Model 2) and as interactions with cancer status (Model 3). These models were non-significant, *p* > 0.05.

Discussion or Conclusions

With higher loneliness found in PBTS, survivors may need support with social adjustment. There were limited numbers of racial/ethnic minority (*n* = 16) and middle/low-income (*n* = 7) participants, which may have affected the analyses. Future research should examine sociodemographic factors in a more diverse population of survivors.

108

### 7.4. The BOLD Sidewalks-to-Screening Program: Assessing Barriers to Cancer Screening among the Underserved Community of Bronx, NYC

Johnna Bakalar, Brittany Miller, Christina Martinez and Alyson Moadel-Robblee

Albert Einstein College of Medicine, Bronx, NY, USA

Background/Rationale or Objectives/Purpose

During COVID-19, the Bronx saw disruption in cancer screening, with mammography rates dropping over 20%. The BOLD Sidewalks-to-Screening (S2S) Program at Montefiore Einstein Cancer Center in Bronx, NY serves one of the most underserved and diverse constituencies in the nation, with 31% in poverty and 34% foreign-born. The program seeks to elucidate barriers to screening uptake contributing to cancer disparities in these marginalized populations.

Methodology or Methods

Patient navigators administered a telephone survey to Bronx residents engaged by the S2S team at previous community events. Assessment included demographics, cancer screening uptake, and barriers and facilitators to screening.

Impact on Practice or Results

Respondents (*n* = 259) identified as Black (44%), Hispanic (53%), foreign-born (38%), non-English dominant (44%), and female (90%). Among females age-eligible for a mammogram (*n* = 173) or pap smear (*n* = 223), those foreign-born had almost four times the odds of having never received a mammogram (22% OR = 3.96, *p* = 0.003) or pap-smear (20% OR = 3.97, *p* = 0.001) than those US-born. In females (*n* = 227), not having insurance (c2 = 6.325, *p* = 0.012) and a regular primary care physician (c2 = 7.002, *p* = 0.008) were associated with being foreign-born. A significantly lower percentage of foreign-born females reported a family cancer history (c2 = 14.990, *p* < 0.001), which may lower perceived risk, and a significantly higher percentage endorsed concerns about immigration status, language, or religion as a screening barrier (c2 = 6.706, *p* = 0.01).

Discussion or Conclusions

Closing gaps in mammography and pap-smears amongst Bronx immigrants is critical to reducing disparities in cancer. Efforts connecting immigrant women to insurance and culturally competent primary care are necessary. Future directions will focus on ways to engage men in BOLD’s S2S program.

109

### 7.5. Experiences of Invisibility among Young Women Living with Multiple Myeloma: A Call for More Patient-Centered Approaches to Healthcare Organization and Delivery

Cheryl Pritlove ^1,2^, Jannah Wigle ^1^, Mathankki Ramasamy ^1^, Hira Mian ^3,4^, Irwindeep Sandhu ^5,6^ and Arleigh McCurdy ^7^

1. Unity Health Toronto, Toronto, Canada
2. University of Toronto, Toronto, Canada
3. Juravinski Cancer Center, Hamilton, Canada
4. McMaster University, Hamilton, Canada
5. University of Alberta, Edmonton, Canada
6. Cross Cancer Institute, Edmonton, Canada
7. Ottawa Hospital Research Institute, Ottawa, Canada

Background/Rationale or Objectives/Purpose

Multiple myeloma (MM) is an incurable cancer typically diagnosed in an older population, however, approximately 16% of cases occur in patients younger than 50 years of age. The ways in which age, life stage, sex and gender intersect to frame the experiences and care needs of young women living with MM remain underexplored.

Methodology or Methods

We conducted semi-structured interviews with 15 young women to understand their experiences of diagnosis and treatments, care needs, and the impact of living with MM on their quality of life. Transcripts were coded using a reflexive thematic analysis approach.

Impact on Practice or Results

Participants emphasized the intersections of age, life stage, sex, and gender in framing their experience living with MM. Participants often viewed their experiences as “invisible” from a health systems perspective, as they described poorly coordinated, non-personalized, and inadequate care and supports to cope with their unique physical and psychosocial needs and concerns. Participants provided recommendations for system improvements aimed at better reflecting the unique experiences, challenges, and supportive care needs of young women living with MM.

Discussion or Conclusions

Despite highly individual experiences, women also illuminated a shared collection of concerns that rallied around their social locations as young women living with an incurable illness. The invisibility of young women’s experiences living with MM, and the subsequent lack of care, resulted in avoidable physical and psychosocial suffering for these women. It is imperative that health systems adapt to better address the needs of this population, in part, by partnering more effectively with them in the organization and delivery of care.

116

### 7.6. SexOnco a Mixed-Method Pilot Study to Evaluate an Educational Intervention to Improve Sexual Health Nursing Competencies with Adults Living with Cancer

Hazar Mrad, Maria-Pilar Ramirez Garcia and Karine Bilodeau

Faculté des sciences infirmières de l’Université de Montréal, Montreal, Canada

Background/Rationale or Objectives/Purpose

To develop and evaluate the feasibility, acceptability and preliminary effects on sexual health attitudes, beliefs and competencies of an online educational intervention “SexOnco” for nurses caring for adults living with cancer.

Methodology or Methods

A pilot study with a mixed method will be conducted with 30 nurses caring for adult living with cancer. The intervention will be developed in collaboration with clinical experts, such as a sexologist, and patient partners. It will include readings, group discussions and online simulations with patient partners on ZOOM. For the quantitative component, a non-experimental design and self-reported questionnaires will be used. For the qualitative component, a descriptive qualitative design will include semi-structured interviews and analytic questioning. The quantitative and qualitative data will then be juxtaposed in a matrix format and tested to explore convergence or divergence of results.

Impact on Practice or Results

The results of this study regarding the feasibility, acceptability, and preliminary effects of the “SexOnco” intervention could provide the basis for a larger, randomized controlled clinical trial. This project could provide knowledge to stimulate changes in the continuing nursing education curriculum and possibly an update of academic nursing education that remains suboptimal in sexual health.

Discussion or Conclusions

There is a need to optimize the continuing nursing training in sexual health with cancer patients. This study proposes an innovative educational intervention to improve nursing competencies on sexual health after cancer.

## 8. Final Category: F. Digital Health and Cancer Care

35

### 8.1. An Online Adaptation of Managing Cancer and Living Meaningfully (iCALM): A Phase II Randomized Controlled Trial

Laurice Karkaby ^1^, Sarah Hales ^1^, Carmine Malfitano ^1^, Anne Rydall ^1^, Rinat Nissim ^1^, Natalie Bauereiß ^2^ and Gary Rodin ^1^

1. Princess Margaret Cancer Centre, Toronto, Canada
2. University of Ulm, Ulm, Germany

Background/Rationale or Objectives/Purpose

Patients with advanced cancer are at risk of depression which decreases quality of life, adherence to treatment and increases the risk of suicide. Psychotherapy can be effective but most patients do not receive it due to a lack of tailored therapies or trained clinicians. To address this issue, we developed a novel psychotherapy intervention called Managing Cancer and Living Meaningfully (CALM) to prevent and treat depression in patients with advanced disease. In a large randomized controlled trial, we demonstrated CALM reduces or prevents depression in these patients. However, since access to trained clinicians is limited, we developed an online version referred to as iCALM. The aim of this study is to test iCALM’s feasibility and preliminary efficacy.

Methodology or Methods

A total of 50 patients at Princess Margaret (PM) will be randomized (25/group) to receive either iCALM or usual care. Feasibility and efficacy will be determined via questionnaires assessing intervention satisfaction, depression, death anxiety, and quality of life (baseline, 4, 8 and 12 weeks). A subgroup of patients will be interviewed.

Impact on Practice or Results

A total of 32 patients are enrolled with data collection to conclude in Spring 2023. Preliminary evidence from interviews supports iCALM as providing a framework for patients to manage their diagnosis, and that patients feel supported by their eCoach.

Discussion or Conclusions

This trial has the potential not only to enhance the psychological dimensions of early palliative care to alleviate depression and other psychological distress in patients with advanced cancer, but also to be a vanguard for the development, evaluation, and implementation of internet- and mobile-based interventions in psychosocial oncology.

50

### 8.2. High-Fidelity Testing in a Smartphone App to Treat Insomnia in Cancer Survivors (iCANSleep App)

Katherine-Ann Piedalue and Sheila Garland

Memorial University, St. John’s, Canada

Background/Rationale or Objectives/Purpose

Insomnia in cancer survivors is highly prevalent, long-lasting, and debilitating, impacting individuals’ mood, physical health, and perceived quality of life. Although cognitive behavioural therapy for insomnia (CBT-I) is a well-known therapeutic practice with substantial efficacy, only a small percentage of clinicians have received formal training. Technology-based solutions are one way to increase accessibility and decrease barriers for cancer survivors. To fill this gap, we are adapting an evidence-based insomnia treatment, CBT-I, to a smartphone-based app called iCANSleep. The iCANSleep app is uniquely designed with patient advocates to meet the specific needs of cancer survivors. Patient stories will also be included to help patients “see themselves” in the app, which may increase the useability and enjoyment of the program. The goal of this research during the development phase is to collect patient feedback on a high-fidelity version of the app.

Methodology or Methods

A purposive sample of 15 cancer patients with a score of 8 or more on the Insomnia Severity Index (ISI) will be recruited. Participants will be recorded while interacting with the app and verbally describing their experience, including likes, dislikes, and roadblocks.

Impact on Practice or Results

Participants in this study will be able to provide meaningful feedback that will help optimize the ease and use of the iCANSleep app.

Discussion or Conclusions

iCANSleep app will increase access to care for cancer survivors, improving quality of life and health outcomes. Future research will continue to improve the useability of the iCANSleep prototype before starting Phase 3: feasibility, acceptability, and preliminary efficacy testing of the larger research project.

51

### 8.3. Adverse Events in a Randomized Controlled Trial of Cognitive Behavioral Therapy for Insomnia in Patients with Perceived Cancer-Related Cognitive Impairments

Joshua Tulk ^1^, Harsha Ajith ^1^, Kris Aubrey-Bassler ^2^, Scott V Harding ^3^, Bob Wakeham ^1^, Yanqing Yi ^4^ and Sheila N Garland ^1,5^

1. Department of Psychology, Faculty of Science, Memorial University, St. John’s, NL, Canada
2. Discipline of Family Medicine, Faculty of Medicine, Memorial University, St. John’s, NL, Canada
3. Department of Biochemistry, Faculty of Science, Memorial University, St. John’s, NL, Canada
4. Division of Community Health and Humanities, Faculty of Medicine, Memorial University, St. John’s, NL, Canada
5. Discipline of Oncology, Faculty of Medicine, Memorial University, St. John’s, NL, Canada

Background/Rationale or Objectives/Purpose

Insomnia and perceived cognitive impairments (PCI) are frequent symptoms in cancer survivors. This study describes adverse events (AEs) experienced by cancer survivors who completed a randomized controlled trial of cognitive behavioural therapy for insomnia (CBT-I) to treat PCI.

Methodology or Methods

One-hundred thirty-two Atlantic Canadian cancer survivors with insomnia and PCI completed seven sessions of CBT-I as part of a clinical trial. The CBT-I intervention involved a phase of stimulus control, sleep hygiene, sleep restriction, relaxation training, and cognitive therapy. Participants completed open-ended questions about AEs mid- and post-treatment and were asked to report their severity and perceived association with CBT-I. Participant responses were coded using inductive methods and analyzed for repetitive themes. Descriptive statistics were used to describe reports of AEs during and after CBT-I.

Impact on Practice or Results

A total of 72 participants (55% of the total sample) reported 207 AEs during treatment; however, only 31 (15%) were attributed to treatment, and 52% were reported to be mild. The most common AEs related to treatment were changes in the ability to handle life stress, physical symptoms (i.e., increased headaches, consuming more pain medication), and psychological symptoms (i.e., stress related to treatment protocol). Participants reported fewer AEs attributed to treatment at post-treatment (7 AEs) compared to mid-treatment (24 AEs). Of study withdrawals (*n* = 20, 15%), 15% were attributed to AEs related to treatment (treatment too stressful (*n* = 2) and experiencing a mental health crisis (*n* = 1)). Only one participant reported a severe adverse event requiring medical attention (increased joint pain related to treatment).

Discussion or Conclusions

CBT-I appears safe and well tolerated by cancer survivors.

63

### 8.4. Optimizing Virtual Follow-Up Care for Breast and Prostate Cancer Patients: A Realist Evaluation

Sarah Scruton ^1,2^, Stephanie Babinksi ^3^, Lauren Squires ^4^, Geoffrey Wong ^5^, Alejandro Berlin ^1,4^, Julie Easley ^6^, Sharon McGee ^7^, Danielle Rodin ^1,4^, Jonathan Sussman ^8^, Robin Urquhart ^2,9^ and Jacqueline Bender ^1,4^

1. University Health Network, Toronto, Canada
2. Nova Scotia Health Authority, Halifax, Canada
3. Throughline Strategy Inc, Toronto, Canada
4. University of Toronto, Toronto, Canada
5. Oxford University, Oxford, UK
6. Horizon Health Network, Fredericton, Canada
7. The Ottawa Hospital Cancer Center, Ottawa, Canada
8. McMaster University, Hamilton, Canada
9. Dalhousie University, Halifax, Canada

Background/Rationale or Objectives/Purpose

Virtual follow-up (VFU) has the potential to enhance survivorship care. However, a greater understanding is needed of how VFU can be optimized for patients. This study examined how, for whom, and in what contexts VFU works for patients.

Methodology or Methods

We conducted a realist evaluation of VFU among breast cancer (BC) and prostate cancer (PC) patients at an urban cancer centre during the COVID-19 pandemic. Realist evaluations examine how underlying processes of an intervention (mechanisms) in specific circumstances (contexts) interact to produce results (outcomes). Semi-structured interviews were conducted with a purposive sample of post-treatment patients. Interviews were audio-recorded and analysed using a realist logic of analysis.

Impact on Practice or Results

Participants (*n* = 24; 12 BC, 12 PC) were on average 59.6 years-of-age (SD = 10.7) and ≤5 years post-diagnosis. The majority (83.3%) were satisfied with VFU and wanted it to continue after the pandemic. However, VFU impacted on patient perceptions of the quality of their care, particularly in terms of its effectiveness and patient-centeredness. Whether VFU worked well for patients depended on: patient factors (e.g., needs, psychosocial well-being, technological competence), provider factors (e.g., socioemotional behaviours, technological competence), and virtual care system factors (e.g., usability, functionality, communication). Key mechanisms that interacted with contexts to produce positive outcomes (e.g., satisfaction) were visual cues, clear communication, and a trusting relationship with their provider.

Discussion or Conclusions

Patients valued VFU; however, it is not working for patients as well as it could. To optimize VFU, we need to consider the contexts and mechanisms that impact on patient perceptions of the effectiveness and patient-centredness of their care.

67

### 8.5. Establishing Best Practices in Online Cancer Support Groups: A Realist Review

Jackie Bender ^1^, Sarah Scruton ^1^, Stephanie Babinski ^1^, Lauren Squires ^1^, Geoff Wong ^2^, Mary Jane Esplen Esplen ^3^, Janet Papadakos ^1^, Olga Perski ^4,5^, Henry Potts ^6^, Charlene Soobiah ^3^, Andrea Tricco ^7^ and Holly Witteman ^8^

1. Princess Margaret Cancer Centre, University Health Network, Toronto, Canada
2. Oxford University, Oxford, UK
3. University of Toronto, Toronto, Canada
4. University of California San Diego, San Diego, USA
5. Tampere University, Tampere, Finland
6. University College London, London, UK
7. Unity Health, Toronto, Canada
8. Laval University, Quebec City, Canada

Background/Rationale or Objectives/Purpose

Online support groups (OSGs) can be a convenient way for cancer patients and survivors to obtain peer support. However, OSGs may not help everyone, and, in some cases, may lead to negative outcomes. It is currently unclear how OSGs work, for whom, and why.

Methodology or Methods

We conducted a realist review of the literature (12 databases; inception to 5 December 2020) on cancer OSGs. Realist reviews examine how underlying processes of an intervention (mechanisms) in specific circumstances (contexts) interact to produce results (outcomes). A program theory was developed based on the review to explain how cancer OSGs work.

Impact on Practice or Results

Of 9,276 articles identified, 154 were included. We found that cancer OSGs can be an effective tool for providing emotional, informational, validation, and altruistic support. This can lead to changes in distress, isolation, empowerment, and self-esteem, through negative and positive appraisals as well as coping efforts. These outcomes, however, depend on user attitudes about OSGs, how well the OSG fits their needs, and user perceptions of control (e.g., availability, privacy, anonymity). If an OSG was a good fit for their needs (e.g., relevant, and relatable), whether users experienced positive outcomes depended on the communication technology (e.g., response time, visual cues), group dynamics (e.g., norms, moderation, connectedness), and content (e.g., emotional expression, cognitive processing).

Discussion or Conclusions

OSGs can be an effective source of support for cancer patients and survivors and can improve psychosocial well-being. However, outcomes depend on specific contexts and mechanisms that impact on how well cancer OSGs meet the needs of the users.

79

### 8.6. A Cross-Sectional Study of Patients, Caregivers, and Healthcare Providers’ Perceptions of BELONG—A Cancer Navigation and Support Digital Health Platform

Saima Ahmed ^1,2^, Renata Benc ^2^, Karine LePage ^2^, Gabrielle Chartier ^2^, Alon Litvin ^3^, Guy Erez ^3^ and Carmen G. Loiselle ^1,2^

1. McGill University, Montreal, Canada
2. CIUSSS du Centre-Ouest-de l’Île-de Montréal, Montreal, Canada
3. Belong.life Inc, New York, USA

Background/Rationale or Objectives/Purpose

A closed community (CC), a supportive and educational area exclusively for patients and their caregivers at a participating cancer center, was developed and launched using BELONG—Beating Cancer Together (https://cancer.belong.life/, accessed on 10 March 2023), a person-centred digital health platform. Post implementation, this cross-sectional study seeks to explore platform perceptions from the perspectives of diverse groups of users.

Methodology or Methods

Patients, caregivers, and healthcare providers (estimated: *n =* 332) from a large university-affiliated cancer centre in Montreal who downloaded BELONG on their mobile device or tablet will be invited to take part in this study. All potential participants will receive a notification from BELONG on their device inviting them to complete an online survey and consent form. Consenting participants will be directed to complete self-report online surveys including the user Mobile Application Rating Scale (uMARS), assessing apps’ levels of engagement, functionality, aesthetics, and information, as well as questions relating to clinical management (e.g., side effects, emergency room visits) and a sociodemographic sheet.

Impact on Practice or Results

Data collection and analysis are estimated to be completed by May 2023. A focus will be on participant profiles: user (patient, caregiver, or healthcare provider), gender, cancer type, age group, and content viewed in relation to their app’s quality ratings.

Discussion or Conclusions

Findings will be used to gain insights into BELONG’s CC contributions and potential areas within the platform in need of refinement.

100

### 8.7. Transforming Cancer Care in Nova Scotia: Remote Symptom Management and beyond

Katrin Julia Kaal ^1,2^, Marianne Arab ^3^, Joy Tarasuk ^3^, Helmut Hollenhorst ^3^, Margaret Ann Morrison ^3^, Carolyn Fifield ^3^, Sue Chisholm ^3^, Bruce Colwell ^3^ and Amanda Caissie ^1,3^

1. Dalhousie University, Halifax, Canada
2. Nova Scotia Health Authority, Halifax, Canada
3. Cancer Care Program Nova Scotia Health Authority, Halifax, Canada

Background/Rationale or Objectives/Purpose

The Oncology Transformation Project (OTP) is an initiative being conducted within the Nova Scotia Health Cancer Care Program in partnership with Varian Medical Systems. Its goal is to redesign the delivery of cancer care, ensure the highest quality of care with improved operational efficiency and new, modernized, and integrated software tools for clinicians and patients to enhance a connected model of care delivery across the two cancer centers and eight community-based oncology sites in the province. System functionality will include the expansion of an electronic patient-reported outcomes (ePROs) tool (Noona), that previously was used exclusively within the Department of Radiation Oncology.

Methodology or Methods

A phased, accelerated approach for the operationalization and implementation of the ePRO tool Noona into scheduling and the clinical workflow is led by the Virtual Care and Patient Engagement working group, with representation of NSH CCP and Varian stakeholders.

Impact on Practice or Results

The expansion of Noona to all cancer patient populations in Nova Scotia will facilitate real-time reporting of treatment toxicities and side effects by patients directly to providers, eliminating incomplete, delayed, and biased reporting. Proactive symptom assessment and management by healthcare providers as well as the delivery of tailored patient education will transform the care experience for patients and providers, improve cancer care outcomes, and improve access to timely and personalized cancer care, thereby reducing unscheduled healthcare utilization.

Discussion or Conclusions

Noona will support remote symptom management, advancing care closer to home and empower patients to manage their disease and cancer treatment-related symptoms from home.

## 9. Final Category: G. Exercise/Pre-Habilitation and Rehabilitation in Cancer

15

### 9.1. Participant Perspectives on Using a Self-Monitoring App to Support Physical Activity Maintenance after an Exercise Oncology Program: Qualitative Findings from a Cluster-Randomized Controlled Trial

Manuel Ester ^1^, Mannat Bansal ^1^, Julia T. Daun ^1^, Thompson Luu ^1^, Julianna Dreger ^1^, Margaret L. McNeely ^2,3^, Meghan H. McDonough ^1^ and S. Nicole Culos-Reed ^1,4^

1. Faculty of Kinesiology, University of Calgary, Calgary, Canada
2. Department of Physical Therapy, University of Alberta, Edmonton, Canada
3. Department of Oncology, University of Alberta, Edmonton, Canada
4. Department of Oncology, Cumming School of Medicine, University of Calgary, Calgary, Canada

Background/Rationale or Objectives/Purpose

Health technology (eHealth) interventions can help increase physical activity (PA) and thereby improve health among cancer populations. To date, the literature on the effectiveness of eHealth exercise oncology interventions is mixed. Qualitative studies may provide further understanding on the use of eHealth. The present study explored participant perspectives on ease of use and perceived value of an eHealth app to support PA habits during and after an exercise oncology intervention.

Methodology or Methods

Participants used a self-monitoring app throughout a 12-week PA intervention and 12-week post-intervention maintenance phase. Semi-structured interviews after 24-weeks examined participants’ perspectives on ease-of-use and value of the app. Convenience (interview rounds 1–2) and purposive (interview rounds 3–5, targeting diversity based on age, gender, cancer type) sampling was used. Interview transcripts were analyzed using interpretive description.

Impact on Practice or Results

Participants (*n* = 18) described barriers and facilitators to ease-of-use and perceived value of the app for supporting PA. Facilitators (e.g., simplicity, PA goal-setting) prompted continued use, increasing accountability to PA. Barriers (e.g., time consuming, lacking personal relevance) contributed to decreased use. Lastly, participants shared ideas for making the app more user-friendly and valuable (e.g., inter-app integration, providing weekly PA summaries).

Discussion or Conclusions

The self-monitoring app was described as a valuable tool for supporting PA, especially for participants with greater PA barriers, unmet support needs, and familiarity with technology. However, it may be less valuable for those with established PA habits, fewer PA barriers, or preferred pre-existing self-monitoring methods. Improved user-friendliness and added value via “smart” health insights could enhance the app’s potential to support PA.

90

### 9.2. Examining the Relationship between Physical Activity, Pain Interference, and Psychosocial Well-Being in Patients Receiving Care at a Long-Term Survivor Clinic

Favour Omobhude ^1^, Kathleen Reynolds ^2^, Jennifer Giles ^2^, Mehak Stokoe ^1^, Brianna Henry ^1^ and Fiona Schulte ^1,2^

1. University of Calgary, Calgary, Canada
2. Alberta Children’s Hospital, Calgary, Canada

Background/Rationale or Objectives/Purpose

To describe the relationship between physical activity and pain interference, and their impact on the levels of psychosocial functioning of patients attending a long-term survivor clinic (LTSC).

Methodology or Methods

Survivors of childhood cancer and blood/marrow transplant (BMT) attend annual or bi-annual medical follow-up appointments at the Alberta Children’s Hospital LTSC. Prior to each clinic appointment, parents complete a questionnaire probing their child’s health status (i.e., pain interference, physical activity) and psychosocial well-being (i.e., depression, anxiety, peer relations). Pain interference, depression, anxiety, and peer relations were measured using PROMIS Short Form questionnaires. Correlations were used to determine if pain interference and physical activity were associated with psychosocial well-being. Significant associations were followed up with linear regressions.

Impact on Practice or Results

A total of 321 parents of survivors (42.1% male, mean age = 11.5 years, *SD* = 4.3) completed proxy reports. Pain interference scores differed based on patients’ level of physical activity (*F* (23, 200) = 2.3, *p* = 0.002). Patients who frequently participated in physical activities, three or more times a week, had lower depression (*p* = 0.03), lower anxiety (*p* = 0.011), and higher peer relationship scores (*p* = 0.003). Pain interference scores could predict patients’ depression scores (β = 0.354, *p* < 0.0005) but were unable to predict anxiety and peer relationship scores.

Discussion or Conclusions

The frequency of physical activity had significant positive clinical implications for pediatric cancer survivors. Pain interference scores predicted depression scores, indicating the importance of addressing pain management in improving emotional well-being. Interventions at the intersection of pain management and physical activity may improve physical and psychosocial well-being in survivors of pediatric cancer.

98

### 9.3. The Feasibility of a Tailored Neuro-Oncology Exercise Program: Work to Date

Julia T. Daun ^1^, Lauren C. Capozzi ^1,2^, Meghan H. McDonough ^1^, Gloria Roldan Urgoiti ^3^, Jacob C. Easaw ^4^, Margaret L. McNeely ^5,6^, George J. Francis ^2,7^, Tana Dhruva ^1^, Tanya Williamson ^1^, Jessica Danyluk ^1^, Emma McLaughlin ^1^, Paula A. Ospina ^5^, Elaine Gobeil ^5^, Christopher Sellar ^5^, Christine Lesiuk ^3^ and Nicole Culos-Reed ^1,7,8^

1. Faculty of Kinesiology, University of Calgary, Calgary, Canada
2. Department of Clinical Neurosciences, Cumming School of Medicine, University of Calgary, Calgary, Canada
3. Department of Medical Oncology, Tom Baker Cancer Centre, Alberta Health Services, Calgary, Canada
4. Department of Medical Oncology, Cross Cancer Institute, Edmonton, Canada
5. Department of Physical Therapy, University of Alberta, Edmonton, Canada
6. Department of Oncology, Cancer Care Alberta, Edmonton, Canada
7. Department of Oncology, Cumming School of Medicine, University of Calgary, Calgary, Canada
8. Department of Psychosocial Resources, Tom Baker Cancer Centre, Alberta Health Services, Calgary, Canada

Background/Rationale or Objectives/Purpose

Exercise is an evidence-based tool that can support the well-being of neuro-oncology patients, yet it is not included as part of standard neuro-oncology care. To understand implementation needs for exercise across the neuro-oncology cancer continuum, the Alberta Cancer Exercise-Neuro-Oncology (ACE-Neuro) study is examining the feasibility of a tailored exercise intervention for this patient population.

Methodology or Methods

Neuro-oncology patients who are >18 years old, able to consent in English, and treated across Alberta are eligible for the ACE-Neuro-Study. Patients can either (1) be referred via a clinician using the electronic medical record or (2) self-refer to the study. The exercise intervention includes 12 weeks of tailored one-on-one and group-based sessions, and health coaching. Participants complete patient-reported outcomes and tests of functional fitness pre- and post-intervention and at 6- and 12-month follow-ups, as well as wear a Garmin activity tracker across the intervention. Criteria for determining feasibility include minimum rates for referral (≥50%), enrolment (≥50%), program adherence (≥50%), and measurement completion (≥60% for pre- and post-intervention; ≥40% for 6- and 12-month follow-ups), and no major adverse events.

Impact on Practice or Results

Recruitment occurred from April 2021 to December 2022 and *n* = 70 patients enrolled (31% referral rate; 65% enrolment rate). To date, *n* = 46 participants have completed the intervention and no major adverse events have occurred. Intervention delivery and collection of adherence and measurement completion rates is ongoing until Winter 2024.

Discussion or Conclusions

ACE-Neuro is a novel exercise intervention for a clinically underserved patient population. Assessing the feasibility of ACE-Neuro is critical for implementation of a clinic-supported exercise program across the neuro-oncology care pathway.

## 10. Final Category: H. Implementation Science, Knowledge Translation and Synthesis

26

### 10.1. Improving Evidence-Informed Symptom Management Delivery in Outpatient Malignant Hematology Care: Protocol for an Experience-Based Co-Design Study

Kylie Teggart, Denise Bryant-Lukosius, Sarah Neil-Sztramko, Nancy Carter and Rebecca Ganann

McMaster University, Hamilton, Canada

Background/Rationale or Objectives/Purpose

Objectives/Purpose: To address research-to-practice gaps influencing the delivery of evidence-informed symptom management in outpatient malignant hematology care by (1) understanding patients’ and oncology nurses’ symptom management experiences, (2) establishing consensus on priorities for service improvements, and (3) co-designing strategies to address current challenges and leverage existing strengths related to the implementation of cancer symptom management guidelines.

Methodology or Methods

Methods: A novel experience-based co-design approach will be used. A Steering Group of patient, caregiver, nursing, and organizational knowledge users will inform all study phases. A scoping review will identify the types and components of strategies used to overcome symptom management guideline implementation barriers in cancer-specific outpatient settings. A qualitative descriptive study will include patients (*n* = 12–15) and oncology nurses (*n* = 12–15) in an outpatient malignant hematology setting. Patient interviews will be videotaped and edited into a compilation video highlighting touchpoints shaping patients’ experiences with the quality of symptom management care. Interviews with nurses will identify factors influencing evidence-informed symptom management delivery based on the Consolidated Framework for Implementation Research.

Impact on Practice or Results

Results: Findings will be shared with the Steering Group to establish consensus on service improvement priorities that are of high relevance to patients, nurses, and the organization. Co-design meetings will aim to develop tailored strategies necessary to support evidence-informed symptom management delivery.

Discussion or Conclusions

Conclusions/Clinical Implications: Hematologic cancer symptom burden is distressing and often unmanaged. Using experience-based co-design, this study will result in contextually relevant, evidence-informed strategies to improve patient/clinician experiences, health system performance, and symptom burden for individuals living with hematologic malignancies.

64

### 10.2. Implementing Distress Screening in a Diverse, Multi-Site Oncology Network: Optimizing Alignment among Collaborating Partners

Carrie Mazoff ^1^, Renata Benc ^2^, Marie-Solange Bernatchez ^3^, Catherine Bigras ^2^, Christine Bouchard ^3^, Maya Jeanty ^4^, Tina Kusaian ^3^, Joséphine Lemy Dantica ^4^, Karine Lepage ^2^ and Jeff Mangerpan ^4^

1. Rossy Cancer Network, Montreal, Canada
2. CIUSSS du Centre-Ouest-de-l’Île-de-Montréal (JGH), Montreal, Canada
3. McGill University Health Centre, Montreal, Canada
4. CIUSSS de l’Ouest-de-l’Île-de-Montréal (SMH), Montreal, Canada

Background/Rationale or Objectives/Purpose

The benefits of screening for distress among oncology patients using patient-reported outcomes (PROs) have been well-documented in the literature. Implementation of this, however, can be very complex. The Rossy Cancer Network (RCN), comprised of three McGill hospitals, undertook the “PROMISE” project in order to ensure comprehensive and proactive symptom management across its network.

Methodology or Methods

The RCN hospitals have worked intensely over the last few years to collaborate in building the distress screening program, following the same vision and common principles. Relying on best practice and the literature for guidance, and balancing consistency with flexibility helped to ensure buy-in from all sites and clinicians with differing viewpoints. Change management was especially important, to support clinicians in integrating PROs into practice and ensuring optimal documentation of interventions offered.

Impact on Practice or Results

Each hospital tailored their approach to their own environments, constraints, and goals to achieve their own path to success. This “healthy tolerance” for variation led to full participation, partner engagement, and productive knowledge-sharing along the way (among network sites). Despite the challenges inherent in this type of implementation, acceptance and culture change around the importance of distress screening has evolved very positively since the inception of the project, and the momentum continues.

Discussion or Conclusions

One key lesson learned is that uniformity of the platform (software) for screening is not critical, but rather consistency in the questionnaires and ultimate project goals is what matters. Next steps include the ongoing implementation (wider rollout) and program adjustments. We would also like to build a pan-Canadian community of practice for distress screening.

71

### 10.3. Availability and Characteristics of Psychosocial Oncology Training across Canada

Alexei Bardine ^1^, Catherine Bergeron ^1^, Mary-Clare Booth ^2^ and Annett Körner ^1^

1. McGill, Montreal, Canada
2. Department of Psychology, McGill University, Montreal, Canada

Background/Rationale or Objectives/Purpose

Psychosocial oncology services support patients in coping with the psychosocial challenges of cancer and are an essential component of comprehensive cancer care. However, little is known about training opportunities in Canada for graduate students and healthcare professionals seeking basic or advanced training to prepare for working in psychosocial oncology.

Methodology or Methods

Selected courses must primarily focus on psychosocial oncology and be offered by an organization within Canada. A two-step environmental scan was conducted to address this knowledge gap. The first step consisted of a targeted search of websites for training courses and programs offered by special interest organizations and Canadian universities. The second step consisted of a search of non-indexed sources through a generic Internet search engine using relevant keywords drawn from the literature.

Impact on Practice or Results

Our search identified 25 training programs and courses specific to psychosocial oncology. Half of them consisted of university-level courses, 24% were fellowships or internships offered in conjunction with medical centers, 16% were continuing education courses, and 8% were online workshops/webinars. A detailed breakdown of the trainings’ characteristics, such as prerequisites, costs, duration, scheduling, and location, will be provided.

Discussion or Conclusions

Most courses and training programs can be completed online, which facilitates accessibility for clinicians across Canada. Making our findings available on the CAPO website will provide a centralized resource for seeking information on available training opportunities. The generated knowledge will also inform the design of future training, for example, by identifying current gaps in Canadian psychosocial oncology training opportunities.

76

### 10.4. Patient Adherence to Oral Anticancer Agents: A Mapping Review of Supportive Interventions

Saima Ahmed ^1,2^ and Carmen G. Loiselle ^1,2^

1. McGill University, Montreal, Canada
2. Segal Cancer Centre, CIUSSS du Centre-Ouest-de l’Iˆle-de Montreal, Montreal, Canada

Background/Rationale or Objectives/Purpose

As the development and use of oral anti-cancer agents (OAAs) continues to grow, supporting individuals on OAAs has emerged as a challenge. A mapping review was undertaken to capture the body of literature on supportive interventions promoting OAA adherence.

Methodology or Methods

Key databases (PubMed/MEDLINE, EMBASE, CINAHL, PsycINFO) and the grey literature were searched for publications from January 2010 to January 2023. Quantitative, qualitative, mixed-method studies, theses/dissertations, reports, and abstracts were included; study protocols and review articles were excluded. The search strategy included concepts of oral chemotherapy, intervention, and medication adherence, related subject headings/keywords (e.g., oral cancer agent*/therapy*/treatment*/drug*). Duplicates were removed and remaining articles were screened by title and abstract. Full-text articles were assessed for eligibility, those meeting inclusion criteria were retained for synthesis. Data extracted included study design, OAA intervention type, theoretical underpinnings, primary outcome(s), patient population, and sample size.

Impact on Practice or Results

A total of 3175 articles were identified, 435 were screened, 314 were excluded for duplicates and not meeting inclusion criteria, and 121 articles were retained. OAA interventions reported were pharmacist-led (*n* = 41), digitally based (*n* = 26), nurse-led (*n* = 21), multidisciplinary (*n* = 22), digital/care provider combined (*n* = 8). Frequently assessed outcomes were medication adherence/compliance (*n* = 53), toxicity/symptoms/side effects (*n* = 12), or both (*n* = 12). In terms of design, of the 121 studies, non-experimental (e.g., retrospective) were the most frequent (*n* = 70), followed by quasi-experimental (*n* = 31), and randomized controlled trials (*n* = 20).

Discussion or Conclusions

Mapping review findings suggest that more multi-modal approaches should be tested with rigorous designs to provide higher quality evidence on the role of OAA supportive interventions.

91

### 10.5. Implementation of a Sexual Health Clinic in an Oncology Setting

Andrew Matthew ^1^, Taylor Incze ^1^, Elisa Stragapede ^1^, Steven Guirguis ^1^, Sarah Peltz ^2^ and Dean Elterman ^3^

1. Department of Surgical Oncology, Princess Margaret Cancer Centre, University Health Network, Toronto, Canada
2. Division of Urology, Department of Surgery, Mackenzie Health, Richmond Hill, Canada
3. Division of Urology, Department of Surgery, University Health Network, University of Toronto, Toronto, Canada

Background/Rationale or Objectives/Purpose

Sexual health concerns are some of the most prevalent and challenging side effects of cancer treatment. A hybrid virtual and in-person Sexual Health Clinic (SHC) was recently established at a high-volume Cancer Centre to provide patients with comprehensive sexual health assessment and intervention. Previous research has highlighted the importance of implementation in the achievement of successful outcomes. As part of a robust program evaluation, implementation research methods were used to inform the successful integration of the SHC.

Methodology or Methods

The implementation of SHC was guided by the Quality Implementation Framework comprising the identification of unique implementation factors within each cancer-site specific clinic. The procedure involved conducting 30-min, semi-structured interviews with 20 clinic representatives and patient partners. A pragmatic qualitative examination was used to analyze the interview transcripts and inform the evolution of successful SHC integration strategies.

Impact on Practice or Results

Analysis of the key stakeholder interviews produced five organizing themes that best capture essential implementation processes. These include: Known Gap in Care, Confinements of Current Care, Unique Needs by Cancer Site, Collaborative Stakeholder Integration, and Strategic Implementation. Subthemes include: Validation of Patient Concerns, Stigmatized Avoidance, Fear of the Unknown, and Mutually Established Helplessness. The outcomes of this study provide pragmatic insight into key stakeholder enablers and barriers to acceptability, adoption, engagement, feasibility, fidelity, penetration and sustainability.

Discussion or Conclusions

Understanding implementation variables is fundamental to the successful delivery of sexual health assessment and intervention within a high-volume Cancer Centre.

113

### 10.6. Implementation of Proactive PSO: Enhancing access to Psychosocial Oncology Care for Vulnerable Patients

Pragash Loganathan ^1,2^, Richa Srivastava ^1^, Alyssa Macedo ^1^, Janet Papadakos ^1,3^, Megan Wexler ^1^, Mike Lovas ^1^, Robin Forbes ^1^, Lesley Moody ^1^ and Madeline Li ^1,4^

1. Princess Margaret Cancer Center, Toronto, Canada
2. Institute of Medical Science, Temerty Faculty of Medicine, University of Toronto, Toronto, Canada
3. Institute of Health Policy, Management and Evaluation, University of Toronto, Toronto, Canada
4. Department of Psychiatry, Faculty of Medicine, University of Toronto, Toronto, Canada

Background/Rationale or Objectives/Purpose

The increasing adoption of digital screening tools fails to address equity in identifying distress when providing Psychosocial Oncology (PSO) care to demographically vulnerable groups. This study implements a proactive care model to identify vulnerable patients and provide early PSO care.

Methodology or Methods

This implementation science study is being conducted in the oncology clinics at the Princess Margaret Cancer Centre in Toronto, Ontario. Data from the initial two pilot sites, Head and Neck and Gynaecologic Oncology, will be presented. The study participants consist of all new patients attending the pilot sites, healthcare providers and admins.

The Consolidated Framework for Implementation Research (CFIR) was used to guide project development and identify barriers and facilitators. Formative evaluation included individual interviews, structured observations, conversations with stakeholders and staff satisfaction surveys. To assess the results of implementation, a mixed methods approach combining both qualitative and quantitative data will be employed.

Impact on Practice or Results

A total of 23 Oncologists, 4 nurses and 2 patient flow coordinators participated in the interviews. There were eight preliminary themes identified. Facilitators were identified mostly in the CFIR intervention and inner-setting domains and barriers were found in almost all CFIR domains. Plan-Do-Study-Act solutions include refining the vulnerability screening tool with staff input, using iPads onsite to assist with screening, developing tip sheets and tailoring training for staff.

Discussion or Conclusions

The study used CFIR to examine key stakeholder perspectives and workflow experience at the institutional and individual levels to facilitate implementation. Successful implementation will deliver more proactive care models to address equity and enable prompt identification of distress in highly vulnerable oncology patients.

## 11. Final Category: I. Survivorship

13

### 11.1. Translating Research Data into Practice to Improve the Trajectories of AYA Pediatric Brain Tumour Survivors (PBTS) Following Treatment: Preliminary Results of Two Focused Workshops, with Different Stakeholders, in Québec

Marco Bonanno ^1,2^, Claude Julie Bourque ^1,3^, Lye-Ann Robichaud ^2,4^, Charlotte Roy ^2,4^, Ariane Levesque ^2,4^, Sébastien Perrault ^1,3^, Leandra Desjardins ^1,2^ and Serge Sultan ^2,4^

1. CHU Sainte-Justine Research Centre, Montréal, Canada
2. Psycho-Oncology Centre (CPO), Montréal, Canada
3. Department of Pediatrics of Université de Montréal, Montréal, Canada
4. Department of Psychology of Université de Montréal, Montréal, Canada

Background/Rationale or Objectives/Purpose

PBTS may experience social, academic and employment difficulties during aftercare. Despite widely recognized needs, PBTS often do not use services offered to them. This study is a third of a three-step process aiming at implementing solutions that were previously developed and prioritized by PBTS and parents (*n =* 68) in clinical practice.

Methodology or Methods

We organized two online workshops with a convenience sample of professionals (*n =* 15) and decision makers (*n =* 6) from different sectors in Quebec (health, education, non-profit organizations). Before the workshops, we surveyed the participants to identify which of the previously prioritized solutions could be most effectively implemented. Then, we selected the two most promising solutions and conducted two online workshops that lasted 2 h each. The workshop with professionals aimed at identifying the main elements and their concrete translation that would need to be integrated. The workshop with decision makers aimed at identifying institutional strategies for implementing these solutions.

Impact on Practice or Results

The two solutions that were chosen were (1) to provide ongoing evaluation to PBTS and (2) to better help PBTS understand their needs to ask for appropriate services. The participants formulated recommendations: promoting cross-sector involvement in offering services; developing a new role of case manager that would intersect professions; including stakeholders from outside the healthcare sector in integrated intervention plans; and offering systematic and interdisciplinary consultations to better evaluate the assessment needs of PBTS.

Discussion or Conclusions

Future research should focus on pilot testing each of these proposals to improve the quality of life of this vulnerable population in aftercare.

14

### 11.2. Factors That Influence Breast Cancer Survivor Identity

Kevin Gunther ^1^, Richard Wassersug ^2^, Robin Urquhart ^3^ and Chiara Gottheil ^3^

1. University of British Columbia, Victoria, Canada
2. University of British Columbia, Vancouver, Canada
3. Dalhousie University, Halifax, Canada

Background/Rationale or Objectives/Purpose

Not all individuals diagnosed with a cancer are comfortable being labelled a “survivor”. Our objective was to assess how disease state, demographic variables, and emotional well-being correlate with acceptance of “cancer survivor” identity.

Methodology or Methods

We focused on Canadian women treated for breast cancer to give homogeneity to the population in terms of diagnosis and medical management. We used an online questionnaire to survey participants about their comfort with self-identifying as a cancer survivor or being identified by others as a “survivor”. Data were collected from 256 women using validated psychometric questionnaires, such as the PHQ-9, GAD-7, and distress thermometer, plus open-ended questions about what the label “survivor” meant to participants.

Impact on Practice or Results

A total of 62% of respondents rated their comfort with the label “survivor” as 8/10 or higher. Significant positive relationships were found between survivor identity and time since both diagnosis (*p* < 0.0001) and last oncological treatment (*p* = 0.036). Having metastatic or recurrent disease diminished comfort with survivor identity (*p* < 0.0001, *p* = 0.038, respectively). While 35% of participants’ comments were positive about the label “survivor”, repeatedly flagged barriers to acceptance of the label were a dislike of the war-like nature of the term “survivor” and having recurrence or metastatic disease.

Discussion or Conclusions

Several factors correlate with acceptance of survivor identity. However, many individuals are not comfortable being labeled a “survivor” solely because they have been treated for cancer. Cancer support networks and those promoting survivorship care plans need to be aware of the factors that influence comfort with the label “survivor”.

17

### 11.3. A Systematic Review of Models of Follow-Up Care for Survivors of Childhood Cancer: The Role of Policies and Guidelines in Guiding Future Care in Canada

Rachelle Drummond ^1^, Travis Carpenter ^2^ and Fiona Schulte ^1^

1. University of Calgary, Calgary, Canada
2. University of Toronto, Toronto, Canada

Background/Rationale or Objectives/Purpose

Several different models of follow-up care for survivors of childhood cancer exist, leading to disparate outcomes. This study aimed to: (1) review research comparing outcomes across different models of follow-up care, (2) explore policies and guidelines for follow-up care across Canada.

Methodology or Methods

Studies included: (1) survivors of cancer diagnosed < 21 years; (2) >2 years post-treatment and/or 5 years post-diagnosis; (3) a comparison of models on health, quality of life (QOL), or psychosocial outcomes. In addition, we examined existing follow-up policies and guidelines via published studies and a grey literature search of government and health agency websites and documents.

The Cochrane Handbook’s systematic review tool was used. The following databases were searched: MEDLINE(Ovid), EMBASE, PsycINFO, the Cochrane Library, PubMed, CINAHL, and Web of Science. A Canada-wide scan for follow-up guidelines and policies was conducted as a separate search that included the grey literature from provincial governments and health agencies.

Impact on Practice or Results

A total of 2051 articles were identified. One article met the inclusion criteria. In comparison with a community-based follow-up care model, survivors who received follow-up care at a cancer center were more appropriately screened for diagnosis-specific late effects and experienced fewer long-term effects. Substantial gaps in follow-up care policies and guidelines were revealed.

Discussion or Conclusions

The findings of this review indicate that (1) further research is needed comparing models of follow-up care to establish guidelines and policies that will benefit survivors of childhood cancer and (2) a national standard for follow-up care for survivors of childhood cancer must be created to improve the long-term QOL of Survivors.

29

### 11.4. Canadian Cancer Survivors’ Use of Cannabis as a Sleep Aid: The Impact of Cannabinoid Content and Method of Ingestion

Rachel Lee ^1,2^, Mackenzie Lush ^1^, Jennifer Donnan ^1^, Nicholas Harris ^1^ and Sheila Garland ^1,2^

1. Memorial University of Newfoundland, St. John’s, Canada
2. Beatrice Hunter Cancer Research Institute, Halifax, Canada

Background/Rationale or Objectives/Purpose

Cannabis is increasingly used to manage cancer treatment-related symptoms, including sleep. This study investigated whether methods of ingestion or cannabinoid content differ in their sleep effects.

Methodology or Methods

Adult Canadian cancer survivors (*n =* 1464) completed an online survey via the Angus Reid Institute that included the Insomnia Severity Index and questions about cannabis use for the purpose of sleep. A series of chi-squared analyses were performed.

Impact on Practice or Results

A total of 23% currently use cannabis for the purpose of sleep (*n =* 344; Mage = 61.1; 50% women; 91% white). The most common methods of ingestion were edibles (20%), smoking (18%), and oils/sprays (16%). Products contained either mostly THC (36%), balanced amounts of CBD/THC (33%), or mostly CBD (23%). There was no effect of the method of ingestion nor cannabinoid content on insomnia. However, of those reporting that cannabis helped them relax, more reported smoking cannabis (*p* = 0.015), and less reported using cannabis with mostly CBD (*p* = 0.008). Of those that felt that cannabis helps them sleep through the night, fewer smoked cannabis compared to other forms (*p* = 0.04). Of those that said cannabis helps them fall asleep faster, more smoked or used multiple methods of ingestion (*p* = 0.004) and used cannabis containing balanced CBD/THC (*p* = 0.02).

Discussion or Conclusions

One in four Canadian cancer survivors regularly use cannabis to improve their sleep. Not all cannabis is perceived to impact sleep in the same way. More research is needed to examine the actual efficacy of cannabis as a sleep aid.

37

### 11.5. Change in Objective Cognitive Function in Cancer Survivors with Insomnia and Perceived Cognitive Impairments Receiving Virtual Cognitive Behavioural Therapy for Insomnia

Joshua Tulk ^1^, Joshua Rash ^1^, Joseé Savard ^2^, Melanie Seal ^3^, John Thoms ^3,4^, Robin Urquhart ^4,5^, Sondria Browne ^1^ and Sheila Garland ^1,3^

1. Department of Psychology, Faculty of Science, Memorial University, St. John’s, NL, Canada
2. School of Psychology, Faculty of Social Sciences, Université Laval, Québec, QC, Canada
3. Discipline of Oncology, Faculty of Medicine, Memorial University, St. John’s, NL, Canada
4. Beatrice Hunter Cancer Research Institute, Halifax, NS, Canada
5. Department of Community Health and Epidemiology, Faculty of Medicine, Dalhousie University, Halifax, NS, Canada

Background/Rationale or Objectives/Purpose

Cognitive behavioural therapy for insomnia (CBT-I) can improve perceived cognitive functioning among cancer survivors with insomnia; however, little is known about its impact on objective cognitive functioning.

Methodology or Methods

Thirty Atlantic Canadian cancer survivors with insomnia and objective cognitive impairments (i.e., two neuropsychological tests 1.5 SD or one test 2.0 SD below mean at baseline) who received CBT-I were compared to a waitlist control group as part of an ongoing randomized controlled trial. Participants completed the Controlled Oral Word Association Test, Hopkins Verbal Learning Test–Revised, and Digit Span at baseline, two months (waitlist controls only), and post-treatment. Z-scores and reliable change indices were calculated for each test. A series of 2-by-2 ANCOVAs assessed the impact of CBT-I on cognition after statistically adjusting age, education, and baseline performance. Fisher exact tests were used to explore differences in demographic and clinical characteristics among participants reporting significant improvement in cognitive function.

Impact on Practice or Results

Participants were a mean of 61 years with 15 years of education. Breast cancer was the most common cancer (63%). There were significant improvements in word recall [*p* = 0.02, *η*_p_^2^ = 0.22], retention [*p* = 0.01, *η*_p_^2^ = 0.27], and recognition [*p* = 0.05, *η*_p_^2^ = 0.15], in the CBT-I-treated group compared to baseline and waitlist controls. Participants with significant improvement (*n* = 18) were more likely to be older than 60 years (*V* = 0.35), have high school or less education (*V* = 0.21), and deny pre-treatment depression (*V* = 0.26) relative to participants without improvements.

Discussion or Conclusions

CBT-I is the gold standard for treating insomnia. Early results suggest that it can also improve select domains of objective cognitive function.

48

### 11.6. Fear of Cancer Recurrence in Breast Cancer Survivors Carrying a BRCA1/2 Mutation: Findings of a Mixed Methods Study

Josée Savard ^1,2^, Alexandra Michel ^1,3^, Catherine Filion ^2,3^, Aude Caplette-Gingras ^2,4^, Jocelyne Chiquette ^2,5^ and Michel Dorval ^6,7^

1. Université Laval, School of Psychology, Québec, Canada
2. Centre de recherche du CHU de Québec-Université Laval, Québec, Canada
3. Université Laval Cancer Research Center, Québec, Canada
4. Centre des maladies du sein, CHU de Québec-Université Laval, Québec, Canada
5. Université Laval, Faculty of Medicine, Québec, Canada
6. Université Laval, Faculty of Pharmacy, Québec, Canada
7. CISSS de Chaudière-Appalaches Research Center, Lévis, Canada

Background/Rationale or Objectives/Purpose

Objectives/purpose. To assess the severity of fear of cancer recurrence (FCR) and understand how FCR is experienced in women treated for breast cancer and carrying a BRCA1/2 mutation.

Methodology or Methods

Methods. Participants having completed treatment for non-metastatic breast cancer and carrying a BRCA1/2 mutation were recruited through the Réseau ROSE mailing list. For the quantitative study, women completed a battery of questionnaires online that included the Fear of Cancer Recurrence Inventory-severity (FCRI-S) subscale. Then, another e-mail was sent to the 89 responders to recruit participants for the qualitative study. Three focus groups were conducted (*n* = 19).

Impact on Practice or Results

Results. The mean FCRI-S score was 16.8, which exceeds the clinical cut-off score of 13, and 70.8% of the participants showed a clinical level of FCR. Qualitative analyses indicated that FCR was an unmet need in these women. While all women who underwent a preventive surgery (e.g., bilateral mastectomy) reported a significant decrease in their FCR soon after the surgical procedure(s), many experienced residual FCR thereafter. Participants were unanimous in affirming the necessity to develop FCR interventions that are specific to the needs of BRCA1/2 mutation carriers. Group interventions were particularly emphasized.

Discussion or Conclusions

Conclusion and clinical implications. These results suggest that, although being the most effective medical option to reduce the actual risk of local recurrence (or second breast cancer), prophylactic surgery is not sufficient to reduce FCR. A psychological intervention targeting specifically FCR would be an appropriate complement to preventive surgery.

56

### 11.7. Multi-Stakeholder Perspectives on Transition of Care of AYA Survivors of Hematological Cancers

Katrin Julia Kaal ^1,2^, Sarah Scruton ^1^, Stephanie Villeneuve ^1,3^, Amy Trottier ^1,2^, Annette Flanders ^3^, Roetka Gradstein ^1^, Chris Lewis ^4^, Allie Campbell ^4^, Carol Digout ^5^ and Robin Urquhart ^1^

1. Dalhousie University, Halifax, Canada
2. Nova Scotia Health, Halifax, Canada
3. IWK Health Center, Halifax, Canada
4. Patient Partner, Halifax, Canada
5. Atlantic Provinces Pediatric Hematology Oncology Network, Halifax, Canada

Background/Rationale or Objectives/Purpose

Adolescent and young adult (AYA) cancer survivors have unique needs. A particularly vulnerable time in the lives of these patients is during the transition of care from surveillance with hematology/oncology to long-term survivorship with primary care providers. Evidence of outcomes of transitional care services and knowledge about what a successful transition of care entails is scarce. The objective of the current project is to develop a robust definition of ‘successful transition of care’ from a multi-stakeholder perspective (i.e., within and across stakeholder groups), and work towards consensus of definition across groups.

Methodology or Methods

Recruitment of stakeholder groups across NS, NB and PEI include AYA cancer survivors, caregivers, pediatric and adult hematologists/oncologists, and primary care providers. Stakeholder focus groups are being conducted using the Nominal Group Technique, an approach to group decision-making that places weight on all participants having an equal opportunity to express a view. Focus group recruitment and data collection are ongoing.

Impact on Practice or Results

The first focus group of adult hematologists revealed factors pertaining to the current state of the health system (i.e., inadequate access to primary care) as well as an overarching theme of communication relating to patient issues (i.e., life goals, ongoing issues) and provider-to-provider exchange/flow of information as important factors when guiding patients through this transition in their follow-up care.

Discussion or Conclusions

Defining what constitutes successful transition of care for AYA cancer survivors will provide a better understanding of what is needed to optimize transitions of care services and improve transition outcomes.

61

### 11.8. The Relationships between Perfectionism and Cancer-Related Symptoms in Non-Metastatic Breast Cancer Patients Undergoing Chemotherapy

Véronique Massicotte ^1,2^, Aude Caplette-Gingras ^2,3^, Frédéric Langlois ^4^, Jean-Philippe Gouin ^5^, Gordon Flett ^6^, Danielle Molnar ^7^, Julie Lemieux ^2,3^ and Josée Savard ^1,2^

1. School of Psychology, Université Laval, Québec, Canada
2. CHU de Québec-Université Laval Research Center, Oncology division, Québec, Canada
3. Centre des maladies du sein, CHU de Québec-Université Laval, Québec, Canada
4. Department of Psychology, Université du Québec à Trois-Rivières, Trois-Rivières, Canada
5. Department of Psychology, Concordia University, Montréal, Canada
6. Faculty of Health, York University, Toronto, Canada
7. Department of Child and Youth Studies, Brock University, St-Catharines, Canada

Background/Rationale or Objectives/Purpose

There is some evidence suggesting that perfectionism could negatively affect patients’ adjustment to chronic disease. However, few studies have investigated its role in oncology. This study aimed to examine cross-sectional and prospective relationships between two dimensions of perfectionism (perfectionistic strivings (PS); perfectionistic concerns (PC)) and psychological (anxiety, depression, fear of cancer recurrence (FCR)) and psychophysiological symptoms (insomnia, fatigue, pain, cognitive impairments, sexual dysfunctions).

Methodology or Methods

Forty women diagnosed with non-metastatic breast cancer were recruited before their chemotherapy at the Hôpital du Saint-Sacrement (CHU de Québec-Université Laval) in Quebec City between July 2019 and March 2020. Participants completed self-reported questionnaires before (T1), at mid-treatment (T2), after chemotherapy completion (T3), and six months later (T4).

Impact on Practice or Results

Results of Spearman correlations revealed significant associations between perfectionism dimensions and greater anxiety, depression, and FCR. These relationships were consistent across time points (T1 to T4). Some significant correlations were also observed between perfectionism and psychophysiological symptoms. Results of linear repeated measures models including participant’s random effect showed that PS were significantly associated with higher levels of anxiety, *F*(1, 131) = 6.01, *p* = 0.016, while PC significantly predicted greater depressive symptoms, *F*(1, 94.2) = 5.81, *p* = 0.018. No significant prospective association was found between perfectionism and FCR and psychophysiological symptoms (*p*s = 0.084 to 0.922).

Discussion or Conclusions

Findings support the relevance of assessing the role of perfectionism in cancer patients and, if replicated, suggest that patients with higher levels of perfectionism could be more vulnerable to experiencing psychological distress.

68

### 11.9. ‘My Mind Just Feels Slowed Down’: Fatigue as a Contributor to Perceived Cognitive Impairment in Cancer Survivors

Krista Greeley, Joshua Tulk and Sheila Garland

Memorial University of Newfoundland, St. John’s, Canada

Background/Rationale or Objectives/Purpose

Roughly 50% of cancer survivors experience fatigue related to their cancer diagnosis or treatment. Fatigue often co-occurs with other issues such as insomnia, anxiety, and depression. Fatigue is also associated with perceived cognitive impairment (PCI). This study examined the relative associations between fatigue, insomnia, mood disturbance, and PCI.

Methodology or Methods

A sample of 132 Atlantic Canadian cancer survivors (77% women) with insomnia disorder and PCI symptoms were recruited as part of a larger randomized controlled trial. This analysis used the following baseline measures: the Functional Assessment of Cancer Therapy-Cognitive Function, the Multidimensional Fatigue System Inventory (Short Form), the Insomnia Severity Index, and the Hospital Anxiety and Depression Scale. Univariate and multivariate regressions explored factors associated with PCI, adjusting for age and education.

Impact on Practice or Results

On average, participants were 60 years old with 16 years of education. The most common cancer type was breast cancer (45%). On average, they completed their cancer treatment 82 months (approximately 7 years) prior to the study. At the univariate level only, fatigue (*R*^2^ = 0.22, *p* < 0.001) and insomnia (*R*^2^ = 0.12, *p* < 0.001) were associated with PCI. Only fatigue remained significantly associated with PCI in the multivariate model (*R*^2^ = 0.22, *p* < 0.001). Of the five fatigue subscales (physical, emotional, mental, general, and vigour), only mental fatigue was significantly associated with PCI, *R*^2^ = 0.43. *p* < 0.001.

Discussion or Conclusions

Fatigue, particularly mental fatigue, has stronger associations with PCI than insomnia, anxiety, and depression. Interventions that treat fatigue in cancer survivors may also help improve PCI symptoms.

105

### 11.10. “There’s No Such Thing as Good Cancer”: A Qualitative Exploration of the Experiences of Young Adult Survivors of Thyroid Cancer

Sara Cho, Perri Tutelman, Tessa Chomistek, Iqra Rahamatullah, Charlotte Ryder-Burbidge, Miranda Fidler-Benaoudia and Fiona Schulte

University of Calgary, Calgary, Canada

Background/Rationale or Objectives/Purpose

The incidence of thyroid cancer in young adults (YAs) (diagnosed between 19 and 45 years) has increased exponentially in recent decades. At the same time, there is growing concern surrounding the overdiagnosis of indolent thyroid cancer, leading to invasive and potentially unnecessary interventions that can significantly impact young patients’ lives. However, the experiences of survivors of thyroid cancer have been largely understudied. The purpose of this study was to explore the experiences of YA survivors of thyroid cancer.

Methodology or Methods

The qualitative research design of hermeneutic phenomenology guided this study. Participants completed a demographic survey and semi-structured interview that was subsequently transcribed verbatim and analyzed using reflexive thematic analysis.

Impact on Practice or Results

Thirty-six YA survivors of thyroid cancer (79% female, median age: 43 years, median age at diagnosis: 38 years) participated. Participants’ experiences were characterized by two themes: (1) reconciling the meaning of the ‘c’ word (cancer) as a dangerous and life-threatening diagnosis with lived experience of thyroid cancer; (2) thyroid cancer leaves patients with lifelong physical and emotional scars.

Discussion or Conclusions

Survival of thyroid cancer is characterized by both physical and emotional challenges. However, this experience is often undermined by how the diagnosis is made, perceived, and managed (e.g., disjointed care pathways). Given concerns around overdiagnosis, results highlight the need for better education (e.g., risks and benefits of treatment) and potential to change how thyroid cancer is framed (e.g., cancer as a chronic illness, cancer exists on a spectrum). Findings highlight the need for tailored psychosocial supports for this growing population.

107

### 11.11. Adherence to Follow-Up Care among Adolescent and Young Adult Survivors of Childhood Cancer in Canada

Brianna Henry ^1,2^, Sharon Hou ^1,3^, Rachelle Drummond ^1^, Kyle Canton ^2^, Caitlin Forbes ^1^, Kathleen Reynolds ^1,2^ and Fiona Schulte ^1,2^

1. University of Calgary, Calgary, Canada
2. Alberta Children’s Hospital, Calgary, Canada
3. British Columbia Children’s Hospital, Vancouver, Canada

Background/Rationale or Objectives/Purpose

Survivors of childhood cancer require long-term follow-up (LTFU) for surveillance of treatment-related effects. Yet, many survivors do not adhere to their LTFU care. This study (1) described rates of adherence to LTFU care among adolescent and young adult (AYA) survivors in Canada; (2) examined the relationships of post-traumatic stress symptoms (PTSS) and health locus of control (HLOC) to adherence; and (3) explored predictors of adherence.

Methodology or Methods

AYA survivors with a cancer diagnosis <18 years, currently 18–39 years, 2 years post-treatment or 5 years post-diagnosis, and living in Canada. Survivors (*n* = 106; 68.6% female; *M_Age_* = 28.41 years, *SD* = 5.22) were recruited through LTFU clinics and community settings to complete an online survey using standardized measures of PTSS and HLOC. Adherence was operationalized as having received cancer-specific care in the past two years.

Impact on Practice or Results

Eighty-one percent of AYAs adhered to LTFU care. AYAs who adhered demonstrated greater “powerful others HLOC” than AYAs who did not, *t*(100) = −1.98, *p* = 0.050, *d* = 0.53. Greater “powerful others HLOC” predicted adherence, AOR, 1.22; 95%CI, 1.03–1.45. AYAs who adhered reported greater levels of PTSS than AYAs who did not, *t*(100) = −2.57, *p* = 0.012, *d* = 0.72., and greater levels on re-experiencing (*t*(100) = −3.04, *p* = 0.003, *d* = 0.90), alterations in cognition/ mood (*t*(100) = −2.03, *p* = 0.046, *d* = 0.58), and hyper-arousal (*t*(100) = −2.33, *p* = 0.022, *d* = 0.63) domains. Greater levels of re-experiencing predicted adherence (AOR, 1.42; 95%CI, 1.06–1.91).

Discussion or Conclusions

HLOC and PTSS influence adherence to LTFU care among AYA survivors. Provision of care must encompass integrative services Additional modifiable factors must be considered in developing interventions.

## 12. Final Category: J. Palliative and End-of-Life Care

27

### 12.1. Medical Assistance in Dying in Canada and Suffering: A Multi-Phase Study to Improve MAiD Education, Practice Guidelines and Plan a Research Agenda

Melissa Henry ^1^, James Downar ^2^, Madeline Li ^3^, Brian Michara ^4^, Gauthier Lynn ^5^, Marcoux Isabelle ^2^, Alias Ali ^1^, Bisson-Gervais Vanessa ^1^, Jun Liu ^1^ and Albert Justine ^1^

1. McGill University, Montreal, Canada
2. University of Ottawa, Ottawa, Canada
3. University of Toronto, Toronto, Canada
4. University of Quebec in Montreal, Montreal, Canada
5. University Laval, Quebec, Canada

Background/Rationale or Objectives/Purpose

Medical Assistance in Dying (MAiD) is legal in Canada since June 2016. According to the Annual Report on MAiD in Canada, requests are mostly motivated by a combination of psychological, existential and social issues. While the psycho-oncology field offers several interventions to relieve suffering, it is unclear how issues of suffering are addressed in practice.

Methodology or Methods

The current study takes place in three phases. Phase (1): A scoping review conducted to better understand how the Canadian scientific and grey literature defines, assesses, and manages suffering within the practice of MAiD. The review uncovered lacunas in MAiD practice guidelines regarding the management of patient suffering. Phase (2): A Canadian Institutes of Health Research (CIHR) 2-day pan-Canadian virtual Knowledge Dissemination and Research Planning Initiative on 14–15 July 2022, to disseminate results of our scoping review to stakeholders, discuss challenges in how MAiD eligibility criteria are currently defined and evaluated in clinical practice documents, and develop a research agenda on MAiD in Canada that focuses on suffering.

Impact on Practice or Results

Phase (3): Based on the items generated at the planning initiative, a Delphi study will be completed of the clinical practice document changes and research priorities on MAiD in Canada and a survey of clinical practices around MAiD and educational needs of MAiD assessors and providers.

Discussion or Conclusions

Overall, the findings of the study will be featured in a report, which will be distributed largely as part of our knowledge dissemination strategy. The findings will give direction on MAiD practice and research.

45

### 12.2. Evidence to Integrate Supportive Care for Adolescent and Young Adults with Advanced Cancer: The Perspectives of Cancer Care Providers and Decision Makers

Jonathan Avery ^1,2^, Kristin Campbell ^3^, Pamela Mosher ^1^, Ahmed Al-Awamer ^1^, Karen Goddard ^4^, Annemarie Edwards ^5^, Laura Burnett ^5^, Breffni Hannon ^1^, Abha Gupta ^1^ and Fuchsia Howard ^3^

1. Princess Margaret Cancer Centre, Toronto, Canada
2. Royal Roads University, Victoria, Canada
3. University of British Columbia, Vancouver, Canada
4. BC Cancer, Vancouver, Canada
5. Canadian Cancer Society, Toronto, Canada

Background/Rationale or Objectives/Purpose

Objective/purpose: Adolescents and young adults (AYAs), defined as individuals aged 15 to 39 years of age, face unique challenges when diagnosed with an advanced cancer and could greatly benefit from supportive or palliative care. However, successful integration lags far behind care programming in pediatric and older age groups. The study purpose was to explore the barriers and facilitators to the early integration of supportive and palliative care for AYAs with advanced cancer.

Methodology or Methods

Methods: Using a qualitative interpretive descriptive approach, one-on-one semi-structured virtual interviews were conducted with cancer care providers, program directors and administrators from across Canada who support AYAs with advanced cancer. Data were analyzed using the constant comparative method.

Impact on Practice or Results

Results: The sample consisted of 24 individuals (6 oncologists, 5 nurses, 3 social workers, 1 palliative care physician, 2 directors, and 7 allied health professionals). Overall, the participants described struggling to support AYAs with advanced cancer because they felt social pressure to convey messages of hope for achieving a state of no evidence of disease even when the illness trajectory dictated otherwise. To facilitate the integration of supportive and palliative care, the participants suggested communication skills training to learn how to balance messages of hope with prognostic realities; incorporating a team-based approach to share responsibilities and difficulties; and creating clearer referral care pathways to supportive/palliative care programs.

Discussion or Conclusions

Conclusions: Results from this study are informative to better equip professionals to support AYAs and will guide co-design workshops to develop an implementation strategy to integrate improved care for this population.

46

### 12.3. End-of-Life Symptom Burden among Patients with Cancer who Pass Away with and without Medical Assistance in Dying: A Propensity Score-Matched Cohort Study

K Brooke Russell ^1^, Caitlin Forbes ^1^, Siwei Qi ^2^, Claire Link ^2^, Linda Watson ^1,2^, Andrea DeIure ^2^, Shuang Lu ^2^, James Silvius ^1,2^, Brian Kelly ^1,3^, Barry D Bultz ^1,3^ and Fiona Schulte ^1,2^

1. University of Calgary, Calgary, Canada
2. Alberta Health Services, Calgary, Canada
3. University of Newcastle, Newcastle, Australia

Background/Rationale or Objectives/Purpose

Cancer is the most common medical condition among Canadians who receive medical assistance in dying (MAiD). Little is known about the symptom burden experienced by cancer patients who receive MAiD. We examined longitudinal symptom data in the last year of life among cancer patients in Alberta, comparing those who were provided with MAiD to those who died without MAiD.

Methodology or Methods

We conducted a retrospective analysis of routinely collected patient-reported outcomes, using data from the Edmonton Symptom Assessment System-Revised (ESAS-r), for patients with cancer who died between July 2017 and January 2019. Propensity-score matched cohorts of those who received MAiD (*n* = 149) and those who did not (non-MAiD, *n* = 149) were compared using ESAS-r data over the final 12 months of life. Mixed-effect models were used to evaluate symptom differences between cohorts over time.

Impact on Practice or Results

Both cohorts experienced increased severity in all ESAS-r symptoms over time (β = 0.086 to 0.231, *p* = 0.002 to <0.001). Non-MAiD cohort patients reported significantly lower anxiety (β = −0.831, *p* = 0.044) and lack of appetite (β = −0.934, *p* = 0.039) overall, compared to those in the MAiD cohort.

Discussion or Conclusions

All patients experienced increasing symptom severity in the year prior to death. Those who ultimately received MAiD reported greater anxiety and lack of appetite overall, with notable differences at 12 months prior to death, before most requests for MAiD had been submitted. These results highlight an opportunity to address patient suffering through routine collection of patient-reported outcomes, focused clinical assessment, and interventions targeting specific symptoms.

62

### 12.4. How COVID-19 Restrictions in Hospital Environments Impact Communication with Healthcare Teams at the End of Life: A Qualitative Study

Runqun Helen Zheng ^1^, Yun-Ting Paige Chu ^2^, Alison Stere ^2^, Sally Bean ^3^, Elie Isenberg-Grzeda ^3^, Debbie Selby ^3^, Madeline Li ^2^, Breffni Hannon ^2^, Sarah Hales ^2^ and Rinat Nissim ^2^

1. University of Toronto, Toronto, Canada
2. University Health Network, Toronto, Canada
3. Sunnybrook Health Sciences Center, Toronto, Canada

Background/Rationale or Objectives/Purpose

The COVID-19 pandemic and institutional control measures have changed how people die and how their families grieve. As part of an ongoing mixed-method study aimed at evaluating the quality of death outcomes during the COVID-19 pandemic, as well as the bereavement outcomes of family caregivers and the impact of pandemic control measures on these outcomes, we conducted a qualitative grounded theory analysis. Specifically, we examined how COVID-19 restrictions in hospital environments impact communication between healthcare teams and family caregivers during end-of-life hospital care.

Methodology or Methods

Retrospective lists of patient deaths in two large urban hospitals in Toronto, Ontario, Canada were used to identify bereaved family members for approach at least six months following patient death to complete a questionnaire package. Purposive sampling was used to select a subset of participants for semi-structured interviews about their experiences of patient death and bereavement. A grounded theory method informed the analysis of transcripts.

Impact on Practice or Results

Ten transcripts were selected for qualitative analysis. The analysis identified four major categories related to caregiver communication with healthcare teams during the pandemic: lack of consistency; lack of transparency; lack of accessible communication; and extra advocacy needed for palliative care. These categories showcase the unique challenges being exacerbated or newly created due to the pandemic and its associated restrictions.

Discussion or Conclusions

The results of this study offer important insights into end-of-life care in the context of a pandemic. These findings can inform the development of policy and intervention strategies for post-COVID-19 and future pandemics.

65

### 12.5. Experiences of Informal Caregivers Regarding MAiD in Canada: A Narrative Review

Justine Albert ^1^ and Carmen Loiselle ^1,2^

1. McGill University, Montreal, Canada
2. Jewish General Hospital, Montreal, Canada

Background/Rationale or Objectives/Purpose

Informal caregivers play an important role in end-of-life (EOL) care by helping patients meet their care needs and facilitating decision-making. Medical Assistance in Dying (MAiD) may reinforce this role as caregivers support the patient through the MAiD process. Therefore, it is imperative that the caregiver perspective is reflected in MAiD guidelines and interventions. The review aims to identify and describe the evidence of informal caregiver experiences related to MAiD within Canada.

Methodology or Methods

A narrative review was conducted by searching MEDLINE, CINAHL, EMBASE, PSYCHINFO, and the grey literature. The studies included were published between June 2016 and November 2022. This review included qualitative and quantitative studies (*n =* 15) about informal caregivers exposed to a MAiD experience in Canada. All studies were coded in NVivo and thematic analysis was used to identify caregiver barriers and facilitators. Key search terms included: Caregivers, MAiD, and Canada.

Impact on Practice or Results

Of the 132 articles reviewed, 15 studies were retained. These studies addressed the following areas: (1) Patient–family EOL experiences including themes of fulfilling patient wishes, involvement in decision-making and the caregiver’s relationship with the patient. (2) Family dynamics during and after the death of the significant other specifically incorporating cultural death practices and the impact of stigma. (3) Healthcare system interactions included communication with the healthcare team and navigating MAiD logistics.

Discussion or Conclusions

Study findings highlight the importance of informal caregivers’ experiences throughout MAiD-related processes. MAiD-related resources for caregivers are desperately needed and these must be co-developed with them to ensure person-centred care through end-of-life and bereavement periods.

87

### 12.6. Mortality-Focused Language of Palliative Care Descriptions and Clinician Willingness to Recommend the Service

Sarah Alonzi, Makaela Yamoah, Derek Dell’Angelica, Sobia Sayeed, Troy Coaston, Citlali Perez and Annette L. Stanton

University of California, Los Angeles, Los Angeles, USA

Background/Rationale or Objectives/Purpose

Palliative care improves quality of life for patients with serious illness, yet eligible patients underutilize it due to conflation with end-of-life care. This study aimed to determine whether mortality salience (i.e., the ability to bring about thoughts of death) influences medical trainees’ perceptions of palliative care.

Methodology or Methods

Medical students (*n =* 95; M age = 25.9 years; 60% women) completed a web-based experiment. Participants were assigned randomly to read either a high- or low-mortality salience palliative care description, which included a palliative care definition adapted from the National Cancer Institute’s Dictionary of Cancer Terms. Participants then rated on Likert-type scales the extent to which they believed the description suggested thoughts of death and whether they would recommend palliative care to patients with a chronic or terminal illness. Analyses examined whether palliative care descriptions influenced perceptions of the service using Welch’s independent samples *t*-tests.

Impact on Practice or Results

Participants perceived the “high mortality salience” condition to be more evocative of thoughts of death than the “low mortality salience” condition t(87.3) = −2.23, *p* = 0.028, but they did not differ significantly in willingness to recommend palliative care to a patient with a chronic (*p* = 0.67) or terminal (*p* = 0.39) illness. Participants were more likely to recommend palliative care to a patient with a terminal illness than a chronic illness t(94) = 8.50, *p* < 0.001.

Discussion or Conclusions

The finding that medical trainees are more likely to recommend palliative care to people with terminal (vs. chronic) illness suggests that early education is needed on the benefits of early palliative care.

129

### 12.7. Psychosocial Profile of Patients Considering Medical Assistance in Dying (MAiD)

Stefan Aguiar ^1,2^, Aliza Panjwani ^1,2^, Anne Barbeau ^1^, Anne Rydall ^1^, Emma Hagopian ^1^, Gary Rodin ^1,2,3^, Gilla Shapiro ^1,2,3^, Olivia Mazzurco ^1,2^, Roberta Y. Klein ^1^ and Madeline Li ^1,2^

1. Princess Margaret Cancer Centre, University Health Network, Toronto, Canada
2. University of Toronto, Toronto, Canada
3. Global Institute of Psychosocial, Palliative and End-of-Life Care (GIPPEC), University of Toronto and Princess Margaret Cancer Centre, Toronto, Canada

Background/Rationale or Objectives/Purpose

Emotional and cognitive factors, such as depressive symptoms, death anxiety and experiential avoidance, may be associated with attitudes and decisions about medical assistance in dying (MAiD) among patients with advanced cancer. We aim to characterize patients at different stages of decision making about MAiD (i.e., those who are seeking information (SI), those who have requested MAiD (R), and those who have decided not to request MAiD (NR)) on a number of clinically relevant constructs.

Methodology or Methods

This preliminary, cross-sectional analysis of baseline data is part of a larger longitudinal study of the attitudes and predictors of MAiD in individuals with advanced cancer. We present descriptive data on 42 patients (SI = 6, R = 3, NR = 33) on depressive symptoms (Patient Health Questionnaire-9 (PHQ-9)); death anxiety (Death and Dying Distress Scale (DADDS)); experiential avoidance (Brief Experiential Avoidance Questionnaire (BEAQ)); intolerance of uncertainty (Intolerance of Uncertainty Scale-12 (IUS-12)); and self-perceived burden (SPB).

Impact on Practice or Results

Average scores among groups on emotional and cognitive variables are as follows: PHQ-9: SI = 10.3, R = 10.3, NR = 6.5; DADDS: SI = 26.5, R =43.3, NR = 18.7; BEAQ: SI = 51.7, R = 48.3, NR = 44.5; IUS-12: SI = 29.5, R = 32.3, NR = 27.3; SPB: SI = 19.3, R = 31.3, NR = 19.4.

Discussion or Conclusions

Patients who requested MAiD appear to show a trend toward higher depressive symptoms, death anxiety, and self-perceived burden at baseline than those who have decided not to request it. Patients seeking information about MAiD have comparable scores to those who requested MAiD on depressive symptoms and experiential avoidance. Findings may assist healthcare providers in identifying mental health vulnerabilities in patients considering MAiD.

## 13. Final Category: K. Primary, Secondary and Tertiary Cancer Prevention

31

### 13.1. Vaping-Specific Regulations as Primary Interventions for Nicotine-Related Cancers: A Scoping Review of Policy at the Federal (Canada), Provincial (British Columbia), and Regional (Okanagan) Levels

Ramona Sharma, Sarah Dow-Fleisner and Laura Struik

University of British Columbia, Okanagan Campus, Kelowna, Canada

Background/Rationale or Objectives/Purpose

Governments and regulatory bodies across Canada have authorized levels of policy measures to regulate e-cigarette visibility, distribution, and usage among youth. Evaluating policies is essential in identifying and remediating factors contributing to youth vaping. This study takes a comparative focus on policies related to youth (aged ≤18) vaping at three levels: federally across Canada; provincially in British Columbia; and regionally within the Okanagan. Objectives include: (1) summarize policy efforts at each level; (2) identify implications of policy targets; and (3) offer insights for policy implementation, awareness, and evaluation at school and regional levels.

Methodology or Methods

A scoping review was conducted using the Arskey and O’Malley framework. Terms like “vaping policies Canada” were searched on Google to compile policies surrounding vaping by the Government of Canada, the Province of British Columbia, and Okanagan schools, health authorities, and municipalities.

Impact on Practice or Results

Data were extracted from 23 policies. Regional policies (*n* = 16) primarily addressed consumption and were found to vary significantly, be reprimand-focused, and be less comprehensive than federal (*n* = 3) and provincial policies (*n* = 4), which addressed production and sale, respectively. School policies differed considerably in addressing possession, distribution, inclusion of awareness campaigns, and knowledge translation of policy content and enforcement.

Discussion or Conclusions

Current policies are modeled after existing smoking policies; few centers allow vaping. Findings suggest streamlining regional and school-based policies to provide youth with a singular comprehensive message that explicitly highlights vaping. Findings also support the identification of disparities in policy awareness at the production, sale, and consumption levels, allowing for improved efficacy of policies as primary interventions.

60

### 13.2. What Are the Challenges of HPV Testing Implementation in the Prevention of Cervical Cancer? Development and Validation of HPV Testing and Self-Sampling Attitudes and Beliefs Scales

Zeev Rosberger ^1,2^, Ovidiu Tatar ^1,3^, Ben Haward ^1^, Patricia Zhu ^1^, Gabrielle Griffin-Mathieu ^1^, Samara Perez ^1,2^, Emily McBride ^4^, Aisha Lofters ^5^, Laurie Smith ^6^, Marie-Hélène Mayrand ^3^, Ellen Daley ^7^ and Julia Brotherton ^8^

1. Lady Davis Institute for Medical Research, Montreal, Canada
2. McGill University, Montreal, Canada
3. University of Montreal, Montreal, Canada
4. King’s College London, London, UK
5. University of Toronto, Toronto, Canada
6. BC Cancer Agency, Vancouver, Canada
7. University of South Florida, Tampa, USA
8. University of Melbourne, Melbourne, Australia

Background/Rationale or Objectives/Purpose

Attitudes and beliefs are key predictors of health behaviour and are critically implicated in the success of HPV testing and self-sampling implementation. Disruptions to screening changes in several countries can be attributed to failure to adequately address concerns about updated testing methods and protocols. In this study, we aimed to develop and validate two scales examining attitudes and beliefs toward HPV testing and HPV self-sampling.

Methodology or Methods

In October–November 2021, a nationally representative sample of cervical screening-eligible Canadians (*n =* 1027) participated in a web-based survey. We included 44 items related to HPV testing and 13 items related to HPV self-sampling attitudes and beliefs based on a systematic literature review. Comprehension was tested in cognitive interviews. The dataset was randomly divided into two equal subsets. On one subset, we identified the optimal number of factors using exploratory factor analysis and parallel analysis. We used Item Response Theory to select the most relevant items in each subscale. Confirmatory factor analysis was used on the remaining dataset to assess model fit.

Impact on Practice or Results

The HPV Testing Attitudes and Beliefs Scale (HTABS) had four factors, and in total twenty items were retained after item reduction. The HPV Self-sampling Attitudes and Beliefs Scale (HSABS) had two factors and seven items were retained. CFA showed good model fit for both final scales.

Discussion or Conclusions

The developed scales fill an important research gap related to measures of HPV test and self-sampling attitudes and beliefs that can inform interventions to increase the acceptability of HPV-based cervical screening.

70

### 13.3. The Behavior Change Techniques Used in Canadian Online Smoking Cessation Programs: Content Analysis

Danielle Rodberg, Laura Struik and Ramona Sharma

University of British Columbia, Kelowna, Canada

Background/Rationale or Objectives/Purpose

Working towards Canada’s federal tobacco strategy of reducing tobacco use to less than 5% by 2035, it is paramount that Canadians have access to effective cessation services. To do so, understanding the behaviour change techniques (BCTs) these websites utilize to both encourage cessation and help individuals achieve their cessation goals is critical to aid Canadians toward tobacco-free lives. This study identifies what techniques websites incorporate when promoting behaviour change and how these techniques are exhibited.

Methodology or Methods

This study includes 12 government-funded websites across Canada including 10 websites funded by provincial or territorial governments and 2 websites funded at the national level. Using the behaviour change techniques outlined in the BCTTv1 taxonomy, websites were coded using deductive content analysis. After completing a training program, the websites were analysed to identify which of the 16 BCT categories and 93 BCTs included in the framework were displayed on the websites.

Impact on Practice or Results

Techniques were included from 14 of the BCT categories with 4 categories employed by all of the websites in this study: goals and planning, social support, natural consequences, and regulation. Various design strategies were taken by programmers to implement these techniques.

Discussion or Conclusions

These results provide programmers with the evidence needed to make improvements that will encourage user engagement and provide effective services. Additionally, since patients are referred to government-funded cessation services when cessation support is needed to accompany their treatment plan ensuring these services will yield positive outcomes is crucial for comprehensive health care.

74

### 13.4. Preparedness for HPV-test Implementation in Cervical Cancer Screening: A Population-Wide Investigation of The Knowledge, Attitudes, and Beliefs of Canadians with Varied Screening Histories

Zeev Rosberger ^1,2^, Ben Haward ^1^, Ovidiu Tatar ^1,3^, Patricia Zhu ^1^, Gabrielle Griffin-Mathieu ^1^ and Samara Perez ^1,2^

1. Lady Davis Institute for Medical Research, Montreal, Canada
2. McGill University, Montreal, Canada
3. University of Montreal, Montreal, Canada

Background/Rationale or Objectives/Purpose

We examined screening-eligible Canadians’ attitudes towards and knowledge of cervical cancer screening, understanding that failure to identify and address potential barriers could impact HPV-based screening program implementation.

Methodology or Methods

A nationally representative sample of screening-eligible Canadians (*n* = 3724) completed a web-based survey in summer 2022. Oversampling ensured that half of the sample were underscreened for cervical cancer (>3 years since previous screening or never screened). Participants completed validated scales of cervical cancer, HPV, and HPV test knowledge and HPV test and self-sampling attitudes and beliefs. Between-group differences (underscreened vs. adequately screened) were calculated for scales and items using independent sample t-tests or chi-square tests.

Impact on Practice or Results

Underscreened participants (*n* = 1871) demonstrated significantly lower knowledge scale scores and had lower awareness of the causal role of HPV in cervical cancer (81.6% vs. 74.7%). In the full sample, 75.9% did not know the difference between HPV+ and abnormal Pap test results. Adequately screened participants (*n* = 1853) exhibited higher scores for subscales of HPV test ‘Confidence’ and ‘Worries’, while underscreened participants (*n* = 1871) demonstrated greater scores on subscales related to ‘Personal barriers’ of HPV testing and the importance of ‘Social norms’. Underscreened participants had higher ‘Concerns’ but endorsed greater ‘Autonomy’ provided by self-sampling.

Discussion or Conclusions

For underscreened Canadians, messaging should emphasize factors that facilitate screening (e.g., convenience), explain the causal role of HPV in cervical cancer, and encourage the development of shared norms for cervical screening. Messaging for adequately screened women should provide reassurance about changes and emphasize the benefits of HPV primary screening.

130

### 13.5. Quitting Nicotine Use: A Qualitative Comparison of Cessation Experiences of Smokers, Vapers, and Dual Users

Ramona Sharma and Laura Struik

The University of British Columbia, Kelowna, Canada

Background/Rationale or Objectives/Purpose

Nicotine use is considered the most significant man-made cause of cancer that is preventable. However, there is limited qualitative research available on the unique nuances between different types of nicotine users, i.e., cigarette users (smokers), e-cigarette users (vapers), and users of both cigarettes and vapor products (dual users) with respect to cessation. This study took a comparative focus on motivations behind and experiences surrounding nicotine cessation among smokers, vapers, and dual users. Specific objectives included exploring and comparing: (a) motivations behind quitting, (b) barriers and facilitators to quitting, and (c) preferences for web-based cessation supports.

Methodology or Methods

Semi-structured qualitative interviews with British Columbia smokers, vapers, and dual users motivated to quit (*n* = 36) were audio-recorded, transcribed, and coded using classical content analysis and NVivo software to compile emergent experiential themes.

Impact on Practice or Results

All nicotine users cited desires to improve health as a primary motivation to quit, with vapers highlighting step-by-step guides on quitting as motivating. All discussed stress, boredom, and being around other users as significant barriers to quitting; however, vapers discussed the societal normalization of “vaping as less harmful” as a specific barrier. All equally discussed social supports as key in facilitating cessation and reduction in use, with dual users noting having distractions as key. Finally, most vapers discussed a need for instant support (e.g., live chat) as a crucial unmet need in existing cessation supports.

Discussion or Conclusions

Findings identify unique needs in terms of cessation journeys and allow for supports to better target a diverse array of nicotine user types.

## 14. Final Category: L. Innovation in Psychosocial Oncology Interventions

16

### 14.1. Effects of Oncology Camp on the Psychosocial Health of Childhood Cancer Patients

Sarah O’Connell ^1^, Nathan O’Keeffe ^1^, Leslie Kerr ^1,2^, Greg D. Wells ^3^ and Sarah L. West ^2,4^

1. Environmental and Life Sciences, Graduate Studies, Trent University, Peterborough, Canada
2. Department of Biology, Trent University, Peterborough, Canada
3. Translational Medicine, SickKids Research Institute, Toronto, Canada
4. Department of Kinesiology, Trent University, Peterborough, Canada

Background/Rationale or Objectives/Purpose

Objectives/purpose: Childhood cancer patients encounter significant adversity, therefore experiences promoting psychosocial health are necessary. This study determined the impact of oncology camp (OC) on the resilience, hope, social functioning, mental well-being, and stress of childhood cancer patients.

Methodology or Methods

Methods: Childhood cancer patients (6–18 years) enrolled in a 12-day session of OC at Campfire Circle (Muskoka, Ontario, Canada) were invited to participate. Participants completed a survey on the first (T1) and last day (T2) of camp, and 3 months post-camp (T3). This survey included the: Child and Youth Resilience Measure (CYRM-R), Children’s Hope Scale (CHS), Social Provisions Scale (SPS-5), and Short Warwick-Edinburgh Mental Wellbeing Scale (SWEMWBS). Afternoon saliva samples were collected at T1 and T2 to determine cortisol (ELISA). Repeated-measures ANOVAs evaluated differences in survey scores between all timepoints. A paired t-test evaluated differences in salivary cortisol (T1 vs. T2).

Impact on Practice or Results

Results: Ten participants (14.1 ± 2.5 years) were included in the analysis. CYRM-R, SPS-5, and SWEMWBS scores were high but did not differ between timepoints. CHS scores did not differ between T1 and T2; however, T3 (23.70 ± 7.364) was lower compared to T1 (28.30 ± 5.889; *p* = 0.004) and T2 (29.30 ± 6.717; *p* = 0.018). Salivary cortisol levels were within normal age-based ranges.

Discussion or Conclusions

Conclusion and clinical implications: While at OC, childhood cancer patients have high levels of resilience, hope, social support, and mental well-being, as well as normal stress levels. CHS scores decreased 3 months post-camp suggesting that continued psychosocial interventions may be necessary. Overall, the OC environment is associated with positive psychosocial health.

24

### 14.2. Examining Engagement in a Self-In-Relationship Observation Exercise by Couples Coping with Breast Cancer: A Qualitative Analysis of Text-Based Feedback

Sami Harb ^1^ and Karen Fergus ^1,2^

1. York University, Toronto, Canada
2. Sunnybrook Health Sciences Centre, Toronto, Canada

Background/Rationale or Objectives/Purpose

We examined how couples coping with breast cancer had engaged in a self-in-relationship observation exercise, and in what ways they may or may not have derived benefit from doing so.

Methodology or Methods

Participants were asked to: (1) observe their own ‘turning towards and away’ behaviours and their partner’s ‘turning towards’ behaviours (Gottman, 2011) deemed to contribute to relationship closeness or distance; and (2) independently, textually describe such interactions across ≥ one week. Upon exercise completion, partners provided feedback via a 5-point Likert scale and open-ended text boxes pertaining to perceptions of liking, benefitting, and learning from the exercise.

Impact on Practice or Results

A thematic analysis of qualitative feedback yielded four themes: “Receptivity to exercise”, “Shifting how I *attend* in relationship”, “Generated insight”, and “Shifting how I *engage* in relationship”. Partners practiced a type of in vivo awareness of relationship moments, reflected on relationship events and patterns, and/or adjusted how they act in relationship as a result of observing themselves.

Discussion or Conclusions

Our findings suggest that an online exercise promoting awareness of relationship interactions was feasible and acceptable for a majority of participants. Many participants reported positive exercise experiences or relationship enhancement, including slowing down of one’s processing of the relationship, reinforcing significance of seemingly small acts of care, and acting with the intention of achieving greater closeness with one’s partner. Drawbacks associated with the exercise primarily pertained to difficulty with dedicating time or effort to it, or with tracking interactions. Our results indicate factors which may facilitate or impede participants’ engagement with a couple-based interpersonal-awareness-exercise.

58

### 14.3. An Evidence-Based Framework for an Innovative Provincial Psychosocial Oncology Program in Nova Scotia

Katrin Julia Kaal ^1,2^, Joy Tarasuk ^3^, Marianne Arab ^3^, Janice Howes ^3^, Mark Embrett ^2^, Logan Lawrence ^2^, Tara Sampalli ^2^, Gail Tomblin Murphy ^2^, Helmut Hollenhorst ^3^, Margaret Ann Morrison ^3^ and Robin Urquhart ^1^

1. Dalhousie University, Halifax, Canada
2. Nova Scotia Health, Halifax, Canada
3. Cancer Care Program, Nova Scotia Health, Halifax, Canada

Background/Rationale or Objectives/Purpose

There is a documented need for an innovative approach to organize and deliver psychosocial oncology (PSO) services in Nova Scotia to address the multifaceted impact of cancer on patients and families. The aim of this project is to develop an evidence-based provincial PSO framework, that extends beyond traditional PSO providers.

Methodology or Methods

Prospective evidence gathering will be guided by the 3-I framework (interests, ideas, and institutions) and include (1) a rapid systematic review of the academic/grey literature; (2) a horizon scan of other Canadian provinces and comparable international health systems to identify critical factors associated with and the extent to which other jurisdictions have implemented PSO programs; (3) key informant interviews with a) relevant individuals in Canadian provinces and international jurisdictions and b) Nova Scotia-based stakeholders to understand the current context and identify key dimensions and structures to inform the design of a provincial PSO program framework that applies an extended, strength-based approach to PSO.

Impact on Practice or Results

Applying an extended approach to PSO care with an emphasis on strengthening ties to the community, will improve health outcomes, lead to better experiences for patients and PSO HCPs, reduce reliance on the healthcare system (i.e., reduce costs) and reduce inequities in access (i.e., achieve the quintuple aim).

Discussion or Conclusions

The deliverable is an overarching framework for a provincial PSO program, the top three options for operationalizing the framework, implications, implementation considerations of each option, and specific knowledge mobilization activities that will serve to socialize relevant stakeholders to its concepts and content.

72

### 14.4. Thoughts and Anticipations about Cancer: Psychometric Properties of a Questionnaire and Relationships with Psychological Symptoms

Adèle Jobin-Théberge ^1,2^, Claudia Mc Brearty ^1,2^, Guillaume Foldes-Busque ^1,3^, Anne-Josée Guimond ^1,2^, Lynn Gauthier ^2,4^, Véronique Massicotte ^1,2^ and Josée Savard ^1,2^

1. School of Psychology, Université Laval, Quebec, Canada
2. Université Laval Cancer Research Center, Quebec, Canada
3. Research Center of the Centre Intégré de Santé et de Services Sociaux de Chaudière-Appalaches, Quebec, Canada
4. Faculty of Medicine, Université Laval, Quebec, Canada

Background/Rationale or Objectives/Purpose

To assess the psychometric properties of the Thoughts and Anticipations about Cancer questionnaire (TAC) and to evaluate the cross-sectional and prospective associations between the different thinking orientations and psychological symptoms before and after cancer treatment.

Methodology or Methods

This study is a secondary analysis of data from three studies conducted at the same research center. All participants were females and treated for non-metastatic breast cancer. A total of 242 women with breast cancer were recruited prior to beginning their chemotherapy or radiotherapy. The TAC was completed prior to treatment (T0) and psychological symptoms, including depression, anxiety, fear of cancer recurrence (FCR), insomnia, fatigue and pain, were assessed at T0 and at post-treatment (T1).

Impact on Practice or Results

The factor analysis conducted on the 37-item TAC revealed a clear structure with four factors for the negative subscale and three factors for the positive subscale. Results also supported the internal consistency (negative subscale; α = 0.90, positive subscale; α = 0.93) of the scale, as well as its convergent validity with similar constructs (i.e., optimism and pessimism; r = 0.38 to 0.48) and divergent validity with opposed constructs (r = −0.20 to −0.46). In addition, repeated measures analyses of variance indicated that, in general, patients with a positive thought orientation and not future-oriented patients reported lower levels of psychological symptoms as compared to those with a negative or a realistic orientation.

Discussion or Conclusions

This study suggests that the French-Canadian version of the TAC is a reliable and valid instrument to evaluate pre-treatment thought orientations in patients with cancer and to investigate their relationship with patients’ psychological symptoms.

78

### 14.5. Lessons Learned from a Pilot Randomized Controlled Trial to Enhance Patients’ Adherence to Oral Anticancer Agents

Saima Ahmed ^1,2^ and Carmen G. Loiselle ^1,2^

1. McGill University, Montreal, Canada
2. Segal Cancer Centre, CIUSSS du Centre-Ouest-de l’Île-de Montréal, Montreal, Canada

Background/Rationale or Objectives/Purpose

A two-arm, pilot randomized controlled trial was conducted to assess the feasibility, acceptability, and preliminary effects of a comprehensive supportive oral anti-cancer agent (OAA) intervention promoting medication adherence. Herein, we report on lessons learned regarding study implementation.

Methodology or Methods

A total of 52 participants (26 per arm) were recruited from a large university-affiliated cancer centre in Montreal. Inclusion criteria were ≥18 years old, diagnosis of cancer, any stage, in their first cycle (systemic, targeted, or hormonal as active treatment), with internet access. Exclusion criteria were having received IV chemotherapy, immunotherapy and/or maintenance hormonal therapy, significant physical or cognitive limitations, imminent “end-of-life”, or participating in an ongoing clinical trial.

Participants completed baseline e-questionnaires and were randomized to the experimental or usual care group. Study intervention: (1) OAA-related handouts, videos, and supportive resources, (2) nurse-led phone follow-ups (if required), and (3) e-reminders to take OAAs. Follow-up questionnaires were completed every 1–2 weeks for 5 months, or until treatment was completed, followed by an exit questionnaire. A subset (8–12 per arm) participated in in-depth semi-structured interviews.

Impact on Practice or Results

To date, 47 participants have been recruited, 28 completed exit questionnaires and 17 participated in interviews. Recruitment challenges included pandemic-related restricted in-person access, intense clinician workloads, and breaks in patient contact. Furthermore, identification of an optimal adherence measure was difficult. To address these, targeted OAAs were included, online recruitment, and exchanges with pharmacists, nurses, and oncologists helped.

Discussion or Conclusions

Conducting RCTs is challenging under the best conditions. Given the pandemic and OAA adherence being outside routine clinical care, creative solutions optimized study implementation and outcomes.

102

### 14.6. Feasibility Study of a Psychotherapeutic Intervention Adapted for Family Caregivers of Children Newly Diagnosed with Acute Leukemia: Emotion and Symptom-Focused Engagement (EASE)

Ally Yu ^1,2^, Stephanie Nanos ^1,3^, Carmine Malfitano ^1,3^, Sarah Alexander ^2,3^, Camilla Zimmermann ^1,2^, Lindsay Jibb ^2,3^ and Gary Rodin ^1,2^

1. Princess Margaret Cancer Centre, Toronto, Canada
2. University of Toronto, Toronto, Canada
3. The Hospital for Sick Children (SickKids), Toronto, Canada

Background/Rationale or Objectives/Purpose

Acute leukemia (AL) is the most common childhood cancer, often requiring urgent hospitalization, intensive treatment, and causing significant traumatic stress (TS) for family caregivers (FCs). We developed the Emotion- and Symptom-focused Engagement (EASE) intervention to prevent or reduce TS in adult cancer patients or FCs. The EASE intervention integrates problem-solving, relational support, affect regulation, and cognitive behavioural techniques. Using a mixed-methods single-arm pilot trial, we aim to assess the feasibility and acceptability of EASE for FCs of children with AL.

Methodology or Methods

We are recruiting 40 FCs of children newly diagnosed with AL at the Hospital for Sick Children in Toronto, Canada. FCs receive ≤12 EASE sessions over 3 months, based on perceived need and feasibility. Measures assessing TS, depression, caregiver burden, and satisfaction with care are administered at baseline, 1, 3, 6, 9, and 12 months. Using a priori established criteria, trial feasibility is evaluated as study accrual, attrition, intervention adherence, and outcome measure completion. Semi-structured interviews are conducted with ~20 FCs to assess acceptability. Interviews are analyzed using qualitative descriptive design and thematic analysis.

Impact on Practice or Results

Based on evidence of EASE feasibility and acceptability, we are developing a subsequent randomized controlled trial to assess its effectiveness in reducing TS among FCs of children with cancer.

Discussion or Conclusions

Findings from this study will enhance our understanding of the potential effectiveness of a proactive psychotherapeutic intervention in preventing or reducing TS among FCs. This research has the potential to expand the evidence base for the value of proactive psychosocial interventions for FCs and other high-risk populations.

103

### 14.7. Creating Safe Online Space for Cancer Support: How Moderation Practices for the Canadian Cancer Society’s CancerConnection.ca Online Community Contribute to High Quality Outcomes

Danielle VandeZande ^1^, Lacey Horta ^2^, Lianne Wilson ^3^, Holly Bradley ^1^ and Laura Burnett ^2^

1. Canadian Cancer Society, Toronto, Canada
2. Canadian Cancer Society, Hamilton, Canada
3. Canadian Cancer Society, Vancouver, Canada

Background/Rationale or Objectives/Purpose

The Canadian Cancer Society’s CancerConnection.ca program is an online community that supports more than 280,000 visitors and 9000 active members annually. In this peer support community, people with cancer and their caregivers find meaningful connection, emotional support and share information from the lived experience of cancer.

Online communities have potential for substantial reach. They are also complex support systems that are guided by a growing knowledge base of industry best practices and research about user behaviour and supportive care outcomes. It is important that leading-edge knowledge inputs be applied to operational practices and that user experience outcomes are measured. Especially in the context of cancer support where online communities include sensitive personal information and potential distress, a safe environment with responsive moderation are keys to success.

Methodology or Methods

The Canadian Cancer Society’s online peer support community CancerConnection.ca will be demonstrated, and quantitative and qualitative analyses from the community will be shared to illustrate the impact of CCS’s moderation practices on user safety and cancer support outcomes.

Impact on Practice or Results

Moderation strategies are core community management techniques that require careful consideration, resources, training, and community engagement. They are also important to ensuring that the integrity of the peer support model is maintained and that users are directed to other healthcare services that extend beyond its boundaries.

Discussion or Conclusions

CCS’s moderation strategy exceeds industry best practices. It will serve as an excellent example of an evidence-informed approach resulting in a safe peer-support environment and high-quality cancer support outcomes.

127

### 14.8. Improving the Experience and Adherence to Adjuvant Endocrine Therapy after Breast Cancer: A Mixed-Method Pilot Randomized Controlled Trial

Sophie Lauzier ^1,2^, Victoria Memoli ^3^, Odilon Assan ^4^, Véronique Turcotte ^2^, Laurence Guillaumie ^2,5^, Julie Lemieux ^6^, Carolyn Gotay ^7^ and Marijn deBruin ^8^

1. Faculty of pharmacy, Université Laval, Québec, Canada
2. CHU de Québec Research Center, Québec, Canada
3. Aix Marseille Univ, INSERM, IRD, SESSTIM, ISSPAM, Cancer Biomedicine and Society Group, Equipe Labellisée Ligue 2019, Marseilles, France
4. VITAM Centre de recherche en santé durable, Québec, Canada
5. Faculty of nursing, Université Laval, Québec, Canada
6. Centre des maladies du sein, CHU de Québec, Québec, Canada
7. School of Population and Public Health, University of British Columbia, Vancouver, Canada
8. Radboud Institute for Health Sciences, Nijmegen, Netherlands

Background/Rationale or Objectives/Purpose

Five to ten years of adjuvant endocrine therapy (AET) reduces breast cancer recurrence. However, 31–47% of women discontinue AET prematurely. We designed the SOIE program, including an educational group session, nurse navigator consultations and online chat sessions to improve AET experience and adherence. We assessed its acceptability, feasibility and impact on psychosocial factors, precursors of AET adherence.

Methodology or Methods

A single-center 12-month mixed-method pilot RCT was conducted among women initiating AET. Psychosocial factors based on the Theory of Planned Behavior (TPB) (intention to persist with AET, attitude, behavioural control) and additional constructs (knowledge, support, coping planning) and adherence were measured at baseline, 3 and 12 months. Group patterns were compared using generalized estimating equations. In the intervention group, 20 women participated in a semi-structured interview.

Impact on Practice or Results

A total of 106 women were randomized (SOIE: 52; control: 54). *Quantitative*: Both groups reported high scores on TPB constructs (mean ≥ 6.0/7.0), and patterns over time were not statistically different. The intervention group reported greater increases in AET knowledge and coping planning (group*time *p*-value ≤ 0.002) and better daily AET intake (*p* = 0.013). More than 90% of participants reported positive impacts and were satisfied. *Qualitative*: SOIE was described as meeting needs (being informed, supported, reassured, and able to cope with side effects) and helping to develop a more positive attitude towards AET, contributing to the intention to continue AET.

Discussion or Conclusions

SOIE is feasible and highly valued. In the first year of AET, it reinforced feelings of being supported and prepared to face AET difficulties, which can help to prevent a decline in long-term persistence.

## 15. Final Category: M. Health Care Provider Wellness

23

### 15.1. Team Resilience at Work in Oncology during the Pandemic: Do We Have Valid Measurement Tools?

Dominique Tremblay ^1,2^, Djamal Berbiche ^2^ and Sylvie Lessard ^2^

1. Université de Sherbrooke-Campus Longueuil, Longueuil, Canada
2. Centre de recherche Charles-Le Moyne, Longueuil, Canada

Background/Rationale or Objectives/Purpose

Chronic exposure to the suffering of cancer patients, exacerbated by the pandemic, has been associated with a greater burden of stress on healthcare professionals (HCPs) in oncology. Team resilience at work (Team R@W) can strengthen team members’ capacity to cope with stressors and help protect their health and well-being. In a study of coping capacities in oncology teams, we employ McEwen’s original Team R@W instrument, and assess its validity and feasibility in this context.

Methodology or Methods

The original Team R@W French language instrument was adapted to the healthcare system and culture in Québec, and pilot-tested within a larger cross-sectional study (2021–2022). Participants (*n* = 209) from four oncology settings completed an online questionnaire: R@W Team—42 items, 7 dimensions on a seven-point scale and sociodemographic characteristics. Descriptive statistics (mean, standard deviation, median) serve to examine distribution. Internal consistency (Cronbach’s Alpha) and correlation analysis were performed.

Impact on Practice or Results

The response rate (26%) was deemed acceptable considering that lower rates are expected in specialized healthcare sectors. Internal consistency is excellent and similar to that achieved with the original instrument: Cronbach’s Alpha for the full scale was 0.95, ranging from 0.92 to 0.83 for the dimensions. All dimensions had high impact. The lowest score was for Self-care (mean = 4.32; SD = 1.41) and the highest was for Perseverance (mean = 5.28; SD = 1.12).

Discussion or Conclusions

Results support the validity of the measurement instrument for oncology team members. Lower Self-care scores indicate an opportunity to develop interventions in oncology teams. Future research should focus on the relationship between Team R@W and organizational and oncology team characteristics.

81

### 15.2. Innovations in Mentorship: Implementation of a Comprehensive Mentorship Program for Faculty in a Psychiatry Department: Implications for Psycho-Oncology

Mary Jane Esplen ^1^, Shaheen Darani ^1^, John Teshima ^1^, Certina Ho ^1^, Krista Lanctot ^1^, Jiahui Wong ^2^, Nicole Kozloff ^1^ and Danica Kwong ^3^

1. University of Toronto, Toronto, Canada
2. de Souza Institute, Toronto, Canada
3. University of Toronto, Toronto, Canada

Background/Rationale or Objectives/Purpose

Mentorship supports academic development and wellness. At a large Department of Psychiatry, a recent survey showed 60% faculty were without mentors and would like to have one; 75% of mentors received no training.

A comprehensive mentorship program was implemented department-wide to facilitate sharing of expertise, self-reflection, career development and wellness.

Methodology or Methods

Program design was informed by a literature review and a mentorship working group. A central component is a primary mentor–mentee relationship, further supported by mentorship groups focused on academic roles or social identity. Equity, Inclusion and Diversity (EDI) is considered throughout the design, implementation and evaluation. An online matching tool, based on faculty academic interests, roles and social identity preferences supports mentee/mentor pairing. A logic model informs a three-year evaluation plan and explores perception and concepts, such as intersectionality, wellness, and EDI.

Impact on Practice or Results

The program was launched with virtual workshops offering best practices and reflection on challenges encountered during mentorship. Fifty-seven faculty mentors and eighty-eight faculty mentees attended. Training was provided in EDI and mentorship, best practices, and content supporting academic roles. Feedback was positive; 93% participants indicated the workshops met learning objectives; 80% rated the workshops as excellent. Eighty-seven percent of mentors reported increased awareness of best practices. Mentorship groups focusing on social identity have been well-received and include communities of practice for: women, LGBTQ2S+, racialized women and international medical graduates.

Discussion or Conclusions

A comprehensive program with the goal to address EDI and career support demonstrates benefit. Preliminary findings will be presented with implications for Psycho-Oncology.

## 16. Final Category: N. Cancer Treatment-Related Symptom and Toxicity Management

25

### 16.1. The Impact of Genetic Predispositions to Depression on Quality of Life and Survival in Patients with Head and Neck Cancer Immediately Post-Treatment: A Longitudinal Study

Melissa Henry ^1^, Lawrence Chen ^1^, Michael Meaney ^1^, Zeev Rosberger ^1^, Michael Hier ^2^, Anthony Zeitouni ^1^, Nader Sadeghi ^1^, Khalil Sultanem ^1^, Fabio Cury ^1^ and Kieran O’Donnell ^3^

1. McGill University, Montreal, Canada
2. McGill University, Montreal, Canada
3. Yale University, Connecticut, USA

Background/Rationale or Objectives/Purpose

The primary purpose of this study was to investigate the contribution of genetic predisposition to depression, as measured through polygenic risk scores (PRS), on levels of quality of life in patients with head and neck cancer (HNC) in the immediate post-treatment period (i.e., at three months post-diagnosis) and on 3-year survival.

Methodology or Methods

Prospective longitudinal study of 223 consecutive adults (72% participation) newly diagnosed with a first occurrence of primary HNC, including genetic data to construct PRS for depression, validated psychometric measures, Structured Clinical Interviews for DSM Disorders, and medical chart reviews.

Impact on Practice or Results

Level of quality of life at 3 months was predicted by (*R*^2^ = 0.51, *R*^2^ adj. = 0.33, *p* = 0.001) the polygenic risk score (PRS) for depression (standardized b = −0.28, *p* = 0.01) and a previous history of suicidal ideation (standardized b = −0.25, *p* = 0.04). PRS for depression predicted 3-year survival (b = 1.75, Exp(B) = 5.75, 95%CI = 1.55–21.27, *p* = 0.009). Other variables were non-significant in the analyses: sociodemographic (i.e., age, sex, education, living alone), psychosocial (i.e., SCID current and past diagnoses (trend), past history of abuse), and medical variables (i.e., cancer stage and site, HPV status, functional status/ECOG, treatment).

Discussion or Conclusions

Our results outline the importance of attending to genetic predisposition and past history of suicidal ideation as markers for quality of life compromise immediately post-treatment in patients with head and neck cancers, as well as of considering targeting genetic predisposition towards depression implicated in survival. Strategies are needed to address psychosocial vulnerability early on as part of pre-habilitation in the treatment of patients with head and neck cancer.

38

### 16.2. Evolution of Anxiety in Breast Cancer Patients: A Prospective Analysis Using Clinical, Biological, and Genetic Factors

Roula Hachem ^1,2^, Michelle Bedran ^1^, Rita Khoury ^1,2^, Pascale Salameh ^3,4^, Hala Sacre ^5^, Georges Chahine ^6^, Joseph Kattan ^6^, Lydia Rabbaa Khabbaz ^1,2^ and Aline Hajj ^1,7^

1. Faculty of Pharmacy, Saint Joseph University of Beirut, Beirut, Lebanon
2. Laboratoire de Pharmacologie, Pharmacie Clinique et Contrôle de Qualité des Médicaments, Saint Joseph University of Beirut, Beirut, Lebanon
3. School of Medicine, Lebanese American University, Byblos, Lebanon
4. Department of Primary Care and Population Health, University of Nicosia Medical School, Nicosia, Cyprus
5. INSPECT-LB (Institut National de Santé Publique, d’Épidémiologie Clinique et de Toxicologie-Liban), Beirut, Lebanon
6. Department of Hemato-Oncology, Hôtel-Dieu de France Hospital, Faculty of Medicine, Saint Joseph University of Beirut, Beirut, Lebanon
7. Faculté de Pharmacie, Université Laval, Québec City, Canada

Background/Rationale or Objectives/Purpose

Assess the prospective evolution of anxiety among breast cancer patients over eight consecutive chemotherapy cycles, taking into account sociodemographic, clinical, biological, and genetic factors.

Methodology or Methods

A prospective longitudinal study was conducted among 69 breast cancer patients treated by intravenous chemotherapy at the oncology outpatient unit of Hôtel-Dieu de France hospital (2017–2019; Ethics: CEHDF1016). The Hospital Anxiety and Depression Scale (HADS) was applied to evaluate anxiety and depression. Other validated scales were used to assess cognitive function, sleep disorders, fatigue, and pain. Genotyping was performed for several genes (*ABCB1*, *COMT*, *DRD2*, *OPRM1*, *CLOCK*, *CRY2*, *PER2*) using the Lightcycler^®^ 2.0 (Roche).

Impact on Practice or Results

Univariate repeated measures analysis shows a decrease in anxiety scores between cycles 1 and 6 of chemotherapy, followed by an increase starting cycle 6 (a polynomial trend for contrasts) (*p*-value_cycle 6 versus 1_ = 0.038; *p*-value_cycle 4 versus 1_ = 0.067). Multivariable analysis showed that higher anxiety and depression scores at baseline and lower alcohol consumption were associated with higher anxiety scores over time. Moreover, patients carrying at least one G variant allele for the *OPRM1* polymorphism and one A variant allele for the *PER2* polymorphism were associated with higher anxiety scores over time, whereas patients with at least one variant T allele for *ABCB1* exhibited lower HADS-Anxiety scores over time.

Discussion or Conclusions

Our findings showed a pattern of anxiety’s evolution through chemotherapy cycles in breast cancer patients and highlighted the importance of identifying the triggering factors and implementing an individualized management plan, thus improving patients’ quality of life.

39

### 16.3. Evolution of Depression among Breast Cancer Patients: A Prospective Analysis Using Clinical, Biological and Genetic Factors

Roula Hachem ^1,2^, Rita Khoury ^1,2^, Pascale Salameh ^3,4^, Hala Sacre ^5^, Georges Chahine ^6^, Fady Nasr ^6^, Lydia Rabbaa Khabbaz ^1,2^ and Aline Hajj ^1,7^

1. Faculty of Pharmacy, Saint Joseph University, Beirut, Lebanon
2. Laboratoire de Pharmacologie, Pharmacie Clinique et Contrôle de Qualité des Médicaments, Saint Joseph University, Beirut, Lebanon
3. School of Medicine, Lebanese American University, Byblos, Lebanon
4. Department of Primary Care and Population Health, University of Nicosia Medical School, Nicosia, Cyprus
5. INSPECT-LB (Institut National de Santé Publique, d’Épidémiologie Clinique et de Toxicologie-Liban), Beirut, Lebanon
6. Department of Hemato-Oncology, Hôtel-Dieu de France Hospital, Faculty of Medicine, Saint Joseph University of Beirut, Beirut, Lebanon
7. Faculté de Pharmacie, Université Laval, Québec City, Canada

Background/Rationale or Objectives/Purpose

Assess the prospective evolution of depression among breast cancer patients over eight consecutive chemotherapy cycles, taking into account sociodemographic, clinical, biological, and genetic factors.

Methodology or Methods

A prospective longitudinal study was conducted among 69 breast cancer patients treated by intravenous chemotherapy at the oncology outpatient unit of Hôtel-Dieu de France hospital (2017–2019; Ethics: CEHDF1016). The Hospital Anxiety and Depression Scale (HADS) was applied to evaluate anxiety and depression among patients. Other validated scales were used to assess cognitive function, sleep disorders, fatigue, and pain. Genotyping was performed for several genes (*ABCB1*, *COMT*, *DRD2*, *OPRM1*, *CLOCK*, *CRY2*, *PER2*) using the Lightcycler^®^ 2.0 (Roche Diagnostics GmbH, Mannheim, Germany).

Impact on Practice or Results

Univariate repeated measures analysis showed a polynomial linear contrast for HADS-D scores from cycle 1 to cycle 8, with a significant increase in depression noted at cycles 7 and 8 compared to cycle 1 (*p*-value_cycle7_ = 0.004 and *p*-value_cycle8_ = 0.009). Multivariable analysis showed that lower hemoglobin levels, higher depression, anxiety, and fatigue scores at baseline were associated with higher depression scores over time. Moreover, carrying at least one Met variant allele for the *COMT* rs4680 polymorphism, and carrying at least one G variant allele for the *PER2* polymorphism were associated with lower depression scores over time.

Discussion or Conclusions

Our findings highlight the importance of identifying markers to improve the understanding of depression trajectories in women with breast cancer and implement personalized medicine approaches to mitigate detrimental health outcomes at specific turning points.

43

### 16.4. Evolution of Cognitive Disorders among Breast Cancer Patients: A Prospective Analysis Using Clinical, Biological, and Genetic Factors

Rita Khoury ^1,2^, Roula Hachem ^1,2^, Pascale Salameh ^3,4^, Elyssa Fares ^1^, Hala Sacre ^5^, Georges Chahine ^6^, Joseph Kattan ^6^, Lydia Rabbaa Khabbaz ^1,2^ and Aline Hajj ^1,7^

1. Faculty of Pharmacy, Saint Joseph University of Beirut, Beirut, Lebanon
2. Laboratoire de Pharmacologie, Pharmacie Clinique et Contrôle de Qualité des Médicaments, Saint Joseph University of Beirut, Beirut, Lebanon
3. School of Medicine, Lebanese American University, Byblos, Lebanon
4. Department of Primary Care and Population Health, University of Nicosia Medical School, Nicosia, Cyprus
5. INSPECT-LB (Institut National de Santé Publique, d’Épidémiologie Clinique et de Toxicologie-Liban), Beirut, Lebanon
6. Department of Hemato-Oncology, Hôtel-Dieu de France Hospital, Faculty of Medicine, Saint Joseph University of Beirut, Beirut, Lebanon
7. Faculté de Pharmacie, Université Laval, Québec City, Canada

Background/Rationale or Objectives/Purpose

Assess the prospective evolution of cognitive function among breast cancer patients over eight consecutive chemotherapy cycles, taking into account sociodemographic, clinical, biological, and genetic factors.

Methodology or Methods

A prospective longitudinal study was conducted among 69 breast cancer patients treated by intravenous chemotherapy at the oncology outpatient unit of Hôtel-Dieu de France hospital (2017–2019; Ethics: CEHDF1016). The 37-item Functional Assessment of Cancer Therapy-Cognitive Function (FACT-Cog) was applied to evaluate cognitive function. Other validated scales were used to assess depression, anxiety, sleep disorders, fatigue, and pain. Genotyping was performed for several genes (*ABCB1*, *COMT*, *DRD2*, *OPRM1*, *CLOCK*, *CRY2*, *PER2*) using the Lightcycler^®^ 2.0 (Roche Diagnostics GmbH, Mannheim, Germany).

Impact on Practice or Results

Univariate repeated measures analysis showed a decrease in the FACT-Cog total scale and subscales from cycle 1 to cycle 8; overall cognition was significantly lower starting cycle 4. Multivariable analysis showed that cognition decreased in patients with higher depression, anxiety, and fatigue scores at baseline and those with at least one Val allele for the *COMT* rs4680 polymorphism. However, higher cognitive functioning was noted among married working women and those with higher socioeconomic status, higher levels of pain and insomnia, and those carrying at least one A allele for the *PER2* rs934945 polymorphism, taking into account baseline cognition.

Discussion or Conclusions

Our findings highlight the importance of assessing cognitive decline trajectories in women with breast cancer and identifying markers to improve the implementation of personalized interventions, thus improving their functionality and productivity.

44

### 16.5. Cluster Symptoms in Breast Cancer Patients: An Analysis Using Clinical, Biological, and Genetic Factors

Rita Khoury ^1,2^, Nathalie Saab ^1^, Pascale Salameh ^3,4^, Roula Hachem ^1,2^, Emile Abou Chaar ^1,5^, Hala Sacre ^6^, Georges Chahine ^7^, Fady Nasr ^7^, Lydia Rabbaa Khabbaz ^1,2^ and Aline Hajj ^1,8^

1. Faculty of Pharmacy, Saint Joseph University of Beirut, Beirut, Lebanon
2. Laboratoire de Pharmacologie, Pharmacie Clinique et Contrôle de Qualité des Médicaments, Saint Joseph University of Beirut, Beirut, Lebanon
3. School of Medicine, Lebanese American University, Byblos, Lebanon
4. Department of Primary Care and Population Health, University of Nicosia Medical School, Nicosia, Cyprus
5. Institut supérieur de sciences religieuses, Saint Joseph University, Beirut, Lebanon
6. INSPECT-LB (Institut National de Santé Publique, d’Épidémiologie Clinique et de Toxicologie-Liban), Beirut, Lebanon
7. Department of Hemato-Oncology, Hôtel-Dieu de France Hospital, Faculty of Medicine, Saint Joseph University of Beirut, Beirut, Lebanon
8. Faculté de Pharmacie, Université Laval, Québec City, Canada

Background/Rationale or Objectives/Purpose

Assess symptom clusters in breast cancer patients and explore the correlates with several sociodemographic, clinical, biological, and genetic factors.

Methodology or Methods

A prospective longitudinal study was conducted among 66 breast cancer patients treated by intravenous chemotherapy at the oncology outpatient unit of Hôtel-Dieu de France hospital (2017–2019; Ethics: CEHDF1016). Symptoms (cognitive function, sleep disorders, anxiety, depression, fatigue, and pain) were assessed using validated scales. Genotyping was performed for several genes (*ABCB1*, *COMT*, *DRD2*, *OPRM1*, *CLOCK*, *CRY2*, *PER2*) using the Lightcycler^®^ 2.0 (Roche Diagnostics GmbH, Mannheim, Germany).

Impact on Practice or Results

The cluster analyses allowed the identification of two distinct clusters: Cluster 1 (*n* = 26) was characterized by more severe psycho-neurological symptoms compared to Cluster 2 (*n* = 40). In particular, fatigue was about four times more severe for patients of Cluster 1, with a mean score of 75.64 versus 19.72 for Cluster 2. Multivariable analyses taking the distribution of patients in Cluster 1 vs. Cluster 2 as the dependent variable showed that patients exhibiting thrombocytopenia (Beta = 0.017), with a higher number of chemotherapy cycles (Beta = −0.431), or those carrying at least one variant T allele of the *DRD2* rs6277 polymorphism (Beta = 1.290) were significantly more prone to belong to Cluster 1 with more pronounced symptoms.

Discussion or Conclusions

Our findings highlight the importance of an adequate assessment of all factors affecting the clustering of symptoms in cancer patients so that an optimal patient outcome can be achieved by improving their quality of life and functional status.

49

### 16.6. Evolution of Sleep Disorders among Breast Cancer Patients: A Prospective Analysis Using Clinical, Biological, and Genetic Factors

Rita Khoury ^1,2^, Roula Hachem ^1,2^, Pascale Salameh ^3,4^, Sarah Harb ^1^, Hala Sacre ^5^, Emile Abou Chaar ^1,6^, Georges Chahine ^7^, Joseph Kattan ^7^, Lydia Rabbaa Khabbaz ^1,2^ and Aline Hajj ^1,8^

1. Faculty of Pharmacy, Saint Joseph University of Beirut, Beirut, Lebanon
2. Laboratoire de Pharmacologie, Pharmacie Clinique et Contrôle de Qualité des Médicaments, Saint Joseph University of Beirut, Beirut, Lebanon
3. School of Medicine, Lebanese American University, Byblos, Lebanon
4. Department of Primary Care and Population Health, University of Nicosia Medical School, Nicosia, Cyprus
5. INSPECT-LB (Institut National de Santé Publique, d’Épidémiologie Clinique et de Toxicologie-Liban), Beirut, Lebanon
6. Institut supérieur de sciences religieuses, Saint Joseph University, Beirut, Lebanon
7. Department of Hemato-Oncology, Hôtel-Dieu de France Hospital, Faculty of Medicine, Saint Joseph University of Beirut, Beirut, Lebanon
8. Faculté de Pharmacie, Université Laval, Québec City, Canada

Background/Rationale or Objectives/Purpose

Assess the prospective evolution of sleep disorders among breast cancer patients over eight consecutive chemotherapy cycles, taking into account sociodemographic, clinical, biological, and genetic factors.

Methodology or Methods

A prospective longitudinal study was conducted among 69 breast cancer patients treated by intravenous chemotherapy at the oncology outpatient unit of Hôtel-Dieu de France hospital (2017–2019; Ethics: CEHDF1016). Sleep disorders were evaluated using two screening tools, the Pittsburgh Sleep Quality Index (PSQI) and the insomnia severity index (ISI). Other validated scales were used to assess depression, anxiety, cognitive function, fatigue, and pain. Genotyping was performed for several genes (*ABCB1*, *COMT*, *DRD2*, *OPRM1*, *CLOCK*, *CRY2*, *PER2*) using the Lightcycler^®^ 2.0 (Roche).

Impact on Practice or Results

Univariate repeated measures analysis showed a slight decrease in PSQI and ISI scores between cycles 1 and 6 of chemotherapy, followed by an increase starting cycle 6 with a significant increase in PSQI score noted at cycle 8 compared to cycle 1 (*p*-value = 0.043). Multivariable analysis taking PSQI scores as the dependent variable showed that patients with lower alcohol consumption, higher BMI, higher anxiety and PSQI scores at baseline were associated with higher PSQI scores over time (worse sleep quality). Patients treated with neuropathic pain medication exhibited lower PSQI scores over time (better sleep quality). Polymorphisms in *COMT* (rs4680), *CLOCK* (rs1801260), *CRY2* (rs10838524) and *DRD2* (rs6277) were also significantly associated with PSQI scores over time.

Discussion or Conclusions

Our findings highlight the importance of assessing sleep disorders’ trajectories in women with breast cancer and identifying markers to improve personalized interventions and overall quality of life.

88

### 16.7. The Quality and Usefulness of Interactive Mobile Health Apps for Cancer Patients: Recommendations for Clinicians, Patients, and Families

Sydney Wasserman ^1^, Lydia Ould Brahim ^1^, Ameer Attiya ^1^, Eric Belzile ^2^ and Sylvie Lambert ^1,2^

1. McGill University, Montreal, Canada
2. St. Mary’s Research Centre, Montreal, Canada

Background/Rationale or Objectives/Purpose

To evaluate the quality and usefulness of publicly accessible interactive mobile health (mhealth) applications (apps) for adults with cancer to support them in managing their illness.

Methodology or Methods

Apps with an interactive tailored feature to help patients cope with cancer challenges were identified in 2020 and 2022 through searches of the Google Play store and a Google web search. Where applicable, the PRISMA guidelines were followed. Apps were evaluated for quality and usefulness using the Mobile App Rating Scale (MARS) and an ‘unmet needs’ checklist derived from the literature, respectively. Inter-rater reliability was established, and descriptive analyses were conducted to compare apps.

Impact on Practice or Results

A total of 1090 apps were identified, of which 19 were retained. The mean app quality score across the sample was 3.6/5 (SD 0.28, range: 3.1–4.1), with the highest scoring app being Outcomes4me. On average, apps addressed 46% (SD 2.73, range: 25–71%) of the unmet needs, with Cancer.net scoring the highest. “Emotional health” was the lowest scoring section of the unmet needs’ checklist whereas the subsection of the MARS scoring the lowest was “information” and the highest was “functionality”. Highest scoring apps were all developed by healthcare institutions, kept updated monthly, and shared features including self-monitoring of symptoms and medications.

Discussion or Conclusions

Interactive cancer-related apps were found to be of acceptable quality with less than 50% of unmet needs addressed. No single app scored highly on both measures. Addressing patients’ unmet needs, specifically emotional health, is an area for development in future apps.

## 17. Final Category: O. Pandemics and Cancer Care Issues

5

### 17.1. Cancer Care Disruptions during the COVID-19 Pandemic and Fear of Cancer Recurrence in Women with Breast Cancer

Claudia Mc Brearty ^1,2^, Laurie Bisaillon ^1,2^, Sophie Lauzier ^2,3^ and Josée Savard ^1,2^

1. School of Psychology, Université Laval, Quebec, Canada
2. Université Laval Cancer Research Center, Quebec, Canada
3. Faculty of Pharmacy, Université Laval, Quebec, Canada

Background/Rationale or Objectives/Purpose

Cancer patients have been particularly affected by the COVID-19 pandemic. In addition to being considered at a higher risk for complications from COVID-19, many cancer patients experienced delays in tests and treatments due to sanitary measures. Available data suggest that disruptions in cancer care during the pandemic negatively impacted patients’ mental health. However, there is a lack of evidence on the influence of disruptions in cancer care during the pandemic on fear of recurrence (FCR). FCR is defined as the fear, worry, or concern regarding the possibility that cancer will come back or progress. This study investigated the relationship between disruptions in cancer tests/treatments and FCR in women diagnosed with breast cancer in the past five years.

Methodology or Methods

A total of 245 women completed online questionnaires between November 2020 and March 2021.

Impact on Practice or Results

The results revealed that the proportion of patients showing a clinical level of FCR was significantly higher among women who had experienced postponement or cancellations of evolution tests (e.g., blood tests, X-rays or magnetic resonance imaging; 91.1% vs. 71.7%; *p* = 0.0001). No significant difference was observed for postponement or cancellation of other tests and treatments.

Discussion or Conclusions

These results highlight the importance of keeping on time diagnostic and evolution tests to prevent an increase in FCR in women treated for breast cancer.

9

### 17.2. People with Cancer Experience Worse Psychosocial and Financial Consequences of COVID-19 Compared to Healthy Individuals and Other Chronic Disease Populations: Findings from the International COVID-19 Awareness and Response Evaluation Study

Mohamad Baydoun ^1^, Andrew McLennan ^1^ and Linda Carlson ^2^

1. University of Regina, Regina, Canada
2. University of Calgary, Calgary, Canada

Background/Rationale or Objectives/Purpose

We comparatively investigated the psychosocial impacts of COVID-19 (C19) on healthy adults, adults living with chronic illness, and adults living with cancer, around the world.

Methodology or Methods

The study population included participants enrolled in the International C19 Awareness and Response Evaluation (iCARE) Study. In this secondary analysis, respondents were divided into three groups based on self-reported health status: (1) healthy; (2) active/current cancer (with or without other chronic conditions); and (3) other chronic illness, but not cancer. Linear regressions were conducted to evaluate the associations between health status and psychosocial outcomes.

Impact on Practice or Results

A total of 41,212 iCARE study respondents (X age = 47.3 years) from 175 countries worldwide, were included in the analysis, of whom 23,058 (56%) identified as healthy; 677 (1.6%) identified as having active/current cancer; and 17,477 (42.4%) identified as having other chronic illness. Relative to healthy individuals, significant associations were found between having cancer and higher C19-related feelings of sadness (β = 0.204, *p* = 0.012) and anxiety (β = 0.305, *p* < 0.001), as well as worsened overall quality of life (β = −0.201, *p* = 0.006). Cancer patients were also more likely than the remaining two groups to report financial hardships, such as inability to pay rent/mortgage (β = 0.264 (relative to healthy) and β = 0.249 (relative to other chronic illness), *p*s < 0.001).

Discussion or Conclusions

Worldwide, cancer patients tended to have worse psychosocial and financial challenges as a result of C19, compared with healthy individuals and other chronic disease populations. Clinicians need to be aware of the importance of attending to the specific mental health needs of cancer patients during ongoing C19-related restrictions.

54

### 17.3. Exploring Cancer Care Team Functioning during COVID-19

Samar Attieh ^1^ and Carmen G. Loiselle ^2,3^

1. Department of Experimental Medicine, Faculty of Medicine and Health Sciences, McGill University, Montreal, Canada
2. Department of Oncology and Ingram School of Nursing, Faculty of Medicine and Health Sciences, McGill University, Montreal, Canada
3. Segal Cancer Center, Jewish General Hospital, Montreal, Canada

Background/Rationale or Objectives/Purpose

During COVID-19, most cancer care teams faced significant challenges including acute work-related disruptions, rapid shifts in recommended health practices, and burnout. Within this context, it is crucial to better understand how serious public health crises affect cancer team functioning—through key indicators of team effectiveness (TE) and relational coordination (RC). This mixed-method study sought to bring together cancer care team members and individuals affected by cancer to explore their perceptions of TE and RC during COVID-19 (2021–2022) and as they transition out of the pandemic (2023).

Methodology or Methods

Sixty-six participants (thirteen healthcare professionals, forty patients, six caregivers, and seven trained volunteers) were recruited from gynecologic and breast cancer clinics at a large university-affiliated cancer center, in Montréal, Qc. Participants completed baseline e-questionnaires, and a subset (*n* = 13) took part in separate virtual fuzzy cognitive mapping sessions.

Impact on Practice or Results

Participant perceptions of TE and RC during the active COVID-19 phase were high with mean ratings of M = 4.48; SD = 0.70 for TE and M = 3.76; SD = 0.76 for RC (rated from 1 to 5). No significant differences in perceptions of TE and RC were found across the four groups of participants. Patient perceptions of TE and RC were highly correlated with their care satisfaction (TE r = 0.86; RC r = 0.66; *p* < 0.01).

Discussion or Conclusions

To our knowledge, this study is the first to simultaneously gather perspectives from various stakeholders on cancer team functioning during the pandemic. The finding that patient perceptions of team functioning is closely linked to care satisfaction underscores the importance of involving patients in continuous quality improvement initiatives.

89

### 17.4. Optimization of Patients’ Surgical Trajectory within a Pulmonary Cancer Network

Gabrielle Chartier ^1^, Nathalie El-Haddad ^1^, Chelsea Ilagan ^2^, Vanessa Lewis ^1^, Renata Benc ^1^, Karine Lepage ^1^, Christine Bouchard ^2^, Catherine Bigras ^1^ and Carmen Loiselle ^3^

1. CIUSSS du Centre-Ouest-de-l’Île-de-Montréal, Montreal, Canada
2. Centre universitaire de santé McGill, Montreal, Canada
3. Department of Oncology and Ingram School of Nursing, Montreal

Background/Rationale or Objectives/Purpose

The Quebec’s Minister of Health and Social Services has made improving access to cancer treatment one of their main priorities. In our setting, patients seen at the pulmonary clinic are transferred to another institution for surgery, then returned to our clinic. This process tends to increase complexity in care and in patient/family experiences. Therefore, we decided to initiate a Kaizen-driven project to standardize processes and enhance staff and patient experiences and communication across care transitions.

Methodology or Methods

In addition to feedback received from participants prior to a 3-day workshop (*n =* 10), in-depth interviews were conducted with pulmonary care team members (*n* = 6) at the clinic and patients (*n* = 2) who had undergone transfer for lung cancer surgery. Using the d’Amour (2004) framework and LEAN principals, participants were released to attend a workshop for three days. Day one served to map the current state of care processes, day two focused on desired and targeted outcomes, and day three served to formalize an action plan to optimize care pathways.

Impact on Practice or Results

Three themes emerged from the interactive workshop: communication processes within the surgery site, further defining the various nursing roles (4), and communication processes related to imaging. A co-designed action plan with key interventions is currently being implemented over an eight-week period with 80% of the plan already operationalized.

Discussion or Conclusions

This collaborative initiative has streamlined administrative processes, and increased the fluidity of patient transfer processes. A continuous quality improvement plan is set up to ensure sustainability of this initiative.

## 18. Final Category: Q. Patient Oriented Research Approaches

53

### 18.1. Canadian Lung Cancer Patient Coping with Fear and Anxiety about the Cancer Recurrence: Needs and the Extent to which They Are Being Met

Christine Qiong Wu ^1^, Parisa Sepehri ^2^, Angus Pratt ^3^, MaryAnn Bradley ^4^, Tim Monds ^5^, Catherine Larose ^6^, Christina Sit ^7^ and Andrea Redway ^8^

1. Canadian Lung Cancer Advocacy—Breathe Hope, Winnipeg, Canada
2. Department of Psychology, Concordia University, Montréal, Canada
3. Canadian Lung Cancer Advocacy—Breathe Hope, Vancouver, Canada
4. Canadian Lung Cancer Advocacy—Breathe Hope, Toronto, Canada
5. Canadian Lung Cancer Advocacy—Breathe Hope, Edmonton, Canada
6. Canadian Lung Cancer Advocacy—Breathe Hope, Montreal, Canada
7. Leukemia and Lymphoma Society of Canada, Toronto, Canada
8. Canadian Lung Cancer Advocacy—Breathe Hope, Ottawa, Canada

Background/Rationale or Objectives/Purpose

Many lung cancer patients experience Fear or Anxiety about the Recurrence of Lung Cancer (FARLC). Extant research on cancer suggests that support for FARLC is the most significant and unmet need. We aimed to assess which types of support are needed and desired and to what extent lung cancer patients’ needs are being met.

Methodology or Methods

Together with the Canadian Lung Cancer Advocacy—Breathe Hope, a purpose-designed questionnaire was distributed online via e-mail, newsletters and social media in Canada. All questions were multiple-choice.

Impact on Practice or Results

Out of 121 respondents, 119 had experienced FARLC and were included in the present study. While over 82% of respondents reported having access to mental healthcare services, 67% of patients reported that they were never referred to mental health services by their oncology team. The most important coping strategies were (1) talking to someone about FARLC (71%), (2) distracting oneself with enjoyable activities (61%), and (3) gaining practical tips on dealing with FARLC (50%). The most influential systems of support identified were patient support groups online and in person (65%), psychologists/therapists within the hospital/cancer care centre (47%), and friends, family and co-workers (42%).

Discussion or Conclusions

Most lung cancer patients reporting FARLC indicated a need for more support, but the majority are not receiving information or referrals for mental health support from their oncology care teams. Many patients rely on patient support groups, friends and family for support. Serious gaps in the mental health support available to lung cancer patients need to be addressed.

80

### 18.2. When We Hear “Cancer”: Patients’ Psychosocial Needs during Diagnosis

Leah Stephenson ^1^ and Kathleen Barnard ^2^

1. All.Can Canada, Toronto, Canada
2. Save Your Skin Foundation, Penticton, Canada

Background/Rationale or Objectives/Purpose

- Share the current state of patients undergoing a cancer diagnosis, particularly psychosocial support needs as soon as the word “cancer” is mentioned, based on research commissioned by All.Can Canada (ACC);
- Share a patient story that illustrates the challenging current state of cancer diagnosis;
- Identify in small groups any known practices that connect people undergoing investigation for cancer to psychosocial support;
- Explore these practices in the plenary session.

Methodology or Methods

A researcher conducted a literature review, 30 qualitative interviews with cancer survivors, and a healthcare providers’ survey to better understand the Canadian diagnosis landscape. ACC’s patient-led, multi-stakeholder Interim Steering Committee oversaw the methodology, implementation, and interpretation. Findings are being shared with leading cancer, primary care, and health policy stakeholders across Canada.

Impact on Practice or Results

This interactive workshop aims to increase participants’ awareness of the importance of psychosocial support during cancer diagnosis plus explore existing practices that are making a difference. A summary will be shared post-workshop with interested participants via email.

Discussion or Conclusions

ACC recommends to: “expand availability and accessibility of psychosocial supports for people going through cancer diagnosis and create linkages between cancer care and supportive care”. Key enablers are to:

- Work with national bodies with relevant mandates to grow psychosocial supports for people going through cancer diagnosis across Canada;
- Increase awareness of existing psychosocial supports and create mechanisms for providers to easily connect patients with available resources;
- Embed psychosocial supports into community-based primary care services, diagnostic facilities, and hospital settings;
- Fund travel and other supports for people living in rural and remote regions.

82

### 18.3. Psychosocial Support Needs and Contextual Factors for Patients with Genetic Cancer Susceptibility: Learning from Patients’ Lived Experiences and Application to Co-Design of a Patient Decision Aid

Kelly Kohut ^1,2^, Kate Morton ^1^, Lesley Turner ^1^, Diana Eccles ^1^ and Claire Foster ^1^

1. University of Southampton, Southampton, UK
2. St George’s University Hospitals NHS Foundation Trust, London, UK

Background/Rationale or Objectives/Purpose

Patient decision aids (PtDAs) complement shared decision-making with healthcare professionals, improve decision quality and save clinic time. However, PtDAs often lack theoretical underpinning. We are co-designing a PtDA to help people with genetically increased cancer risks manage choices. We drafted a logic model based on published frameworks but had not engaged with people who will use the PtDA.

Methodology or Methods

Patients were asked in an interactive workshop what is important to them when they make health decisions, what theoretical constructs are most meaningful and how this should be applied to the co-design of a PtDA. They were asked to lead small group discussions about lived experiences. An artist created a live visual summary. Notes from patient discussions and artwork were analysed using reflexive thematic analysis.

Impact on Practice or Results

The overarching theme was that it’s personal. Contextual factors were varied and changed with age and life situation. There was no one ‘best fit’ theory to target support needs and psychosocial issues, suggesting an inductive, flexible framework approach to programme theory would be most effective. The PtDA logic model was revised based on patient feedback.

Discussion or Conclusions

Meaningful co-design of PtDAs is likely to lead to improved patient care through understanding the intricately personal nature of health decisions, and tailoring content and format for holistic care. Psychosocial support needs are common and varied. Patients will share these needs when given the time, space and trust to do so, requiring collaborative effort and a commitment from clinicians to promote shared decision-making.

84

### 18.4. How Do Patients Want to Receive Information and Advice about Modifiable Diet and Lifestyle Factors to Lower Their Chances of Getting Cancer in the Future?

Kelly Kohut ^1,2^, Kate Morton ^1^, Lesley Turner ^1^, Diana Eccles ^1^ and Claire Foster ^1^

1. University of Southampton, Southampton, UK
2. St George’s University Hospitals NHS Foundation Trust, London, UK

Background/Rationale or Objectives/Purpose

People with an increased chance of getting cancer due to family history or a pathogenic variant in a cancer susceptibility gene are often given advice about diet and lifestyle changes that could lower risk. Based on our Patient and Public Involvement work so far, we have learned that patient preference for the amount of information and the way this is presented can be highly personal and emotive.

Methodology or Methods

A patient-oriented Q&A session to ask the following questions for which we are seeking feedback to inform co-design of a patient decision aid (PtDA) (personalised, interactive website and booklet) about cancer risks and management options. (1) Should a PtDA include information about diet and lifestyle factors? (2) Digital polls will be presented displaying options asking about the type of information that should be displayed in a PtDA and the language used. (3) Patients and providers will be asked how important they feel it is to control diet and lifestyle factors, and how it makes them feel to receive advice from healthcare professionals about this.

Impact on Practice or Results

Feedback will be considered using the Person-Based Approach to make iterative improvements to the draft PtDA to optimise acceptability and usefulness for patients.

Discussion or Conclusions

Co-design of PtDAs with the people who will use these resources is an approach that should be championed from conception of research through co-production to clinical implementation and evaluation. Regular review and updates should be made based on patient feedback. Attention should be given to equality, diversity and inclusion to partner with underserved populations.

97

### 18.5. Participatory, Patient-Oriented Research to Reshape Adolescent and Young Adult Cancer Care

Cheryl Heykoop, Tiffany Hill, Jennifer Wolfe, Lily Rogers, Param Gill, Ada Okonkwo and Jon Avery

Royal Roads University, Victoria, Canada

Background/Rationale or Objectives/Purpose

The Anew Research collaborative uses patient-oriented, participatory action research to partner with AYAs with cancer and cancer care allies (supporters, clinicians, decision-makers, researchers) to better understand lived experiences, needs, and priorities of cancer care for AYAs and co-create tangible changes for AYA cancer care.

Methodology or Methods

In this participatory workshop, we will explore patient-oriented, participatory action research and how it is contributing to change in AYA cancer care. Participants will: learn more about patient-oriented, participatory action research methods and how Anew is applying them in practice, will experience creative methods to facilitate patient-engagement in research, and will discuss in small groups how patient-oriented, participatory action research methods and approaches can be applied in research and clinical contexts and how they might support greater representation and equity.

Impact on Practice or Results

Through the application of patient-oriented, participatory action research, we have found AYAs have opportunities to share their own experiences and perspectives, engage with clinicians and care providers, and play a more active role in advocacy and support for AYAs. Patient engagement also supports more responsive, relevant programming and has the potential to improve health outcomes.

Discussion or Conclusions

Participants who attend this workshop will be able to: (1) better understand patient-oriented, participatory action research and how it can be applied in practice; (2) consider relevant ethical considerations; (3) consider how to apply patient-oriented, participatory action research methods within their own practice; (4) consider how patient-oriented, participatory action research methods can help support greater equity and representation. Together, we can reshape cancer care for all AYAs!

## 19. Final Category: R. Other Value-Based and Person-Centered Cancer Care

28

### 19.1. Patients’ Preferences for Health Management Support Strategies during and after Treatment for Lung, Breast, Colorectal, Prostate, or Ovarian Cancer: An Australian Cross-Sectional Survey

Anna C Singleton ^1^, Nashid Hafiz ^1^, Raymond Chan ^2^, Amy Von Huben ^1^, Rodney Ritchie ^3^, Nikki Davis ^4^, Kirsty Stuart ^5,6^, Aaron Sverdlov ^7,8^, Rebecca Raeside ^1^, Karice K Hyun ^9,10^, Stephanie R Partridge ^1^, Elisabeth Elder ^1,5^ and Julie Redfern ^1,11^

1. The University of Sydney, Sydney, Australia
2. Caring Futures Institute, Flinders University, Adelaide, Australia
3. Male Breast Cancer Global Alliance, Sydney, Australia
4. Primary Care Collaborative Cancer Clinical Trials Group Consumer Advisory Group, Melbourne, Australia
5. Westmead Breast Cancer Institute, Sydney, Australia
6. Crown Princess Mary Cancer Centre, Sydney, Australia
7. University of Newcastle, Newcastle, Australia
8. John Hunter Hospital, Newcastle, Australia
9. University of Sydney, Sydney, Australia
10. Concord Repatriation General Hospital, Sydney, Australia
11. University of New South Wales, Sydney, Australia

Background/Rationale or Objectives/Purpose

To understand people’s preferences for health management, and technology support and distribution strategies during and post-cancer treatment.

Methodology or Methods

Australian adults with a history of breast, colorectal, lung, ovarian or prostate cancer were recruited through paid Facebook advertisements and cancer not-for-profit e-newsletters to complete a cross-sectional, consumer/researcher co-designed, online survey (34-items). Quantitative data were summarized using summary statistics (means/standard deviations (SD); frequencies/percentages). Free-text responses were analyzed thematically.

Impact on Practice or Results

Most participants were Caucasian (487/546; 89%), female (72%), diagnosed with early-stage (I–III) disease (78%) at Mage = 59 years (SD = 10 years; range 21–83 years), diagnosed with breast (125/524; 23%), colorectal (20%), lung (17%), ovarian (19%) and prostate cancer (19%). The proportion of participants reporting they did not receive enough information during versus post-treatment was 147/446 (33%) and 169/320 (53%), respectively. During treatment, participants wanted information about free programs/services (226/546; 41%) and self-management (222/546; 41%). After treatment, fear of recurrence (200/546; 37%) and managing side effects (199/546; 36%) were most common. The same three themes arose between treatment times (during versus post), sex, cancer type and stage: (1) seek support immediately from clinicians, loved ones and/or patients (e.g., support groups), (2) exercise and (3) practise self-care. Participants desired websites, apps or text messages with an ‘ask-an-expert’ function. Most felt these services should be government-funded (365/546; 65%), and promoted by health-professionals (314/546; 57%) or not-for-profit cancer websites (251/546; 46%).

Discussion or Conclusions

Participants had more unmet psychosocial needs post-treatment versus during treatment. Participants suggested that psychosocial support, exercise, and direct contact with clinicians via free-of-charge technology may be useful.

33

### 19.2. Engaging Patients with What Matters Most to Them, Use of Patient Reported Outcomes in Cancer Care

Renata Benc ^1^, Catherine Bigras ^1^, Karine Lepage ^1^, Carmen Loiselle ^1,2^ and George Loutochin ^3^

1. CIUSSS du Centre-Ouest-de-l’Île-de-Montréal, Montreal, Canada
2. Ingram School of Nursing McGill University, Montreal, Canada
3. ROSSY cancer network, Montreal, Canada

Background/Rationale or Objectives/Purpose

Patient-reported outcomes (PROs) are crucial in documenting and acting on patients’ needs, concerns, and symptoms, while empowering them to be more involved in their care. The use of PROs in cancer has been found to contribute to enhanced symptom control, health-related quality of life, patient–clinician communication, and patient activation.

Methodology or Methods

Our oncology team developed an electronic version of what we call the “Wellness Questionnaire” which includes standardized PROs measures (i.e., ESAS-R, Canadian Problem checklist and the Distress thermometer), with secondary assessments (BPI, CFS, PHQ-8, GAD 7) when relevant.

In a pilot project, patients (*n =* 200) newly seen at oncology clinics completed the online Wellness Questionnaire prior to their treatment visit. The nursing team reviewed PROs’ summary reports while patients received treatment, guiding interventions specific to the reported needs. For instance, if symptomatology was reported, patients received symptom management online tip sheets developed by our team. According to patient demand, a tip sheet on body image issues was added to the list of resources.

Impact on Practice or Results

Nursing-specific interventions included active listening (e.g., validating concerns, fears), providing informational materials for self-care, and referrals to psychosocial or other services when required.

Results trending over time through visits, demonstrated that depression, anxiety, fatigue and pain decreased by 36%, 44%, 44%, and 28%, respectively.

Discussion or Conclusions

Future directions include addressing issues in follow ups, broader implementation of the Wellness questionnaire across tumor sites, and integration into routine practice.

77

### 19.3. “How Can We Work Together to Make [HEALTHCARE] better?”: Exploring the Perspectives and Experiences of Cancer Patients Involved in a Patient as Teacher Program in Toronto, Ontario

Cheryl Pritlove ^1,2^, Beth Edwards ^1^ and Jory Simpson ^1,2^

1. Unity Health Toronto, Toronto, Canada
2. University of Toronto, Toronto, Canada

Background/Rationale or Objectives/Purpose

Involving patients in medical education is an important way to foster patient-centered care competencies for future physicians. We sought to explore patients’ perspectives and experiences regarding their participation in a Patient as Teacher (PaT) program.

Methodology or Methods

We recruited 15 participants with varied cancer diagnoses involved in the PaT program, a collaboration between the University of Toronto Faculty of Medicine and St. Michael’s Hospital. We employed semi-structured interviews, including photo-elicitation, to capture motivations for becoming patient teachers, impacts of program participation, and suggestions for program improvements. Data were coded using thematic analysis.

Impact on Practice or Results

Patient teachers were motivated to participate in the PaT program because they believed that the program, and the practice of situating patients at the center of medical education, was key to producing a more caring, culturally and psychologically safe, and collaborative healthcare system. Participants described unexpected benefits to their health and healing through storytelling and development of self-advocacy skills resulting from the bidirectional teaching/learning that occurred between students and teachers. For many, this contributed to increased confidence and self-efficacy to engage in clinical encounters. Importantly, there was an expressed need to acknowledge and overcome barriers to diverse representation of patient teachers to maximize program impact.

Discussion or Conclusions

The PaT program situates patients as experts, shifting traditional medical pedagogies that privilege the physician perspective. Findings indicate that the PaT program offers an important counterbalance to existing medical school curriculum in ways that help to prioritize patient-centered outcomes, and facilitates healing and self-advocacy for patient teachers.

83

### 19.4. Contributions of Shared Decision Making to the Implementation of Value-Based Healthcare (VBHC) across the Colorectal Cancer Care Trajectory

Jade Marcotte ^1,2^, Sixtine Assalé ^1,2^, Karine Lepage ^1,2^, Kim Ma ^1,2^, Eva Villalba ^3^, Monika Slovinec D’Angelo ^3^ and Aurelia Di Fabrizio ^2^

1. Jewish General Hospital, Montreal, Canada.
2. Integrated Health and Social Services Network for West Central Montreal, Montreal, Canada.
3. Quebec Cancer Coalition, Saint-Lambert, Canada

Background/Rationale or Objectives/Purpose

The Value-based healthcare (VBHC) framework implementation in the colorectal cancer care trajectory sought to optimize care delivery, improve patients’ outcomes, and reduce patients’ burden of disease. Shared decision making (SDM) supports VBHC objectives by encouraging patients’ involvement in decision-making about their care through open and informed discussions with the care team. SDM focuses on evidence-based practices tailored to patients’ characteristics, such as physical condition, preferences, values, and experience.

Methodology or Methods

As a quality improvement project, an informational video was created and will be broadcast in waiting rooms in the oncology departments to enhance care recipients’ involvement in their care. This video includes the goals and benefits of SDM, the advantages of responding to the patient-reported outcomes (PROs) questionnaire, and completing the Whiteboard (an inpatient communication tool to engage patients, their family members, and clinicians). Based on these reported outcomes, care plans can be readily adjusted to further meet patients’ needs.

Impact on Practice or Results

SDM is being incorporated into care delivery through patient–clinician interactions based on the PROs questionnaire and/or Whiteboard answers. Consequently, patients’ care plan can be adjusted to their physical condition, preferences, values, life circumstances and experience. SDM is expected to improve patients’ care experience, their involvement in the decision-making process as well as the completion and the integration of PROs questionnaire and the Whiteboard into clinicians’ practices.

Discussion or Conclusions

The SDM implementation requires education and commitment of clinicians and patients. We will assess and compare outcomes (patients’ health outcomes, tools’ completion rate) before and after the SDM video broadcast.

115

### 19.5. Integrating a Patient-Driven Psycho-Oncology Service into the Cancer Care Workflow: An Overview of Electronic Referral and Patient Engagement Patterns

Alyson Moadel-Robblee, Brittany Miller, Johnna Bakalar, Tasmia Kabir and Shalom Kalnicki

Montefiore Einstein Cancer Center, Bronx, USA

Background/Rationale or Objectives/Purpose

A psycho-oncology program known as “BOLD” offering counseling, peer navigation, and support groups at no cost, has served as an independent, onsite resource to a NYC cancer center serving a highly diverse and disadvantaged community since 2008. In 2019, a Quality Improvement project to integrate BOLD into the cancer care workflow through its electronic medical records was undertaken. Patterns of referral and engagement are described.

Methodology or Methods

Between Jun 2020 and Dec 2022, oncology providers made 1324 referrals to BOLD, with mean monthly referrals increasing by year from 17 to 42 to 58. To date, 298 (23%) patients have agreed to intake and were 2.8 Mean years from diagnosis, on treatment (44%), with 49% having breast cancer. Patients were primarily Hispanic (44%), Black (40%), female (82%), and a Mean age of 57 (range 21–83). Based on the Distress Thermometer, 74% met clinical levels of distress.

Impact on Practice or Results

Interest in support groups (74%), counseling (45%), and a BOLD Buddy (45%) was high. Women expressed greater interest in support groups (77% vs. 65%, *p* < 0.059), counseling (49% vs. 28%, *p* < 0.02) and a Buddy (48% vs. 31%, ns). Those with high vs. low distress had more urgent need for counseling (18% vs. 6%; *p* < 0.05) but comparable interest in a Buddy (47% vs. 41%). Remarkably, 52% of patients were interested in volunteering.

Discussion or Conclusions

Integrating psycho-oncology services into standard cancer care is feasible and patient-centric. Provider referral patterns and high interest among patients to volunteer bode well for growth and sustainability. Next steps will focus on engaging men and measuring impact.

118

### 19.6. Experience OnCourage—The Missing Piece to Your Support System. OnCourage Is a Bilingual Website Platform Designed to Deliver Information and Receive Support from Family and Friends

Debbie Magwood and Maggie Costa

WICWC, Kirkland, Canada

Background/Rationale or Objectives/Purpose

OnCourage was designed to fulfill a need!

OnCourage is a bilingual website platform designed to deliver information and receive support from family and friends of a loved one’s cancer journey, building a solid support community.

Having cancer can be a full-time job. Keeping family and friends in the loop is a big part of the cancer journey, which can be time-consuming and exhausting. Adding to the demands from the doctors, scanning procedures, and treatment visits, all while maintaining daily activities (laundry, cooking, household maintenance, groceries, etc.) can be strenuous and affect the overall wellness of the cancer patient.

OnCourage gives you the break you need! It lets you create a customized, private webpage where family and friends can “gather” for updates, share stories, and support you or your family member diagnosed with cancer.

OnCourage makes it simpler! You can post news and updates for the family members and friends you invited to join your page in one place. They will receive real-time updates and can share their thoughts, stories, and pictures on the page.

Methodology or Methods

In 2021, a feasibility assessment took place whereby 40 individuals were interviewed to assess if this platform is needed. The results were a resounding YES. The suggestions were added to the development of the platform.

In early 2022, we selected 20 families to become part of our testing group to ensure the platform’s efficacy.

Impact on Practice or Results

In 2023 the platform was launched (soft launch) and will be continued to be rolled out across Canada.

Discussion or Conclusions

Experience the platform.

125

### 19.7. Psychosocial Oncology Integrating Promising Upstream Approaches of Co-Design, Value-Based Health Care, and Strengths-Based Nursing and Health Care

Carmen Loiselle

McGill University, Montreal, Canada

Background/Rationale or Objectives/Purpose

Psychosocial oncology quickly embraced person-centred care (PCC)—defined as a respectful, responsive, and tailored approach to care that embeds peoples’ needs, values, and preferences. More recently, complementary upstream approaches have been proposed—those that consider the personal, social, economic, and environmental contributors to health and well-being. These contributors can readily be addressed through intervention co-design, value-based health care (VBHC), and strengths-based nursing and health care (SBNH).

Methodology or Methods

Herein, a framework integrating these three upstream approaches is proposed.

Impact on Practice or Results

The integrative framework seeks to guide cancer care practices by directly addressing relevance, sustainability, optimal resource allocation, and equity in cancer care delivery.

Discussion or Conclusions

Moving forward, psychosocial oncology must ensure that integrative approaches such as the ones proposed here are clearly operationalized and evaluated periodically in terms of both intended and unintended effects. As dynamic and committed forces, organizations such as the Canadian Association of Psychosocial Oncology (CAPO) and the Canadian Association of Nurses in Oncology (CANO) are strategically positioned to spearhead similar efforts to ensure optimization of cancer care services and cancer-related outcomes.


**Author Name**

**Program Codes**
on behalf of the MEGAN-CAN team19Abou Chaar, Emile44, 49Aguiar, Stefan129Ahmed, Saima76, 78, 79Ajith, Harsha51Al-Awamer, Ahmed45Albert, Justine65Alexander, Sarah101, 102Ali, Alias27Alibhai, Shabbir96Alonzi, Sarah87Arab, Marianne30, 57, 58, 100Archambault-Grenier, Marie-Anne73Arenburg, Maggie57Asada, Yukiko110Assalé, Sixtine83Assan, Odilon127Atenafu, Eshetu96Attieh, Samar8, 54, 55Attiya, Ameer88Aubrey-Bassler, Kris51Avery, Jonathan22, 45, 93, 97Babinski, Stephanie67, 63Bakalar, Johnna94, 108, 115Bansal, Mannat15, 92, 99, 123Barbeau, Anne129Bardine, Alexei71Barnard, Kathleen80Bauereiß, Natalie35Baydoun, Mohamad9, 114Bean, Sally62Bedran, Michelle38Belzile, Eric88Ben-David, Shelly2Benc, Renata33, 64, 79, 89Bender, Jacqueline63, 59, 67, 75Bennett, Kathleen124, 128Berbiche, Djamal23Bergeron, Catherine71Berlin, Alejandro63Bernatchez, Marie-Solange64Bernstein, Lori96Bigras, Catherine33, 64, 89Bilash, Tristan12Bilodeau, Karine116Birnie, Kathryn114Bisaillon, Laurie5Bonanno, Marco13Booth, Mary-Clare71Bouchard, Christine64, 89Bourque, Claude Julie13Bourque, Michaela A.19, 20Bradley, Holly95, 103Bradley, MaryAnn53Bridel, William92Brotherton, Julia60Browne, Sondria37Bryant-Lukosius, Denise26Bultz, Barry D46Burnett, Laura45, 95, 103, 117Cahir, Caitriona124, 128Caissie, Amanda100Campbell, Allie56Campbell, Kristin45Canton, Kyle107Caplette-Gingras, Aude48, 61Capozzi, Lauren C.98Carlson, Linda9, 114Carpenter, Travis17Carter, Nancy26Chahine, Georges38, 39, 43, 44, 49Chalifour, Karine111Chan, Raymond28Chan, Wing C.20Chang, Eugene96Chartier, Gabrielle79, 89Chen, Lawrence25Chiquette, Jocelyne48Chisholm, Sue100Cho, Sara105, 106Chomistek, Tessa105Chu, Yun-Ting Paige62Cil, Tulin32Climans, Seth22Coaston, Troy87Coburn, Natalie20Colwell, Bruce100Costa, Maggie118Covelli, Andrea32Cripps, Hannah99Croke, Jennifer73, 75Culos-Reed, S. Nicole15, 42, 92, 99, 98, 123Cury, Fabio25Daley, Ellen60Daniel, Juliet32Danyluk, Jessica98Darani, Shaheen81Daun, Julia T.15, 98, 99David, Pierre-Marie126Davidson, Dr. Kassim4Davis, Laura E.20Davis, Nikki28deBruin, Marijn127Deeks, Julia95DeIure, Andrea46Deleemans, Julie19, 20Dell’Angelica, Derek87Desjardins, Leandra13Devaraja, Kaviya22Dhruva, Tana42, 98, 99Di Fabrizio, Aurelia83Dickson, Lindsay30Digout, Carol56Donnan, Jennifer29Dorval, Michel48, 128Dow-Fleisner, Sarah2, 31Downar, James27Dreger, Julianna15, 99Drummond, Rachelle17, 107Easaw, Jacob C.98Easley, Julie63Eccles, Diana82, 84Edwards, Annemarie45Edwards, Beth77Einstein, Gillian85El-Haddad, Nathalie89Elder, Elisabeth28Elterman, Dean91Embrett, Mark58Erez, Guy79Eskander, Antoine20Esplen, Mary Jane Esplen67, 81Ester, Manuel15Fares, Elyssa43Fay-McClymont, Taryn86Fergus, Karen24Fidler-Benaoudia, Miranda105Fifield, Carolyn100Filion, Catherine48Fitzgibbon, Kyle101Flanders, Annette56Fletcher, Sarah111Flett, Gordon61Foldes-Busque, Guillaume72Forbes, Caitlin46, 107Forbes, Robin113Foster, Claire82, 84Francis, George J.98Fung, Tak106Fusaro, Maria59Gajtani, Zen114Ganann, Rebecca26Garland, Sheila29, 37, 50, 51, 68Gauthier, Lynn72Giles, Jennifer90Gill, Param93, 97, 111Gobeil, Elaine98Goddard, Karen45Gotay, Carolyn127Gottheil, Chiara14Gouin, Jean-Philippe61Gradstein, Roetka56Greeley, Krista68Griffin-Mathieu, Gabrielle60, 74Guilcher, Gregory86, 106Guillaumie, Laurence126, 127Guimond, Anne-Josée72Guirguis, Steven91Gunther, Kevin14Gupta, Abha45Hachem, Roula38, 39, 43, 44, 49Hafiz, Nashid28Hagopian, Emma129Hajj, Aline38, 39, 43, 44, 49Hales, Sarah35, 62Hallet, Julie20Hannon, Breffni45, 62Harb, Sami24Harb, Sarah49Harding, Scott V51Harris, Nicholas29Hasanabadi, Hamidreza6Hatami, Mohammad6Haward, Ben60, 74Henry, Brianna90, 107Henry, Melissa25, 27Hercules, Shawn75Heykoop, Cheryl93, 97, 111, 112Hier, Michael25Hill, Tiffany93, 97, 111Ho, Certina81Hollenhorst, Helmut57, 58, 100Holme, Elizabeth117Horta, Lacey103Hou, Sharon106, 107Howard, Fuchsia45Howes, Janice57, 58Hyun, Karice K28Ilagan, Chelsea89Incze, Taylor91Isabelle, Marcoux27Isenberg-Grzeda, Elie62Iyengar, Yajur22Jamieson, Kara57Janzen, Anna99Jeanty, Maya64Jeyapalan, Apiramy117Jibb, Lindsay101, 102Jobin-Théberge, Adèle72Jones, Jennifer96Justine, Albert27Kaal, Katrin Julia56, 57, 58, 100Kabir, Tasmia115Kalnicki, Shalom115Kaluznick, Georgia99Karkaby, Laurice35, 85Kattan, Joseph38, 43, 49Kelly, Brian46Kennedy, Katie59Kennedy, Margo73, 75Kenny, Sarah J.42Kerr, Leslie16Khalil, Ielaf32Khoury, Rita38, 39, 43, 44, 49Klein, Roberta Y.129Kohut, Kelly82, 84Kozloff, Nicole81Krull, Kevin86Kumar, Rajat96Kusaian, Tina64Kwong, Danica81Körner, Annett71Labbé, Angéline126Lafay-Cousin, Lucie86Lambert, Leah111Lambert, Sylvie88Lanctot, Krista81Langelier, David M.42, 96Langlois, Frédéric61Larose, Catherine53Lau, Paige75Lauzier, Sophie5, 126, 127, 128Laverdière, Isabelle126Lawrence, Logan58Lee, Rachel29Lee, Sharon95Lemieux, Julie61, 127Lemy Dantica, Joséphine64LePage, Karine33, 64, 83, 89, 79Lesiuk, Christine98Lessard, Sylvie23Levesque, Ariane13Lewis, Chris56Lewis, Vanessa89Li, Madeline27, 62, 113, 129Link, Claire46Litvin, Alon79Liu, Jun27Lofters, Aisha32, 60Loganathan, Pragash113Loiselle, Carmen G.8, 54, 55, 76, 78, 79, 33, 65, 89, 125Lopez, Paty96Loutochin, George33Lovas, Mike113Lu, Shuang46Lush, Mackenzie29Luu, Thompson15Lynn, Gauthier27Lyver, Brendan73, 75Ma, Kim83Macedo, Alyssa113MacRae, Helen92Magwood, Debbie7, 118Mahar, Alyson L.19, 20Malakian, Argin101Malfitano, Carmine35, 101, 102Mangerpan, Jeff64Marcotte, Jade83Martinez, Christina108Massicotte, Véronique61, 72Mather, Haley114Mathews, Angela101Matthew, Andrew91Mattsson, Jonas96Mayrand, Marie-Hélène60Mazoff, Carrie64Mazzurco, Olivia129Mc Brearty, Claudia5, 72McBride, Emily60McCurdy, Arleigh109McDonough, Meghan H.15, 98McGee, Sharon63McLaughlin, Emma98, 99McLennan, Andrew9McNeely, Margaret L.15, 98McTaggart-Cowan, Helen111Meaney, Michael25Memoli, Victoria127Meyers, Logan59Mian, Hira109Michara, Brian27Michel, Alexandra48Miller, Brittany94, 108, 115Moadel-Robblee, Alyson94, 108, 115Molina, Heather99Molnar, Danielle61Monds, Tim53Moody, Lesley113Morhun, Janelle106Morrison, Margaret Ann57, 58, 100Morton, Kate82, 84Mosher, Pamela45Mrad, Hazar116Myers, Charlotte124, 128Nabi, Hermann128Naganathan, Gayathri32Nanos, Stephanie101, 102Nasr, Fady39, 44Neil-Sztramko, Sarah26Nissim, Rinat35, 62Nneke, Kelechi4Nugus, Peter95O’Brien, Suzanne8O’Connell, Sarah16O’Donnell, Kieran25O’Grady, Alice112O’Keeffe, Nathan16Okonkwo, Ada93, 97, 111Olivier D’Avignon, Marianne126Omidbeygi, Maryam6Omobhude, Favour90Onwukeme, Ifeyinwa4Ospina, Paula A.98Ould Brahim, Lydia88Panjwani, Aliza129Papadakos, Janet67, 113Partridge, Stephanie R28Passes dos Santos, Raissa95Peltz, Sarah91Perez, Citlali87Perez, Samara60, 74Perrault, Sébastien13Perski, Olga67Piedalue, Katherine-Ann50Porter, Geoff110Potts, Henry67Pratt, Angus53Pritlove, Cheryl77, 109Qi, Siwei46Rabbaa Khabbaz, Lydia38, 39, 43, 44, 49Racine, Nicole106Raeside, Rebecca28Rahamatullah, Iqra105, 106Ramasamy, Mathankki109Ramirez Garcia, Maria-Pilar116Rana, Benny92, 99Rash, Joshua37Redfern, Julie28Redway, Andrea53Reynolds, Kathleen90, 107Ringash, Jolie59Ritchie, Rodney28Roberts, Araby86Robichaud, Lye-Ann13Robichaud, Pamela57Rodberg, Danielle70Rodin, Danielle32, 63Rodin, Gary35, 101, 102, 129Rogers, Lily93, 97, 112Roldan Urgoiti, Gloria98Rosberger, Zeev25, 60, 74Roy, Charlotte13Russell, K Brooke46Rydall, Anne35, 101, 129Ryder-Burbidge, Charlotte105Saab, Nathalie44Sacre, Hala38, 39, 43, 44, 49Sadeghi, Nader25Salameh, Pascale38, 39, 43, 44, 49Sampalli, Tara58Sandhu, Irwindeep109Savard, Josée5, 37, 48, 61, 72, 128Savas, Sevtap12, 55Sayeed, Sobia87Schulte, Fiona17, 46, 86, 90, 105, 106, 107Schulz-Quach, Christian73, 75Scruton, Sarah56, 63, 67Seal, Melanie37Selby, Debbie62Sellar, Christopher98Sepehri, Parisa53Shapiro, Gilla73, 75, 129Sharma, Ramona31, 70, 130Silvius, James46Simpson, Jory77Singleton, Anna C28Sit, Christina53Slovinec D’Angelo, Monika83Smith, Laurie60Soobiah, Charlene67Springer, Leila32Squires, Lauren63, 67, 75Srivastava, Richa113Stanton, Annette L.87Stanwood, Matthew57Stephenson, Leah80Stere, Alison62Stokoe, Mehak86, 90Stonebridge, Genevieve112Stragapede, Elisa91Struik, Laura2, 31, 70, 130Stuart, Kirsty28Sultan, Serge13Sultanem, Khalil25Sundstrom, Alyssa2Sussman, Jonathan63Sutradhar, Rinku20Sverdlov, Aaron28Tam, Samantha96Tarasuk, Joy57, 58, 100Tatar, Ovidiu60, 74Teggart, Kylie26Teshima, John81Thatcher, Fiona112Thibodeau, Kimberley12, 55Thoms, John37Thurston, Chantale12Tomblin Murphy, Gail58Torchetti, Tracy117Tremblay, Dominique23Tricco, Andrea67Trottier, Amy56Tulk, Joshua37, 51, 68Turcotte, Véronique127Turner, Lesley82, 84Tutelman, Perri105ulthiin, iowyth hezel75Urquhart, Robin14, 37, 56, 58, 63, 110Vaezi, Amirabbas6VandeZande, Danielle103Vanessa, Bisson-Gervais27Vickery, Jessica110Villalba, Eva83Villeneuve, Stephanie56Von Huben, Amy28Wagoner, Chad W.42Wahoush, Olive32Wakeham, Bob51Wasserman, Sydney88Wassersug, Richard14Watson, Linda46Weigler, Will112Wells, Greg D.16Werstuik, Saige2West, Sarah L.16Wexler, Megan113Wigle, Jannah109Williamson, Tanya98Wilson, Lianne103Witteman, Holly67Wolfe, Jennifer93, 97, 111, 112Wong, Geoffrey63, 67Wong, Jiahui81Woodside, Melanie59Wright, Frances32Wu, Christine Qiong53Wurz, Amanda99Yamoah, Makaela87Yang, Lin92Yeates, Keith86Yi, Yanqing51Yu, Ally102Zeitouni, Anthony25Zhang, Andy22Zheng, Runqun Helen62Zhu, Patricia60, 74Zimmermann, Camilla101, 102Zwicker, Hailey106

